# Supplementary material for: Marine-Derived Penicillium purpurogenum Reduces Tumor Size and Ameliorates Inflammation in an Erlich Mice Model
Source: Mar Drugs. 2020 Oct 29;18(11):541. doi: 10.3390/md18110541 (PMC7694122; doi:10.3390/md18110541)
Supplement: Supplementary file 1 [file marinedrugs-18-00541-s001.pdf]

Supplementary data content page

Title: Kurilosides A_1, A_2, C_1, D, E and F – triterpene glycosides from the Far Eastern sea cucumber *Thyonidium (=Duasmodactyla) kurilensis* (Levin): structures with unusual non-holostane aglycones and cytotoxicities.

Authors: Alexandra S. Silchenko, Anatoly I. Kalinovsky, Sergey A. Avilov, Pelageya V. Andrijaschenko, Roman S. Popov, Ekaterina A. Chingizova, Vladimir I. Kalinin*

Address: *G.B. Elyakov Pacific Institute of Bioorganic Chemistry, Far Eastern Branch of Russian Academy of Sciences, Pr. 100-let Vladivostoku 159, 690022 Vladivostok, Russia*

Correspondence: kalininv@piboc.dvo.ru; Tel.: +7-914-705-0845

Contents:

Figure S1. The ^{13}C NMR (176.03 MHz) spectrum of kuriloside A_1 (**1**) in C_5D_5N/D_2O (4/1)

Figure S2. The ^{1}H NMR (700.00 MHz) spectrum of kuriloside A_1 (**1**) in C_5D_5N/D_2O (4/1)

Figure S3. The COSY (700.00 MHz) spectrum of the aglycone part of kuriloside A_1 (**1**) in C_5D_5N/D_2O (4/1)

Figure S4. The HSQC (700.00 MHz) spectrum of the aglycone part of kuriloside A_1 (**1**) in C_5D_5N/D_2O (4/1)

Figure S5. The ROESY (700.00 MHz) spectrum of the aglycone part of kuriloside A_1 (**1**) in C_5D_5N/D_2O (4/1)

Figure S6. The HMBC (700.00 MHz) spectrum of the aglycone part of kuriloside A_1 (**1**) in C_5D_5N/D_2O (4/1)

Figure S7. HR-ESI-MS and ESI-MS/MS spectra of kuriloside A_1 (**1**)

Figure S8. The ^{13}C NMR (176.03 MHz) spectrum of kuriloside A_2 (**2**) in C_5D_5N/D_2O (4/1)

Figure S9. The ^{1}H NMR (700.00 MHz) spectrum of kuriloside A_2 (**2**) in C_5D_5N/D_2O (4/1)

Figure S10. The COSY (700.00 MHz) spectrum of the aglycone part of kuriloside A_2 (**2**) in C_5D_5N/D_2O (4/1)

Figure S11. The HSQC (700.00 MHz) spectrum of the aglycone part of kuriloside A_2 (**2**) in C_5D_5N/D_2O (4/1)

Figure S12. The HMBC (700.00 MHz) spectrum of the aglycone part of kuriloside A_2 (**2**) in C_5D_5N/D_2O (4/1)

Figure S13. The ROESY (700.00 MHz) spectrum of the aglycone part of kuriloside A_2 (**2**) in C_5D_5N/D_2O (4/1)

Figure S14. HR-ESI-MS and ESI-MS/MS spectra of kuriloside A_2 (**2**)

Figure S15. The ^{13}C NMR (176.03 MHz) spectrum of kuriloside C_1 (**3**) in C_5D_5N/D_2O (4/1)

Figure S16. The ^{1}H NMR (700.00 MHz) spectrum of kuriloside C_1 (**3**) in C_5D_5N/D_2O (4/1)

Figure S17. The COSY (700.00 MHz) spectrum of the carbohydrate part of kuriloside C_1 (**3**) in C_5D_5N/D_2O (4/1)

Figure S18. The HSQC (700.00 MHz) spectrum of the carbohydrate part of kuriloside C_1 (**3**) in C_5D_5N/D_2O (4/1)

Figure S19. The HMBC (700.00 MHz) spectrum of the carbohydrate part of kuriloside C_1 (**3**) in C_5D_5N/D_2O (4/1)

Figure S20. The ROESY (700.00 MHz) spectrum of the carbohydrate part of kuriloside C_1 (**3**) in C_5D_5N/D_2O (4/1)

Figure S21. 1 D TOCSY (700.00 MHz) spectra of the carbohydrate part of kuriloside C_1 (**3**) in C_5D_5N/D_2O (4/1)

Figure S22. HR-ESI-MS and ESI-MS/MS spectra of kuriloside C_1 (**3**)

Figure S23. The ^{13}C NMR (176.03 MHz) spectrum of kuriloside D (**4**) in C_5D_5N/D_2O (4/1)

Figure S24. The ^{1}H NMR (700.00 MHz) spectrum of kuriloside D (**4**) in C_5D_5N/D_2O (4/1)

Figure S25. The COSY (700.00 MHz) spectrum of kuriloside D (**4**) in C_5D_5N/D_2O (4/1)

Figure S26. The HSQC (700.00 MHz) spectrum of kuriloside D (**4**) in C_5D_5N/D_2O (4/1)

Figure S27. The ROESY (700.00 MHz) spectrum of kuriloside D (**4**) in C_5D_5N/D_2O (4/1)

Figure S28. The HMBC (700.00 MHz) spectrum of kuriloside D (**4**) in C_5D_5N/D_2O (4/1)

Figure S29. 1 D TOCSY (700.00 MHz) spectra of kuriloside D (**4**) in C_5D_5N/D_2O (4/1)

Figure S30. 1 D TOCSY (700.00 MHz) spectra of kuriloside D (**4**) in C_5D_5N/D_2O (4/1)

Figure S31. HR-ESI-MS and ESI-MS/MS spectra of kuriloside D (**4**)

Figure S32. The ^{13}C NMR (176.03 MHz) spectrum of kuriloside E (**5**) in C_5D_5N/D_2O (4/1)

Figure S33. The ^{1}H NMR (700.00 MHz) spectrum of kuriloside E (**5**) in C_5D_5N/D_2O (4/1)

Figure S34. The COSY (700.00 MHz) spectrum of kuriloside E (**5**) in C_5D_5N/D_2O (4/1)

Figure S35. The HSQC (700.00 MHz) spectrum of kuriloside E (**5**) in C_5D_5N/D_2O (4/1)

Figure S36. The HMBC (700.00 MHz) spectrum of kuriloside E (**5**) in C_5D_5N/D_2O (4/1)

Figure S37. The ROESY (700.00 MHz) spectrum of kuriloside E (**5**) in C_5D_5N/D_2O (4/1)

Figure S38. 1D TOCSY (700.00 MHz) spectra of kuriloside E (**5**) in C_5D_5N/D_2O (4/1)

Figure S39. 1D TOCSY (700.00 MHz) spectra of kuriloside E (**5**) in C_5D_5N/D_2O (4/1)

Figure S40. HR-ESI-MS and ESI-MS/MS spectra of kuriloside E (**5**)

Figure S41. The ^{13}C NMR (176.03 MHz) spectrum of kuriloside F (**6**) in C_5D_5N/D_2O (4/1)

Figure S42. The ^{1}H NMR (700.00 MHz) spectrum of kuriloside F (**6**) in C_5D_5N/D_2O (4/1)

Figure S43. The COSY (700.00 MHz) spectrum of kuriloside F (**6**) in C_5D_5N/D_2O (4/1)

Figure S44. The HSQC (700.00 MHz) spectrum of kuriloside F (**6**) in C_5D_5N/D_2O (4/1)

Figure S45. The ROESY (700.00 MHz) spectrum of kuriloside F (**6**) in C_5D_5N/D_2O (4/1)

Figure S46. The HMBC (700.00 MHz) spectrum of kuriloside F (**6**) in C_5D_5N/D_2O (4/1)

Figure S47. 1D TOCSY (700.00 MHz) spectra of the carbohydrate part of kuriloside F (**6**) in C_5D_5N/D_2O (4/1)

Figure S48. 1D TOCSY (700.00 MHz) spectra of the carbohydrate part of kuriloside F (**6**) in C_5D_5N/D_2O (4/1)

Figure S49. HR-ESI-MS and ESI-MS/MS spectra of kuriloside F (**6**)

Figure S50. The ^{13}C NMR (176.03 MHz) spectrum of kuriloside A (**7**) in C_5D_5N/D_2O (4/1)

Figure S51. The ^{1}H NMR (700.00 MHz) spectrum of kuriloside A (**7**) in C_5D_5N/D_2O (4/1)

Figure S52. The COSY (700.00 MHz) spectrum of kuriloside A (**7**) in C_5D_5N/D_2O (4/1)

Figure S53. The HSQC (700.00 MHz) spectrum of kuriloside A (**7**) in C_5D_5N/D_2O (4/1)

Figure S54. The HMBC (700.00 MHz) spectrum of kuriloside A (**7**) in C_5D_5N/D_2O (4/1)

Figure S55. The ROESY (700.00 MHz) spectrum of kuriloside A (**7**) in C_5D_5N/D_2O (4/1)

Figure S56. 1 D TOCSY (700.00 MHz) spectra of kuriloside A (**7**) in C_5D_5N/D_2O (4/1)

Figure S57. 1 D TOCSY (700.00 MHz) spectra of kuriloside A (**7**) in C_5D_5N/D_2O (4/1)

Figure S58. HR-ESI-MS and ESI-MS/MS spectra of kuriloside A (**7**)

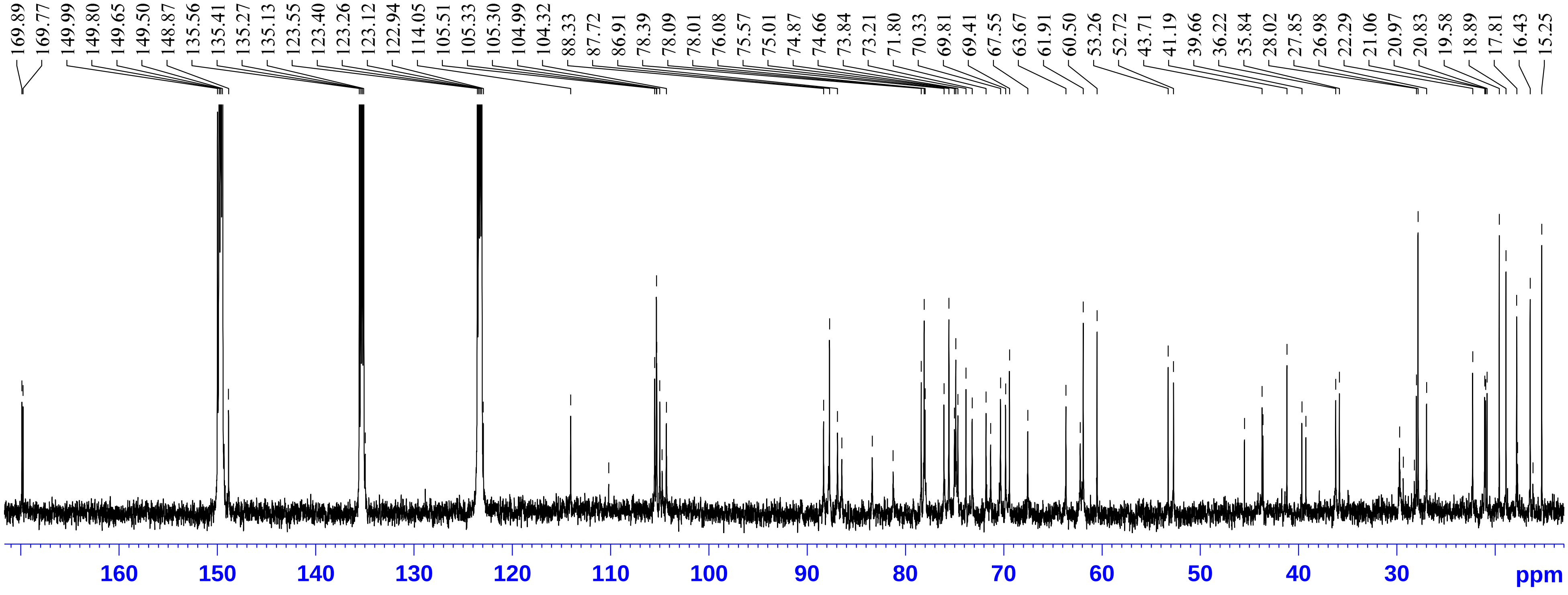

Figure S1. The ^{13}C NMR (176.03 MHz) spectrum of kuriloside A1 (**1**) in C_5D_5N/D_2O (4/1)

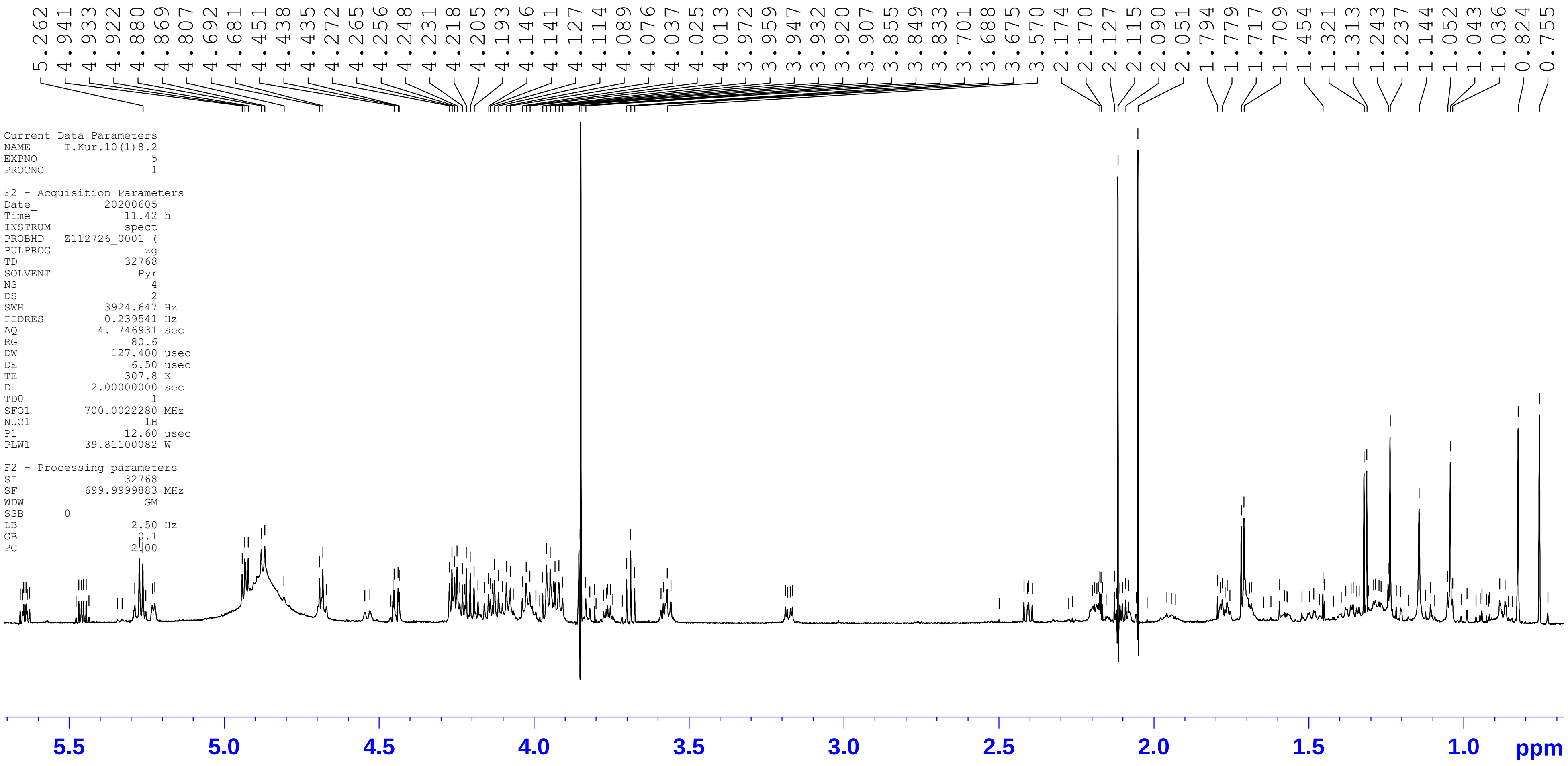

Figure S2. The ^{1}H NMR (700.00 MHz) spectrum of kuriloside A_1 (**1**) in C_5D_5N/D_2O (4/1)

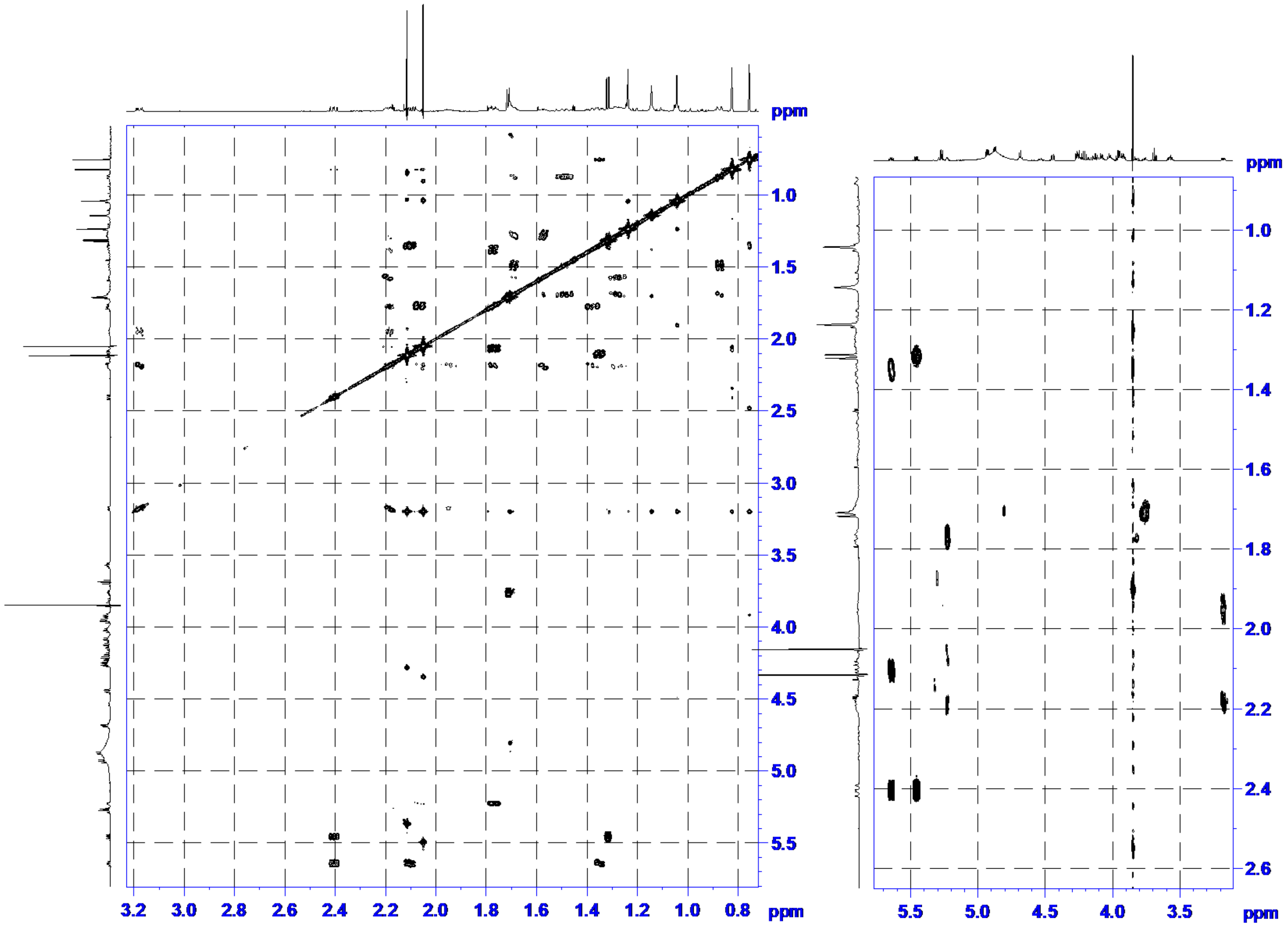

Figure S3. The COSY (700.00 MHz) spectrum of the aglycone part of kuriloside A1 (**1**) in C_5D_5N/D_2O (4/1)

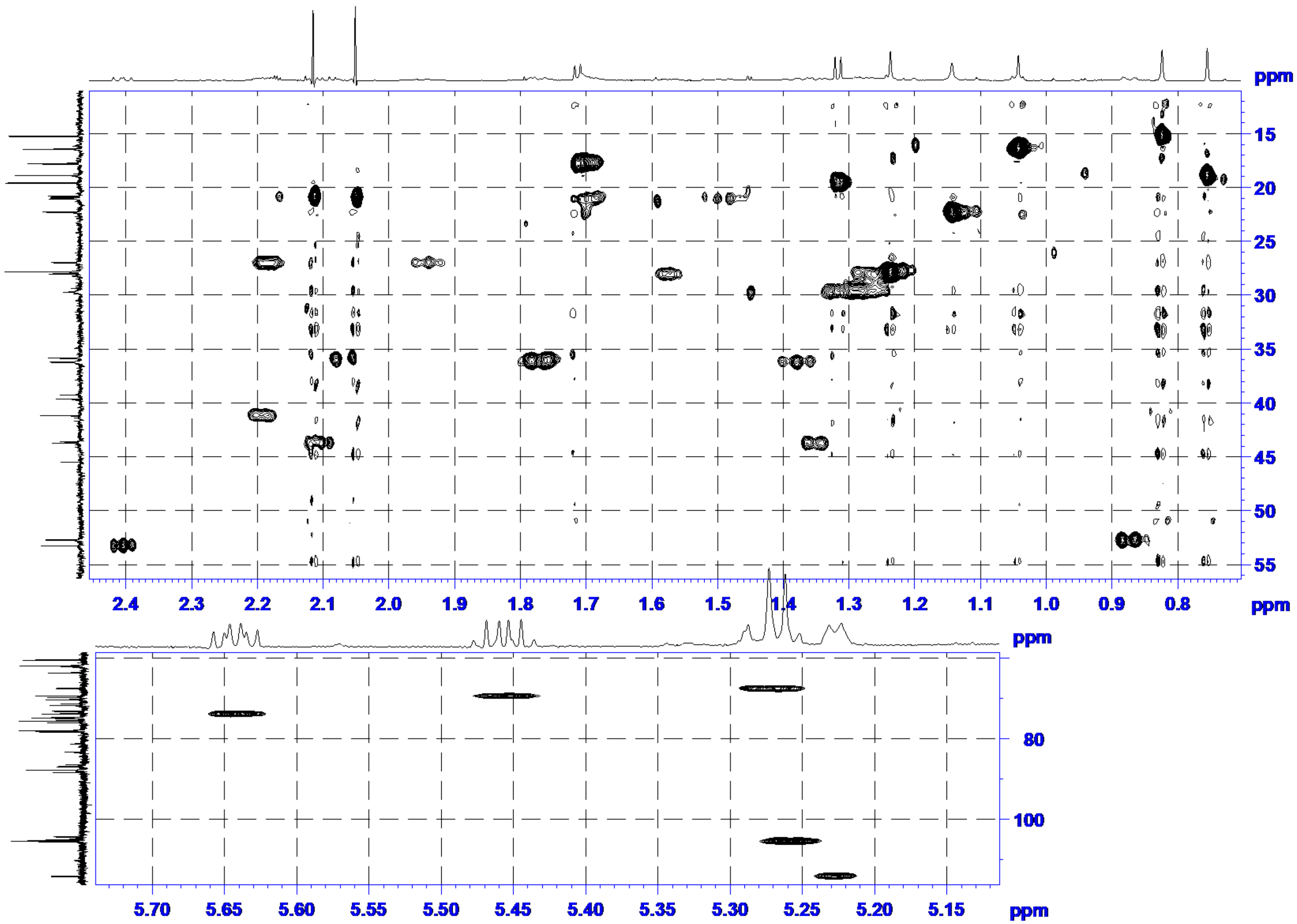

Figure S4. The HSQC (700.00 MHz) spectrum of the aglycone part of kuriloside A_1 (**1**) in C_5D_5N/D_2O (4/1)

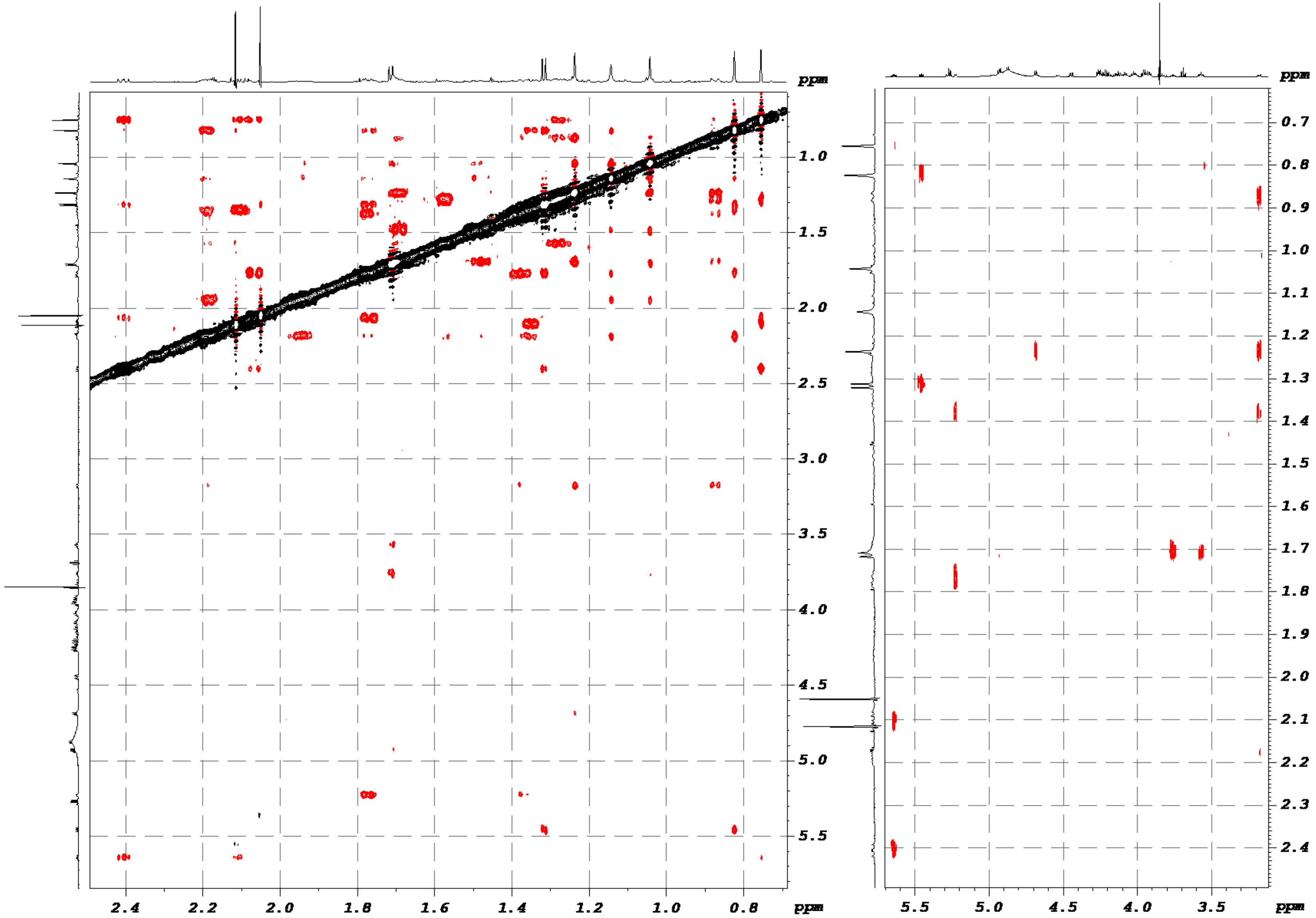

Figure S5. The ROESY (700.00 MHz) spectrum of the aglycone part of kuriloside A_1 (**1**) in C_5D_5N/D_2O (4/1)

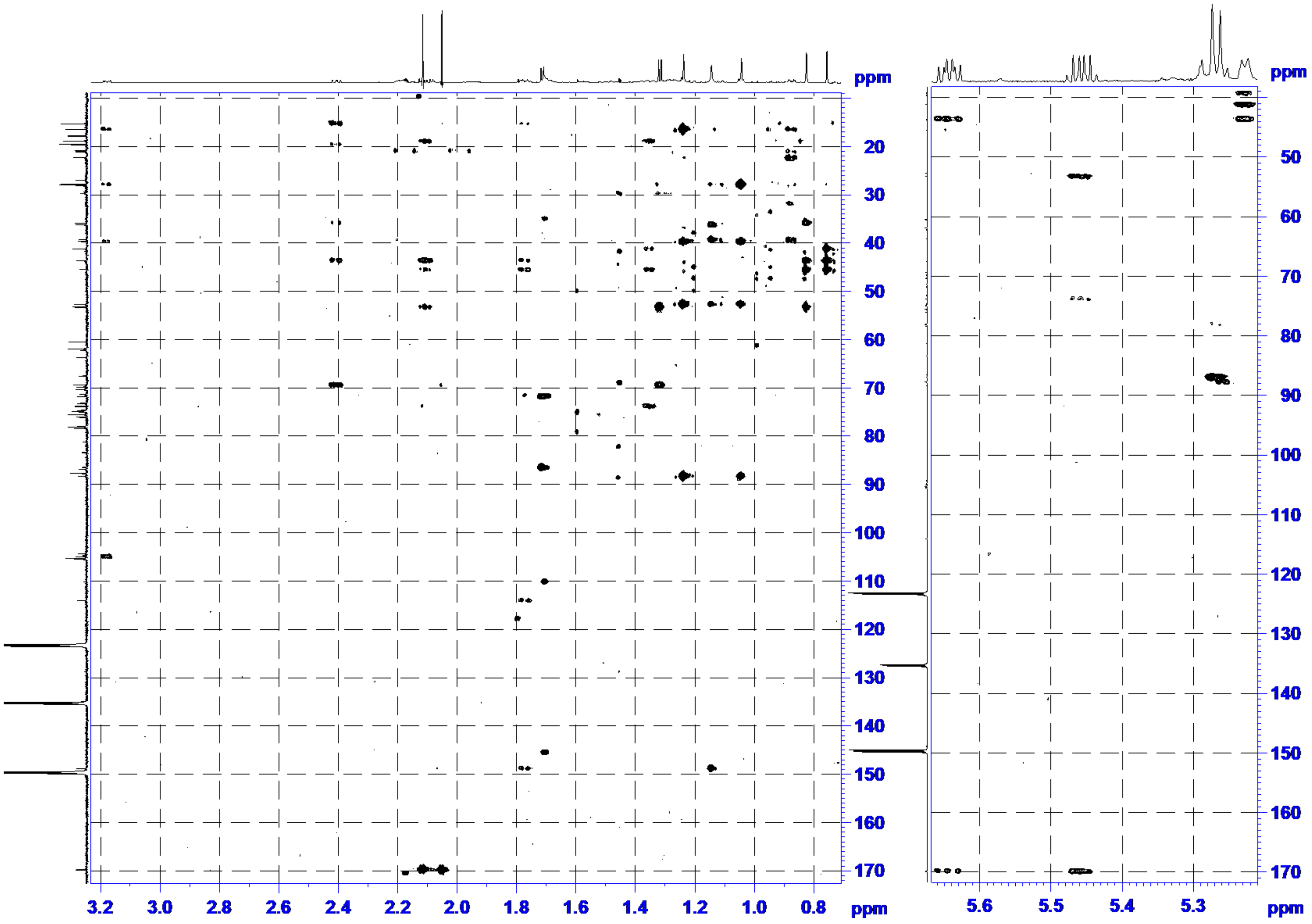

Figure S6. The HMBC (700.00 MHz) spectrum of the aglycone part of kuriloside A1 (**1**) in C_5D_5N/D_2O (4/1)

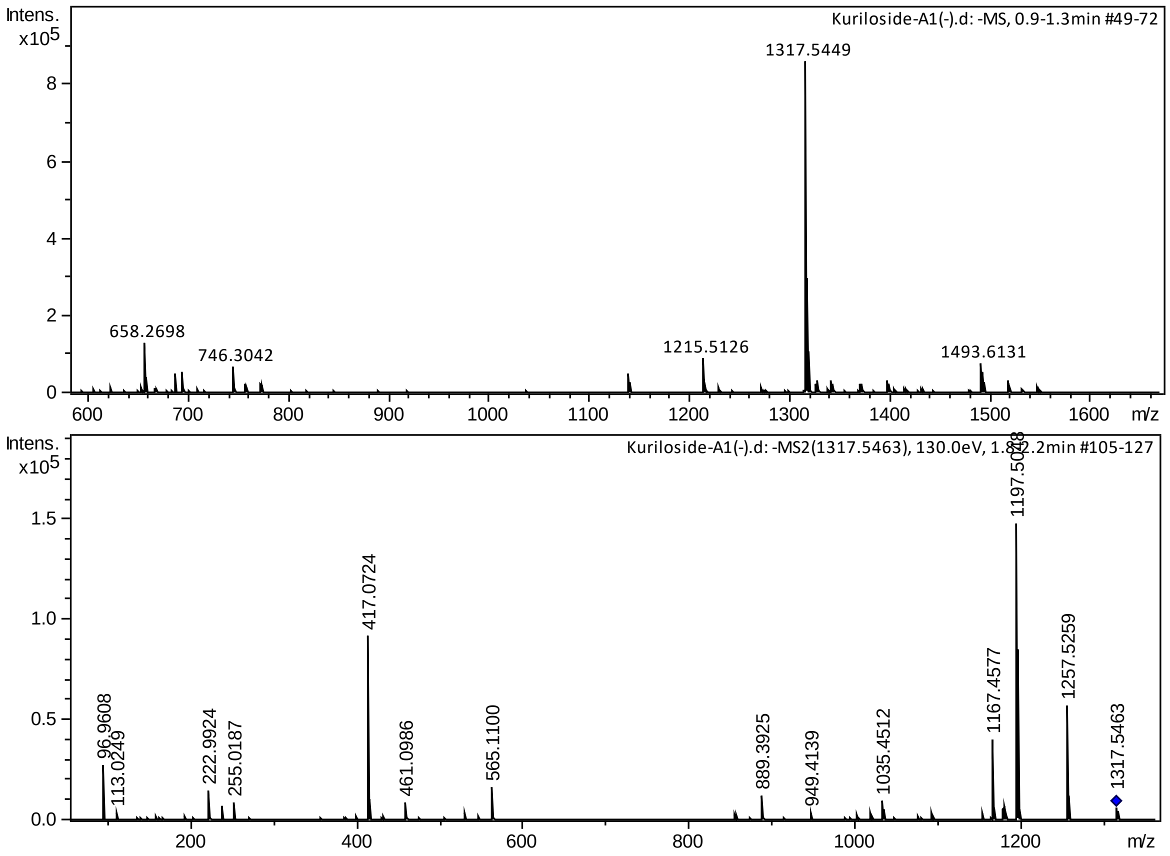

Figure S7. HR-ESI-MS and ESI-MS/MS spectra of kuriloside A1 (**1**)

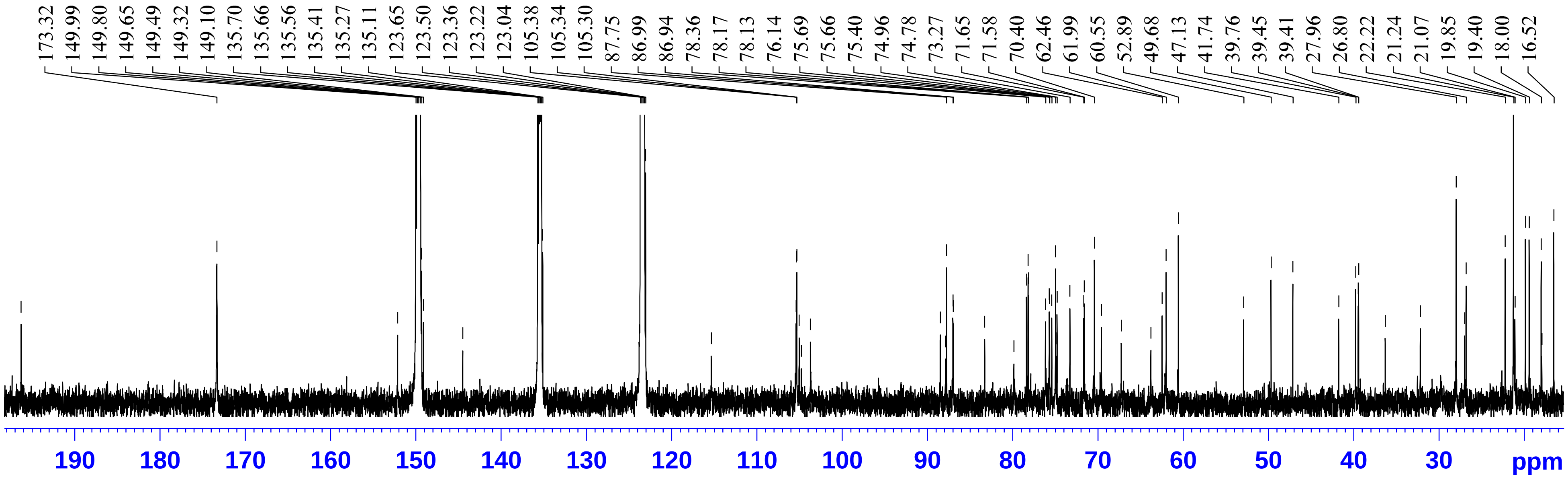

Figure S8. The ^{13}C NMR (176.03 MHz) spectrum of kuriloside A_2 (**2**) in C_5D_5N/D_2O (4/1)

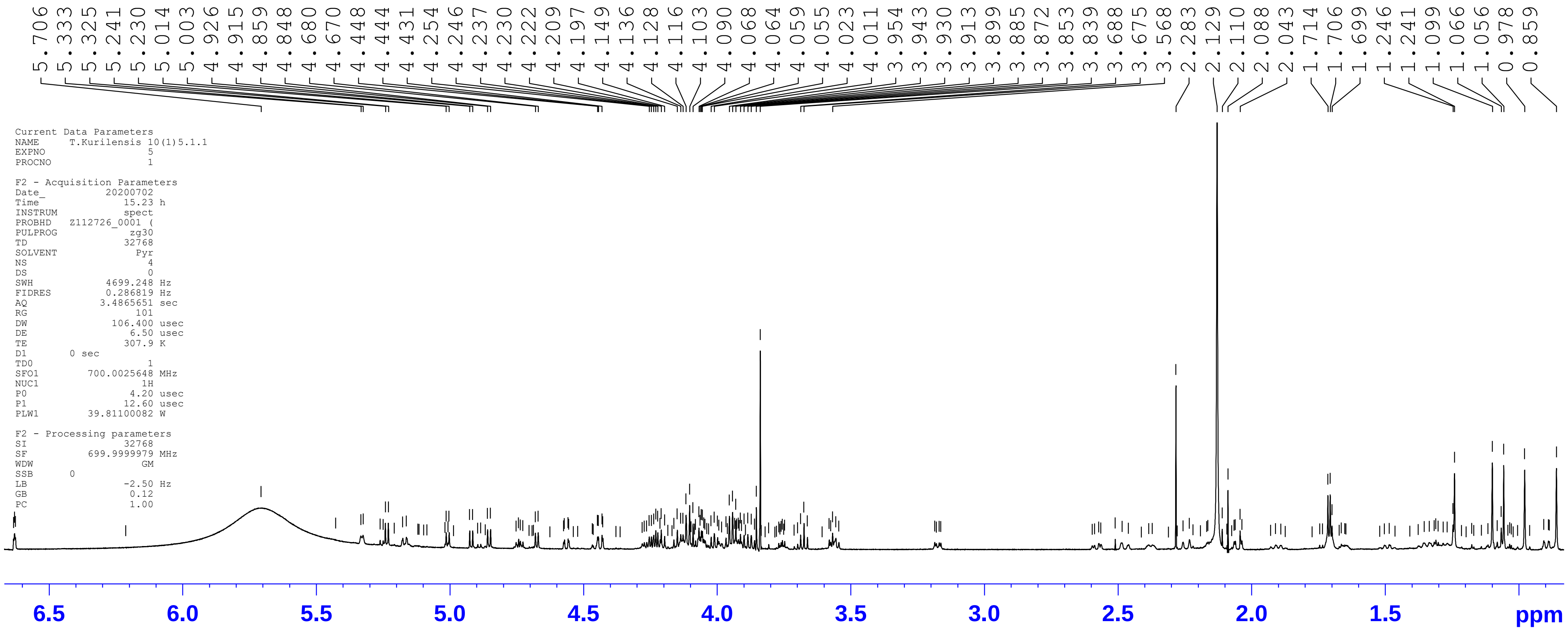

Figure S9. The ^{1}H NMR (700.00 MHz) spectrum of kuriloside A$_2$ (**2**) in C_5D_5N/D_2O (4/1)

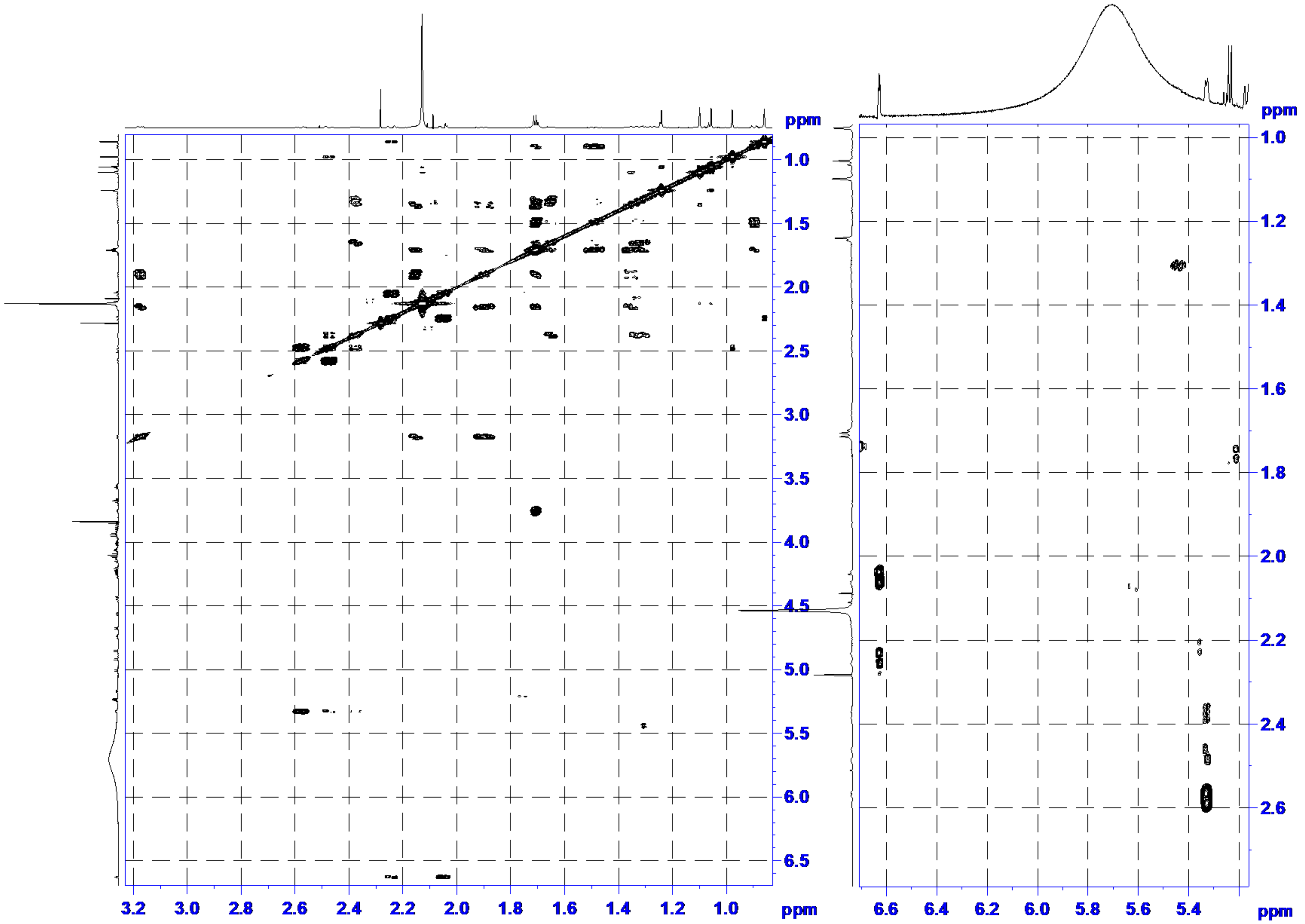

Figure S10. The COSY (700.00 MHz) spectrum of the aglycone part of kuriloside A_2 (**2**) in C_5D_5N/D_2O (4/1)

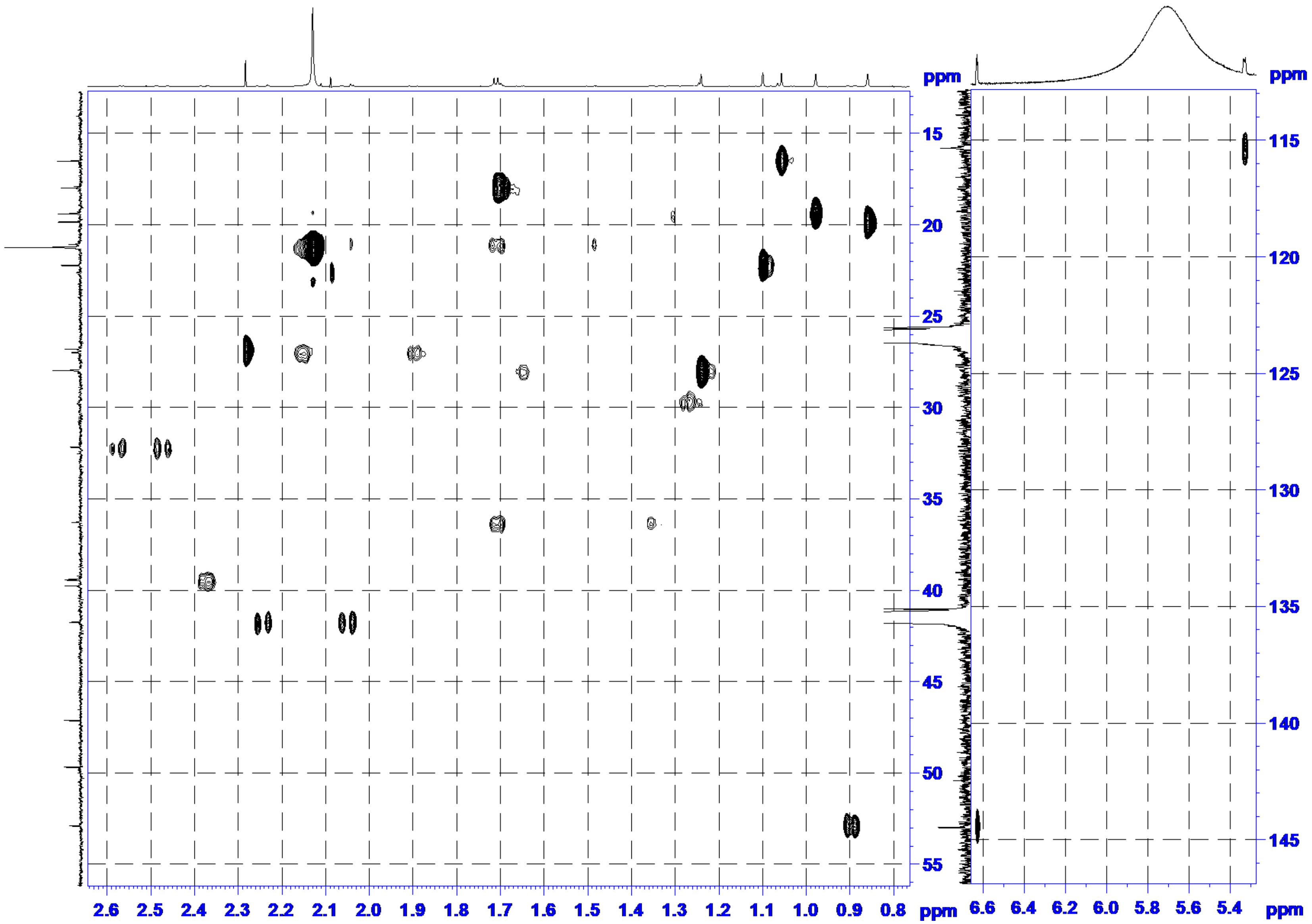

Figure S11. The HSQC (700.00 MHz) spectrum of the aglycone part of kuriloside A_2 (**2**) in C_5D_5N/D_2O (4/1)

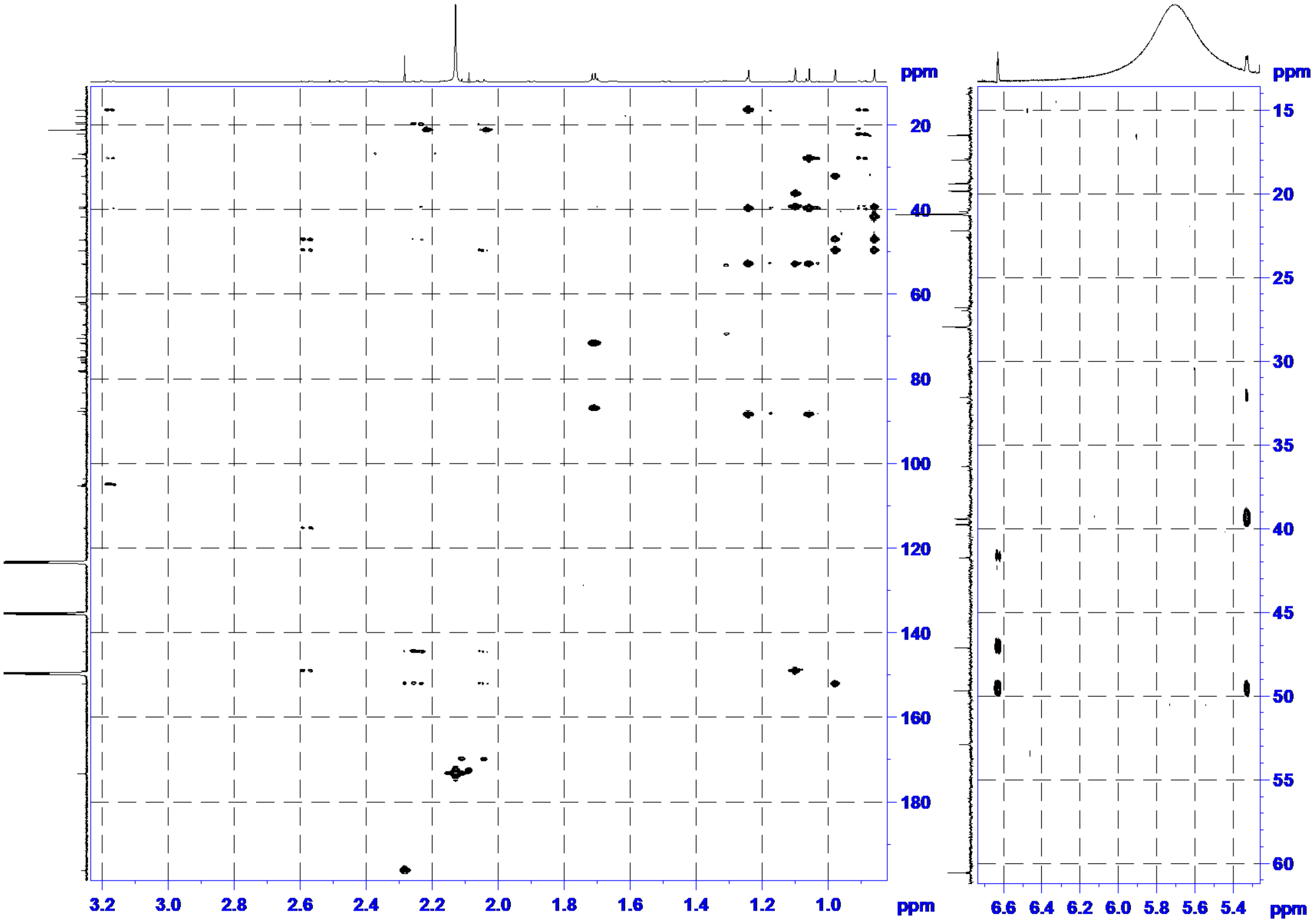

Figure S12. The HMBC (700.00 MHz) spectrum of the aglycone part of kuriloside A2 (**2**) in C_5D_5N/D_2O (4/1)

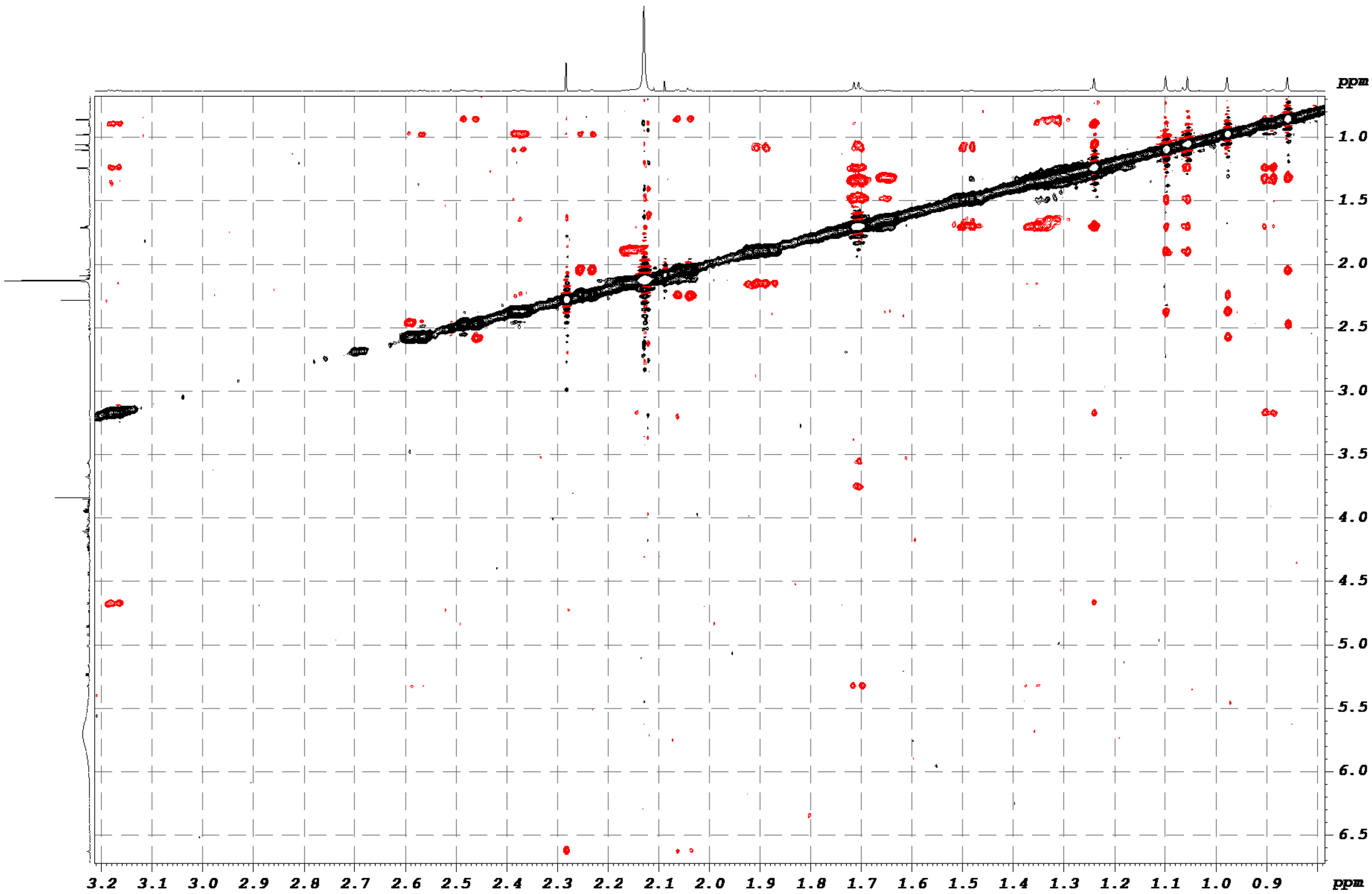

Figure S13. The ROESY (700.00 MHz) spectrum of the aglycone part of kuriloside A2 (**2**) in C_5D_5N/D_2O (4/1)

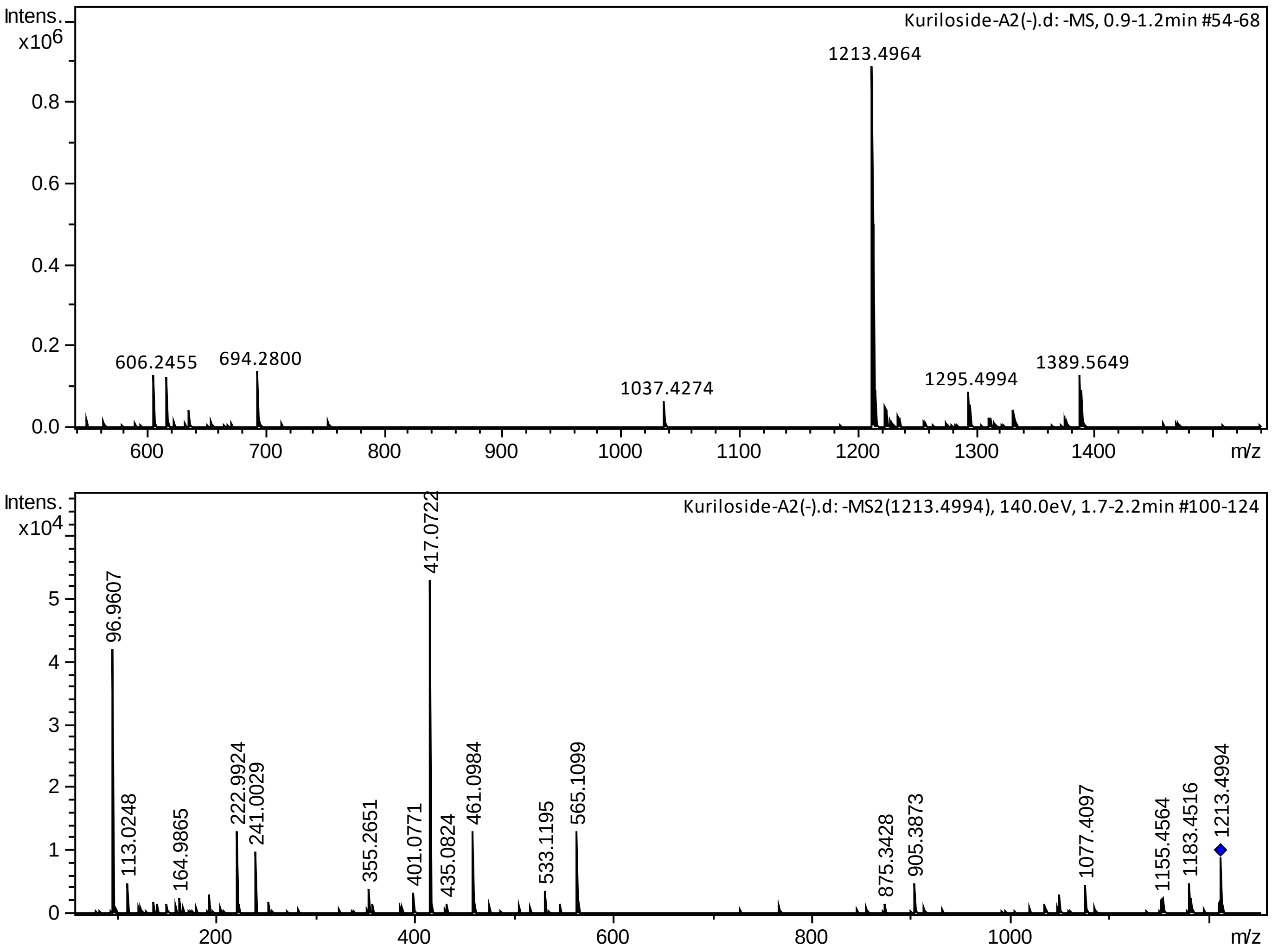

Figure S14. HR-ESI-MS and ESI-MS/MS spectra of kuriloside A_2 (**2**)

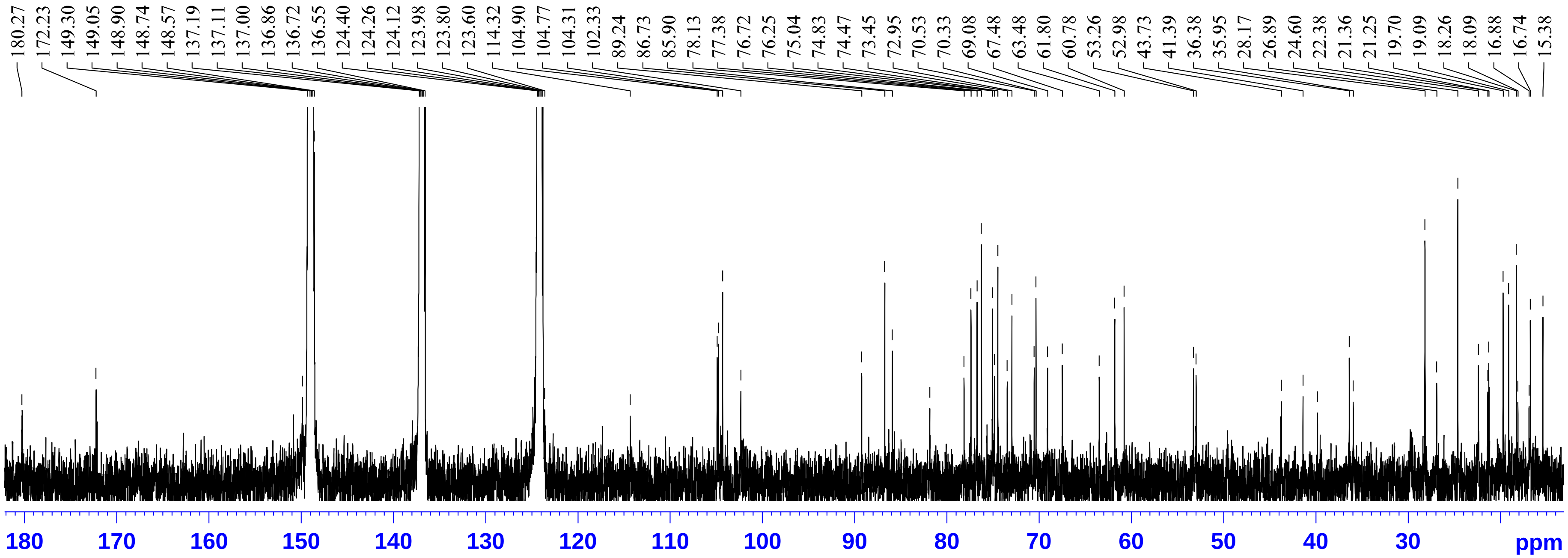

Figure S15. The ^{13}C NMR (176.03 MHz) spectrum of kuriloside C_1 (**3**) in C_5D_5N/D_2O (4/1)

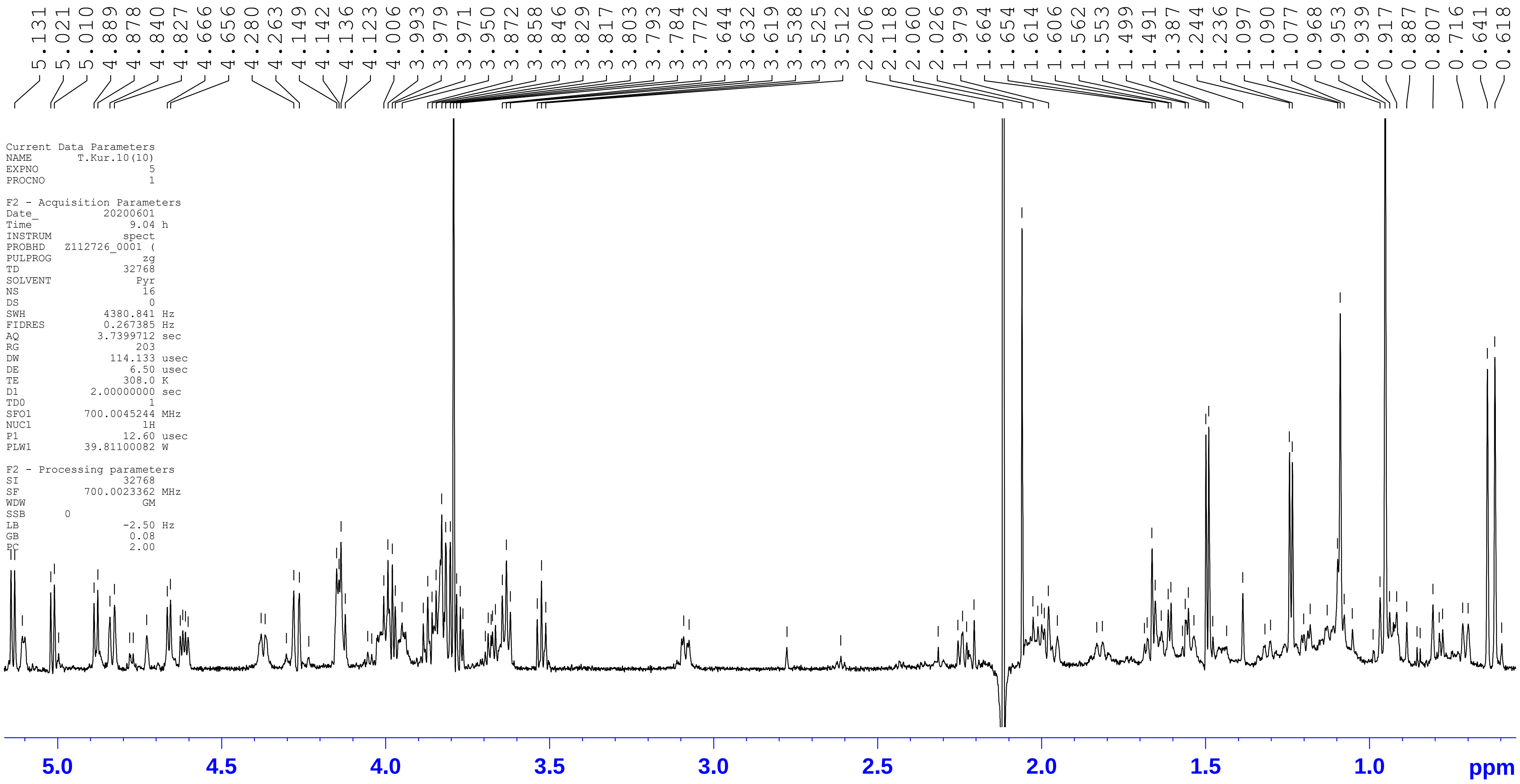

Figure S16. The ^{1}H NMR (700.00 MHz) spectrum of kuriloside C_1 (**3**) in C_5D_5N/D_2O (4/1)

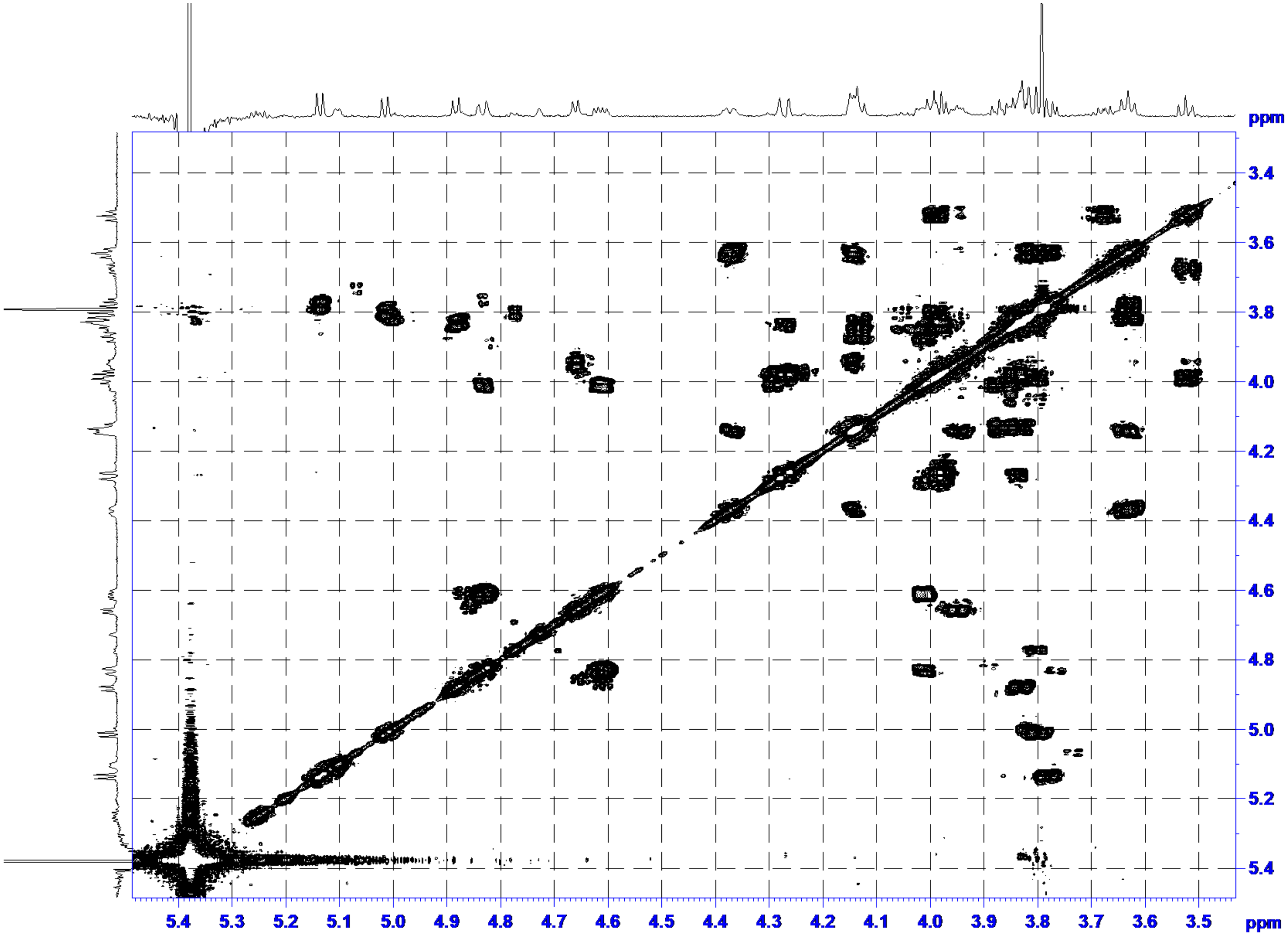

Figure S17. The COSY (700.00 MHz) spectrum of the carbohydrate part of kuriloside C1 (**3**) in C_5D_5N/D_2O (4/1)

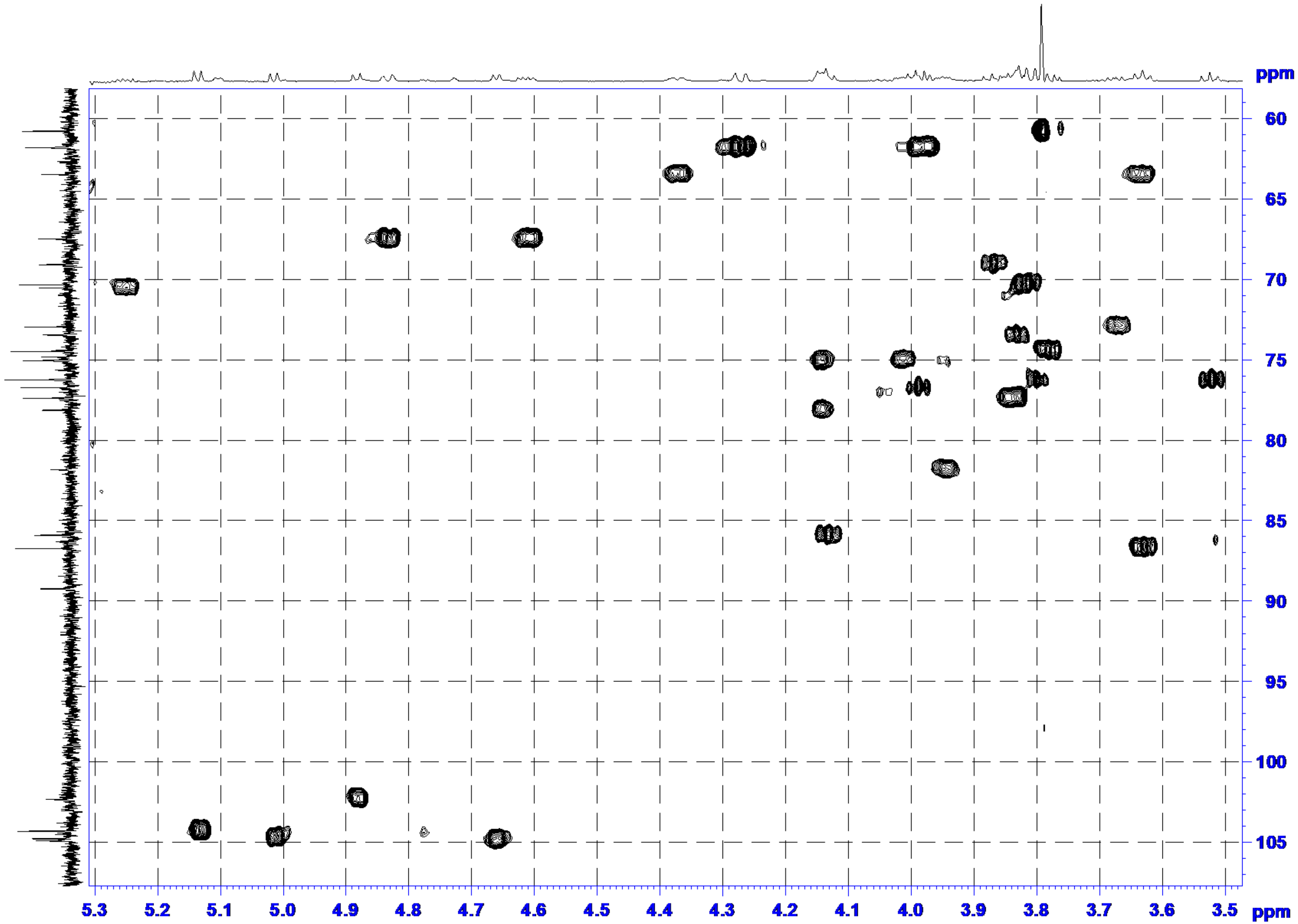

Figure S18. The HSQC (700.00 MHz) spectrum of the carbohydrate part of kuriloside C_1 (**3**) in C_5D_5N/D_2O (4/1)

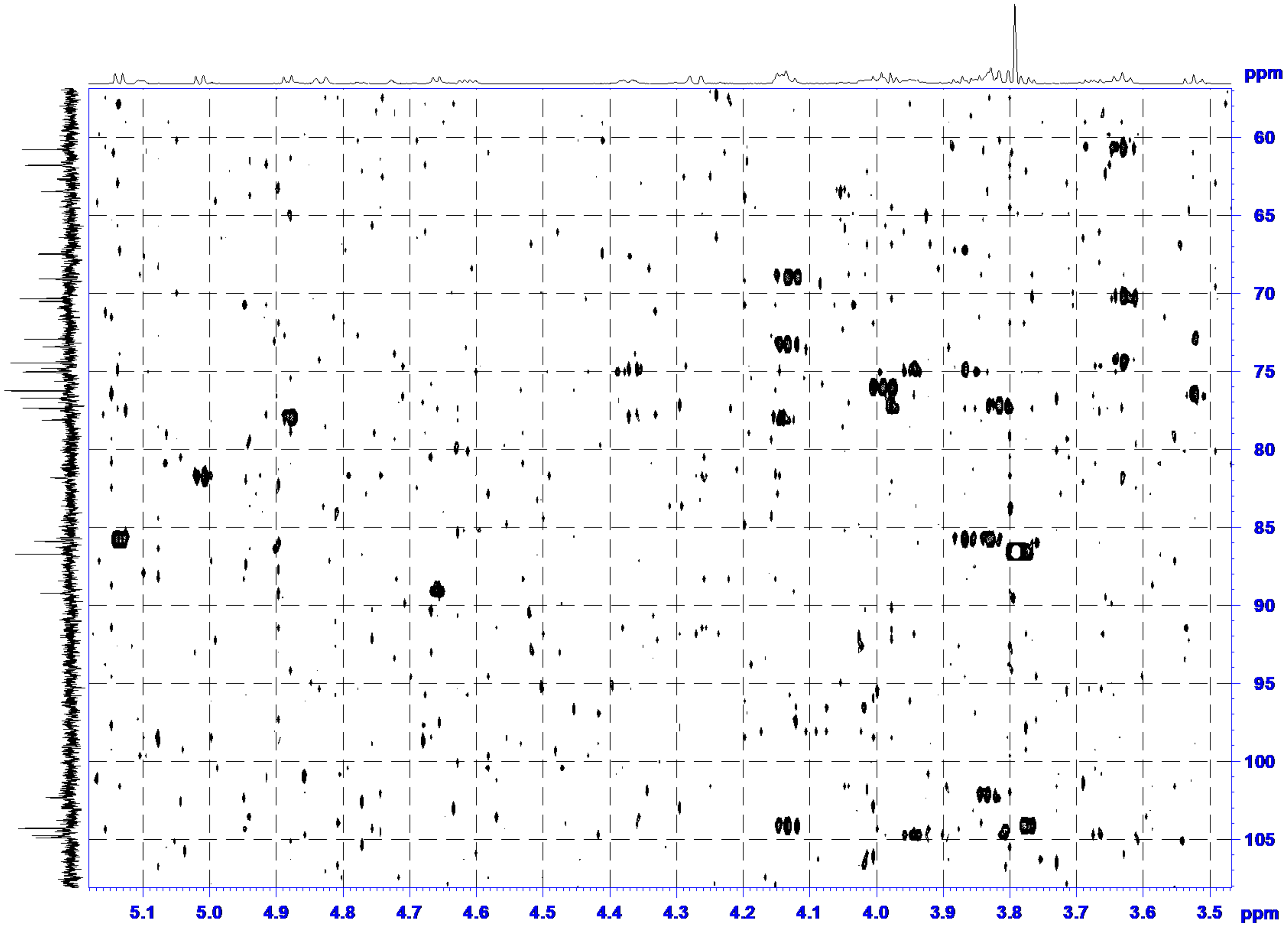

Figure S19. The HMBC (700.00 MHz) spectrum of the carbohydrate part of kuriloside C_1 (**3**) in C_5D_5N/D_2O (4/1)

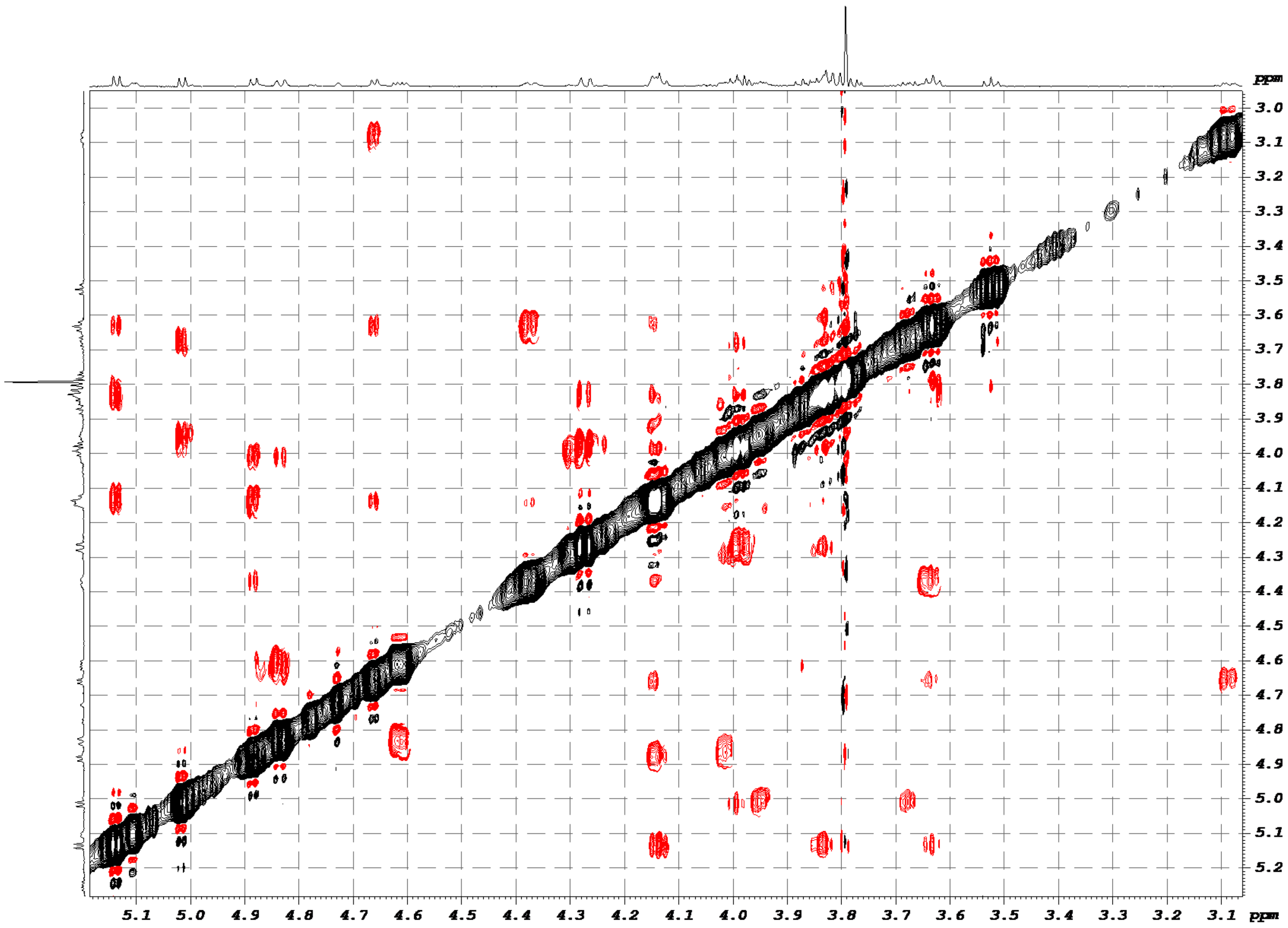

Figure S20. The ROESY (700.00 MHz) spectrum of the carbohydrate part of kuriloside C_1 (**3**) in C_5D_5N/D_2O (4/1)

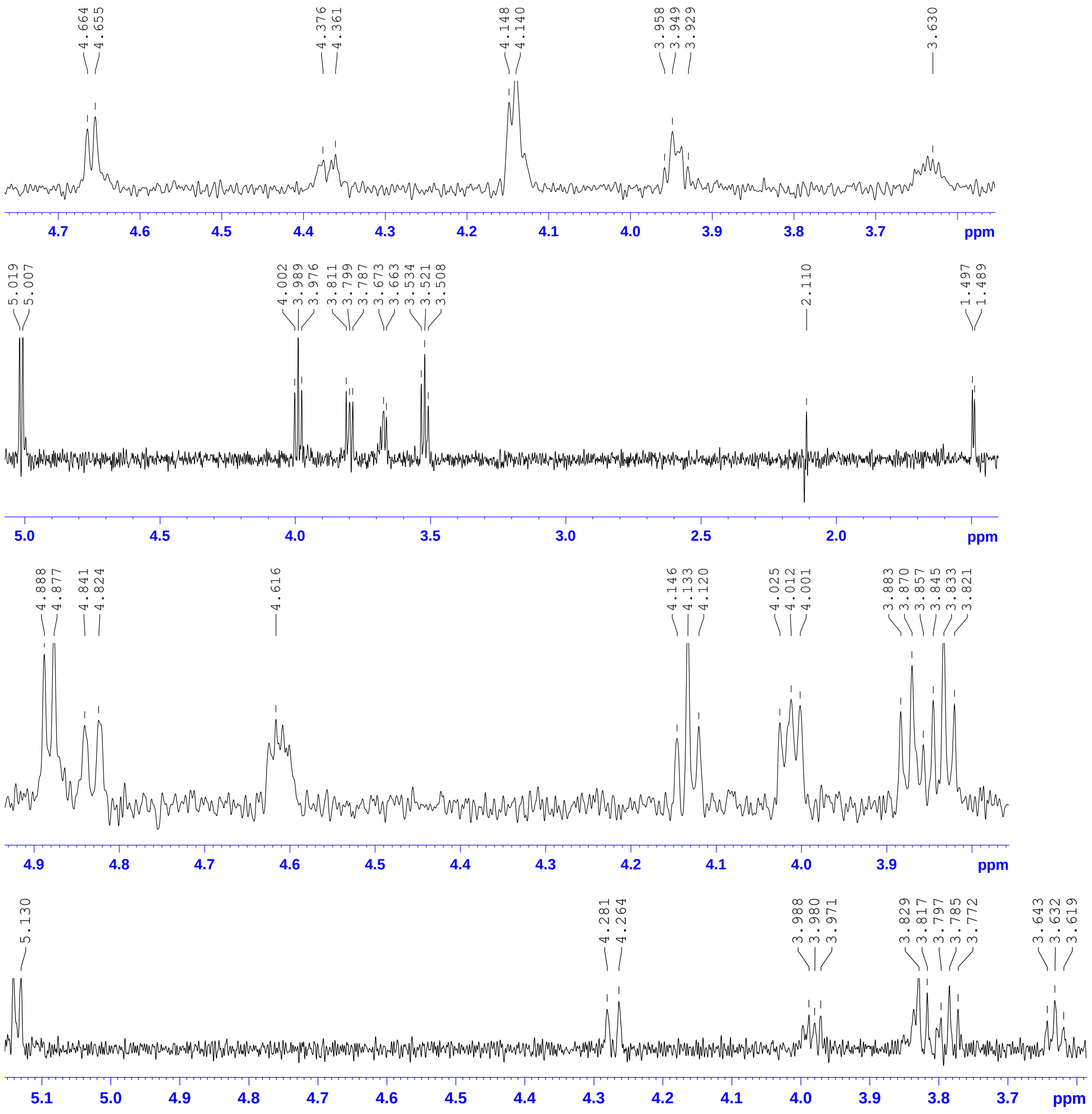

Figure S21. 1 D TOCSY (700.00 MHz) spectra of the carbohydrate part of kuriloside C_1 (**3**) in C_5D_5N/D_2O (4/1)

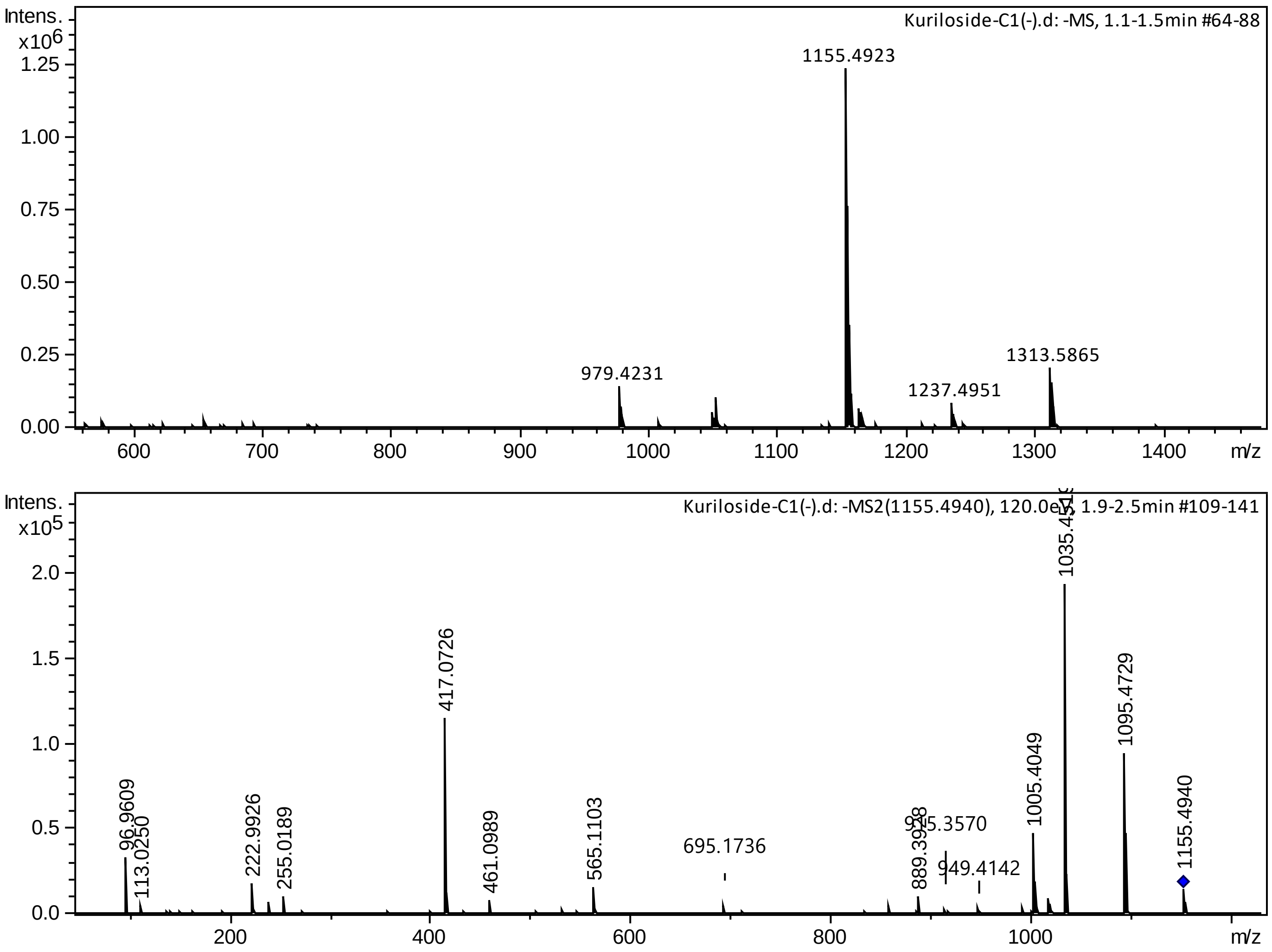

Figure S22. HR-ESI-MS and ESI-MS/MS spectra of kuriloside C_1 (**3**)

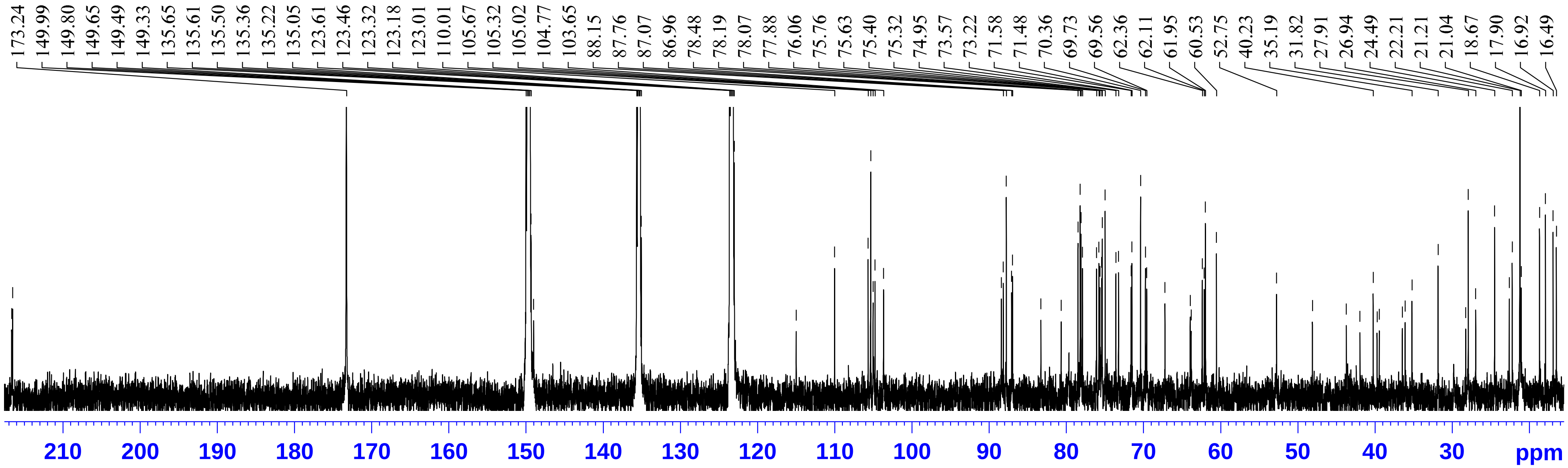

Figure S23. The ^{13}C NMR (176.03 MHz) spectrum of kuriloside D (**4**) in C_5D_5N/D_2O (4/1)

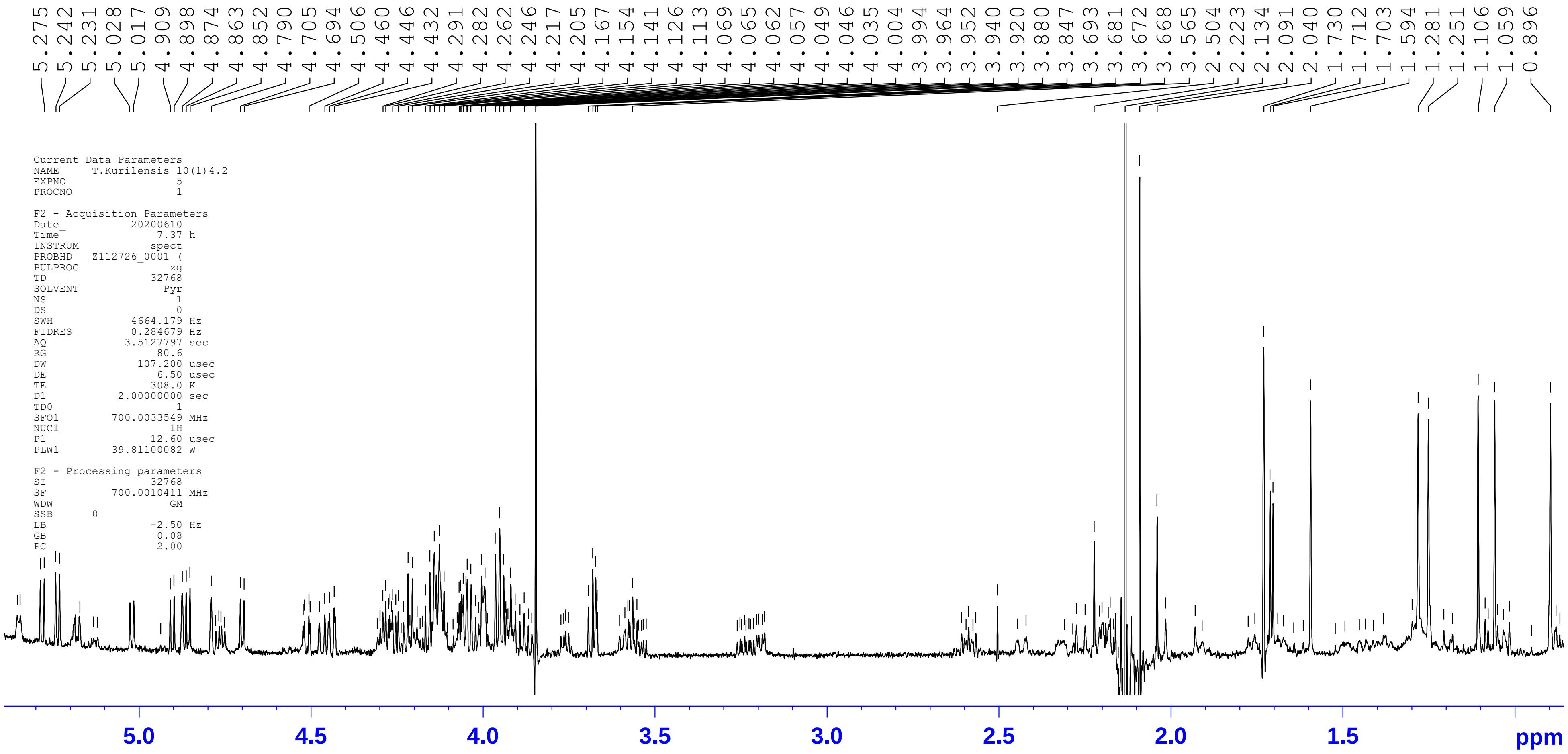

Figure S24. The ^{1}H NMR (700.00 MHz) spectrum of kuriloside D (**4**) in C_5D_5N/D_2O (4/1)

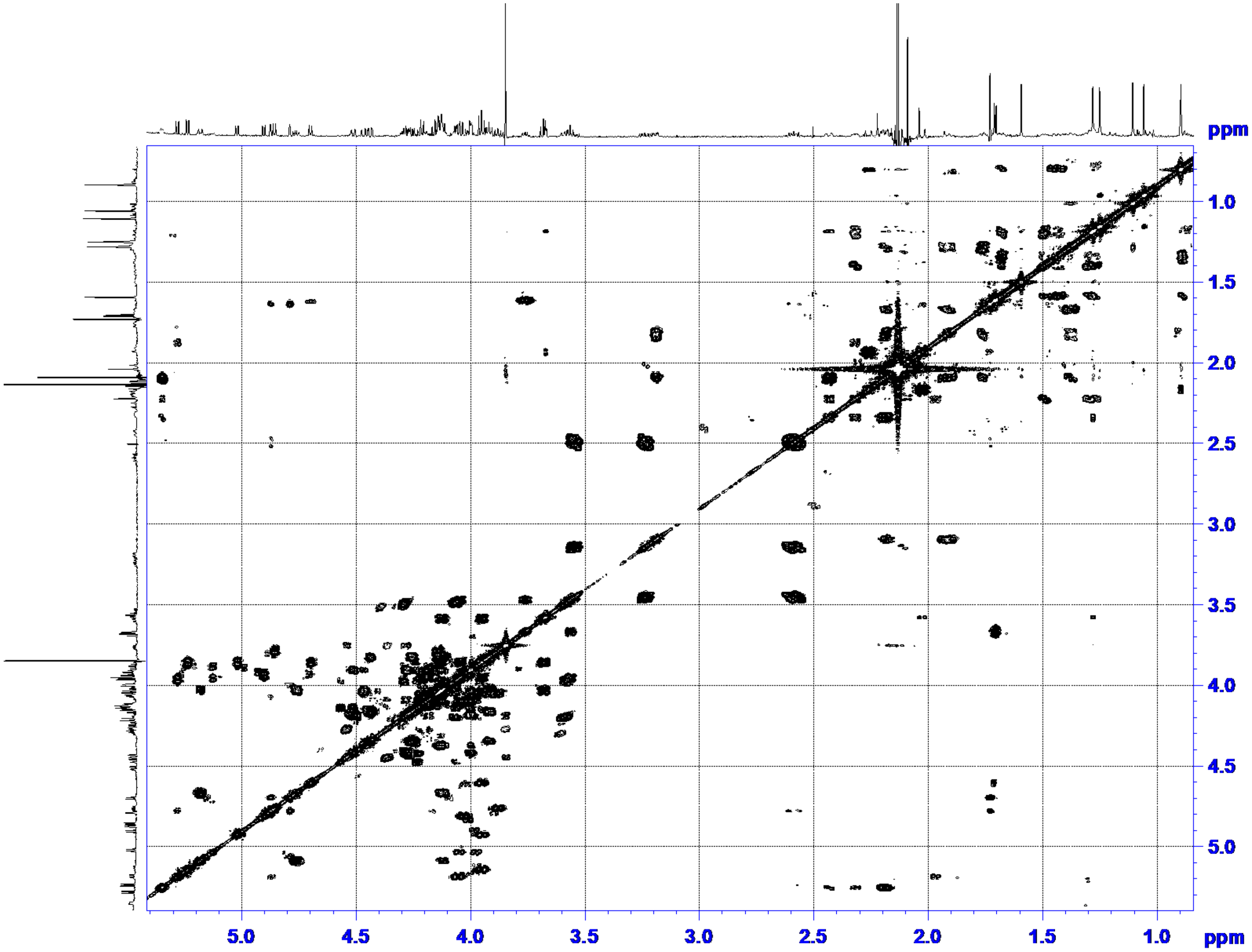

Figure S25. The COSY (700.00 MHz) spectrum of kuriloside D (**4**) in C_5D_5N/D_2O (4/1)

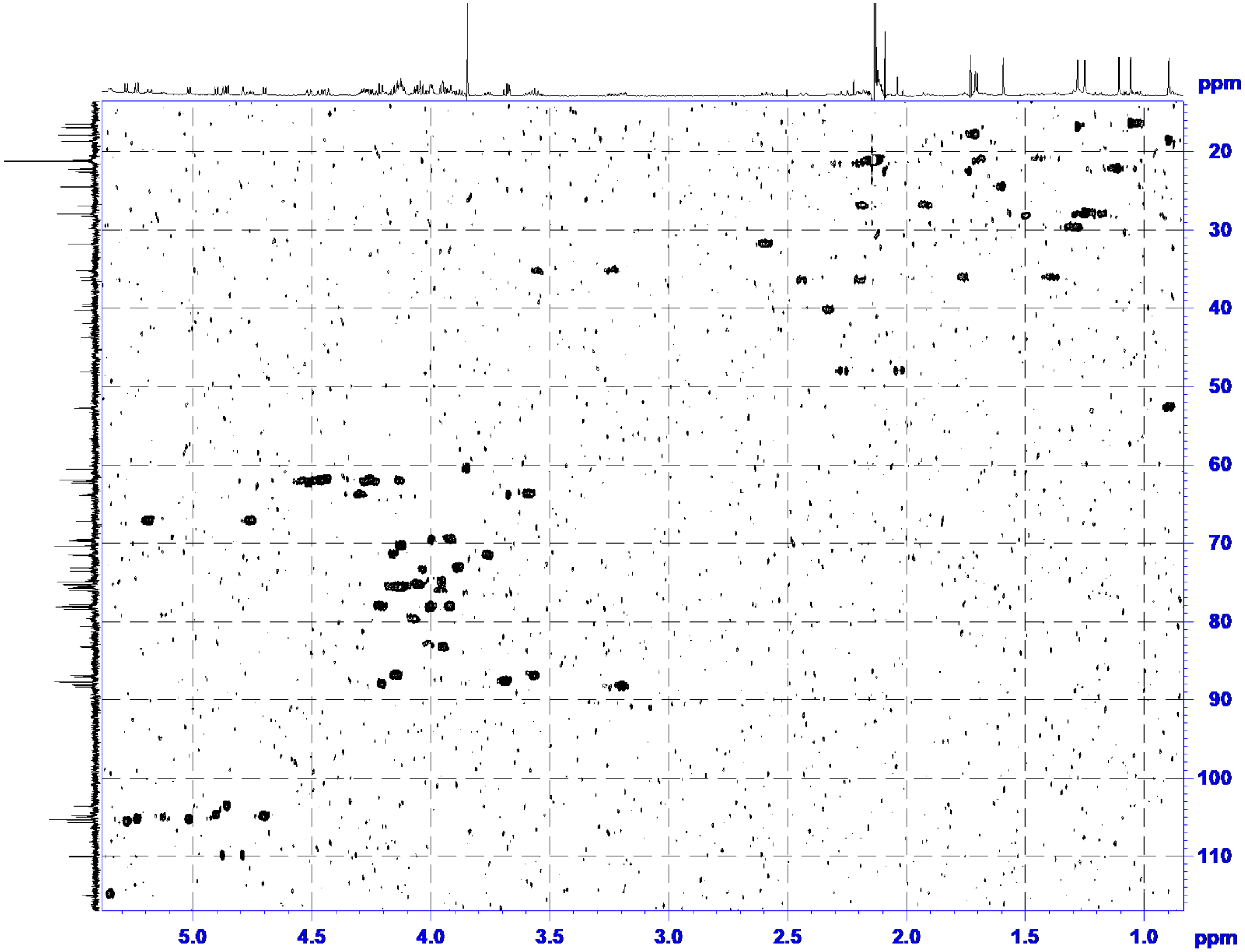

Figure S26. The HSQC (700.00 MHz) spectrum of kuriloside D (**4**) in C_5D_5N/D_2O (4/1)

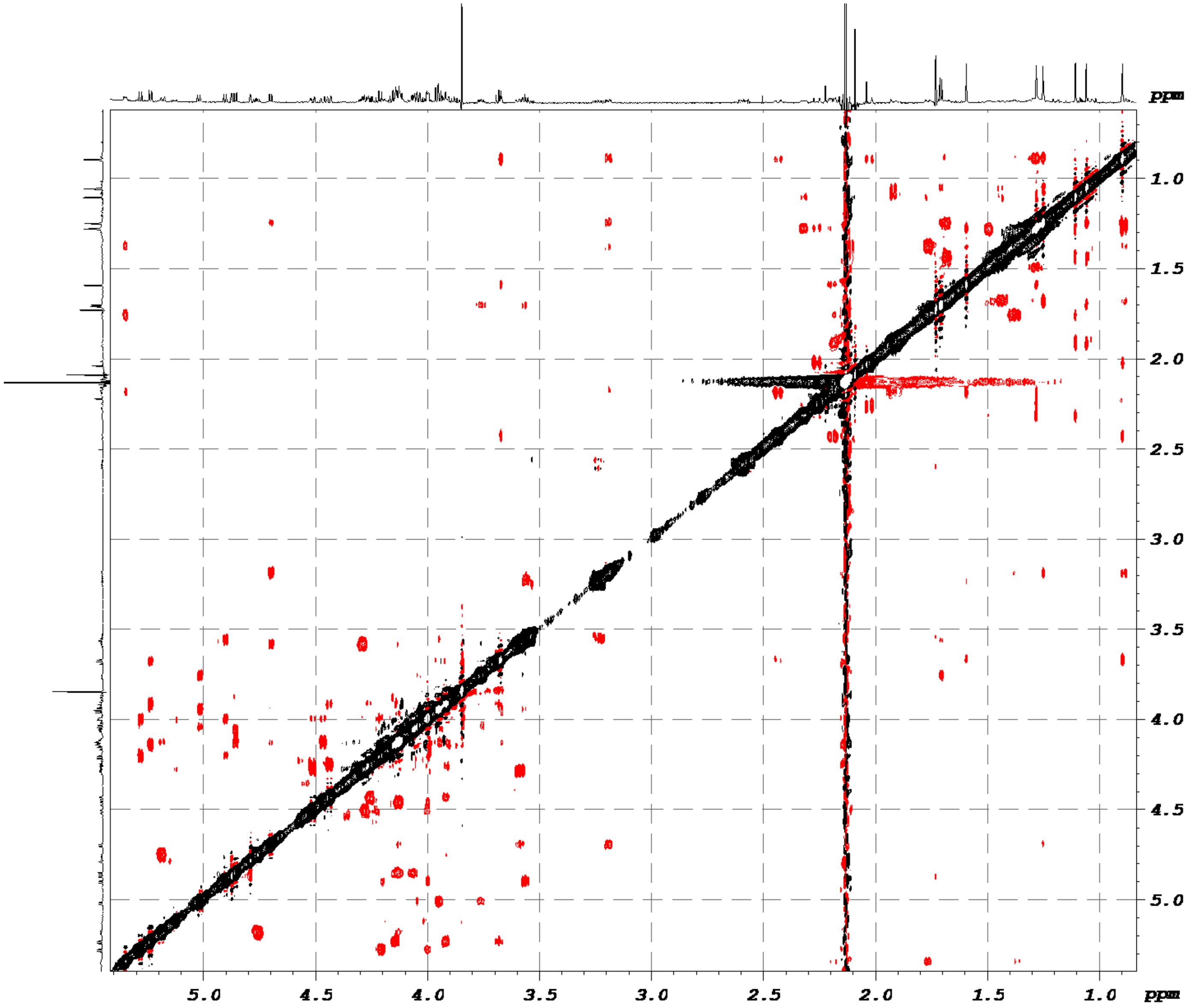

Figure S27. The ROESY (700.00 MHz) spectrum of kuriloside D (**4**) in C_5D_5N/D_2O (4/1)

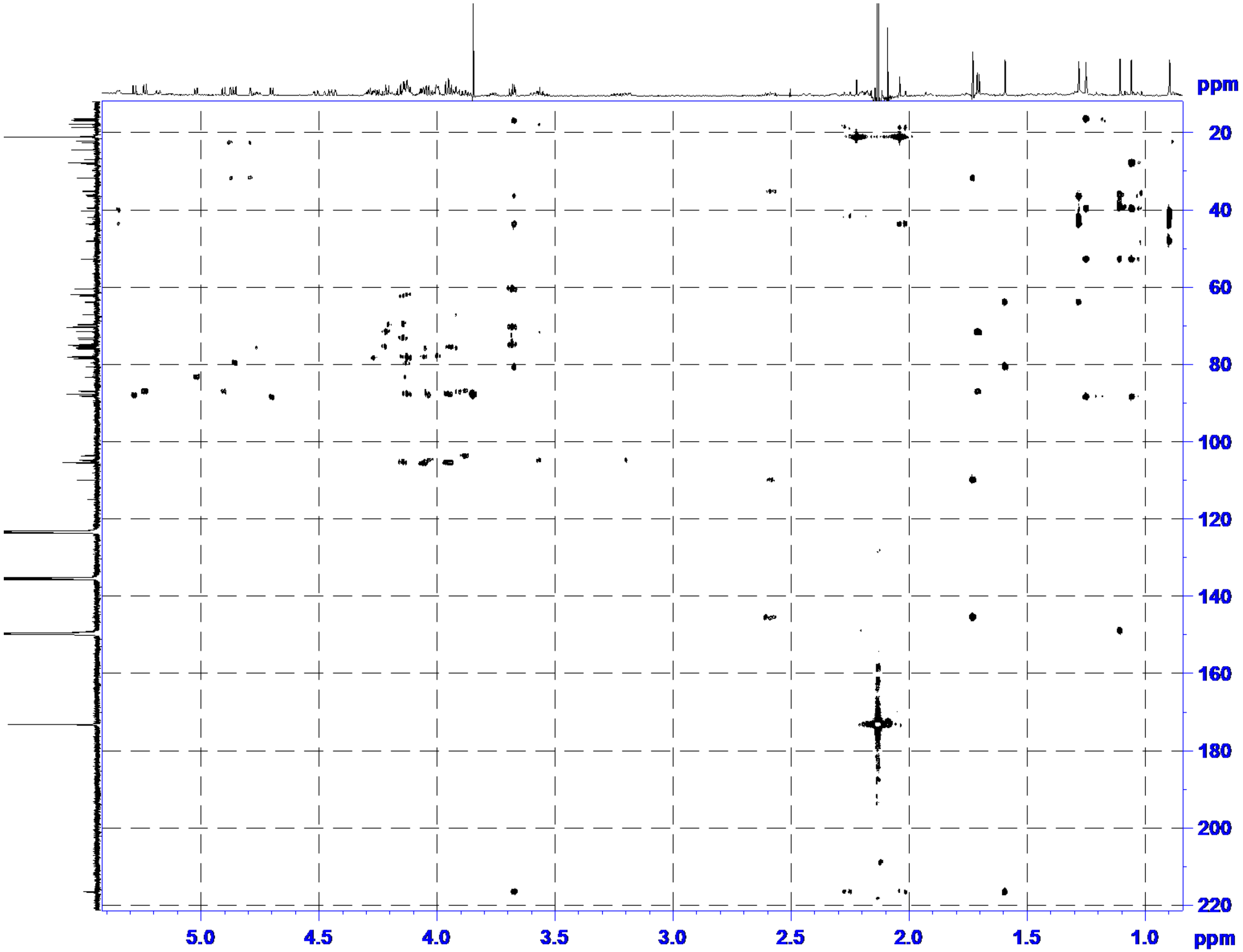

Figure S28. The HMBC (700.00 MHz) spectrum of kuriloside D (**4**) in C_5D_5N/D_2O (4/1)

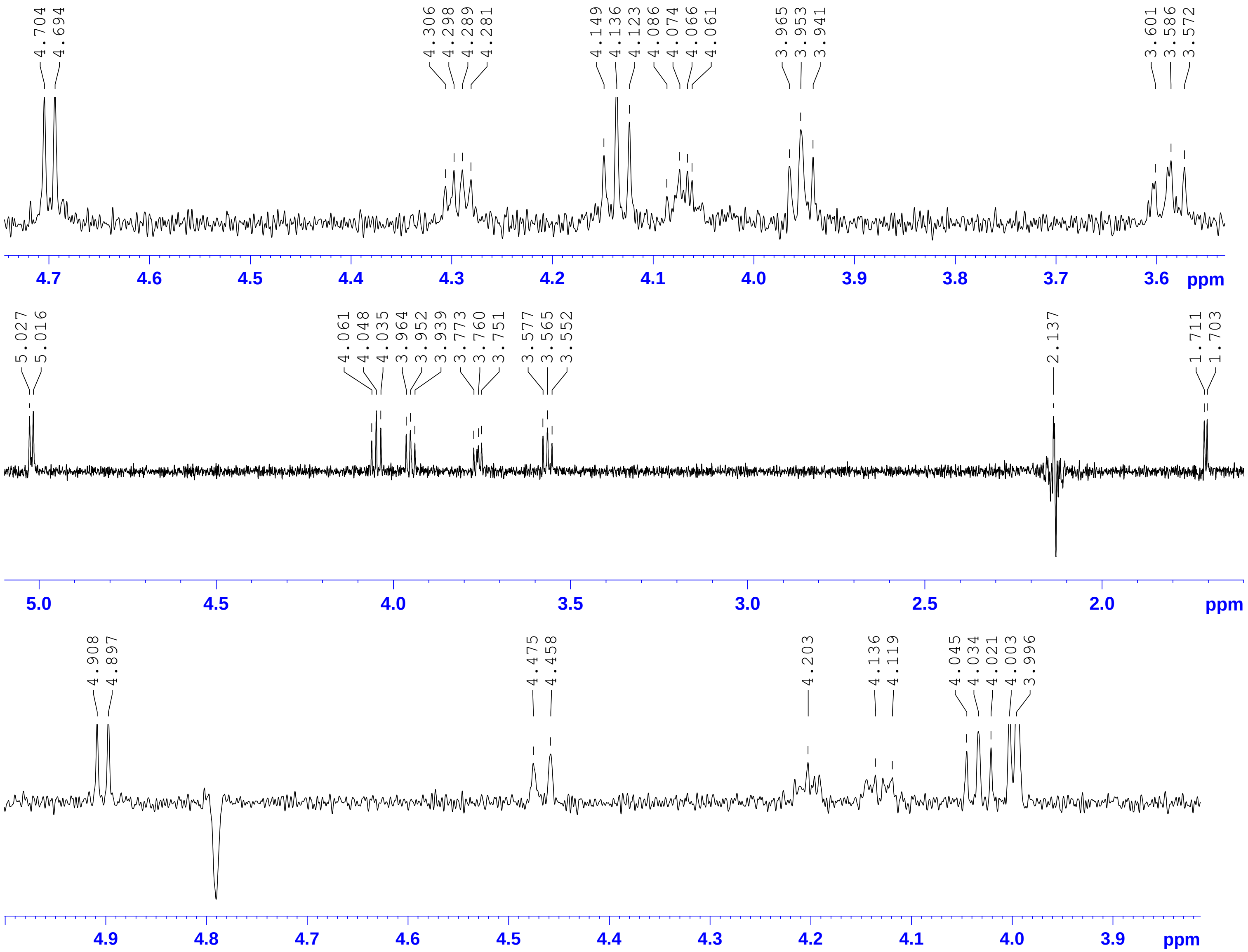

Figure S29. 1 D TOCSY (700.00 MHz) spectra of kuriloside D (**4**) in C_5D_5N/D_2O (4/1)

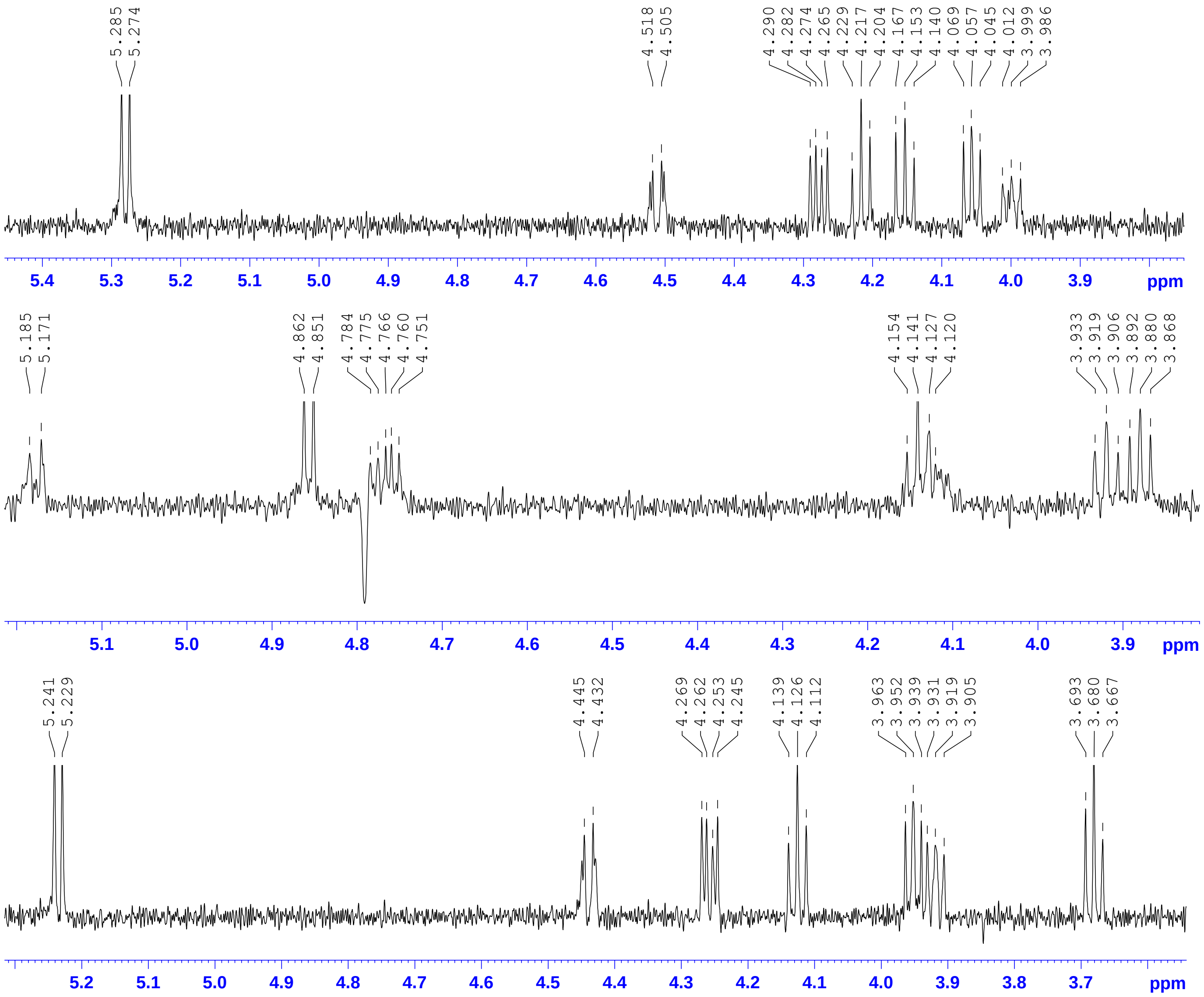

Figure S30. 1 D TOCSY (700.00 MHz) spectra of kuriloside D (**4**) in C_5D_5N/D_2O (4/1)

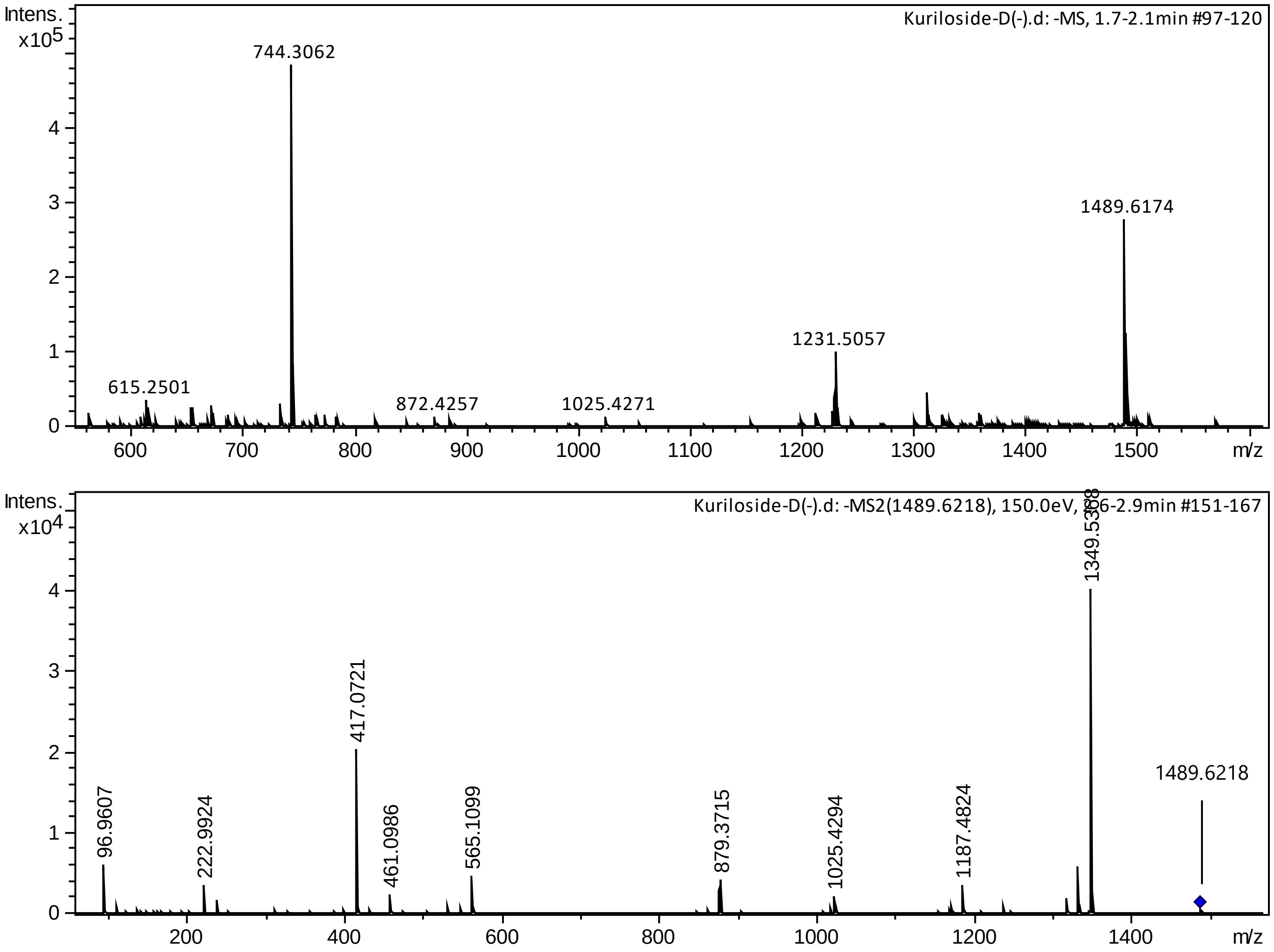

Figure S31. HR-ESI-MS and ESI-MS/MS spectra of kuriloside D (**4**)

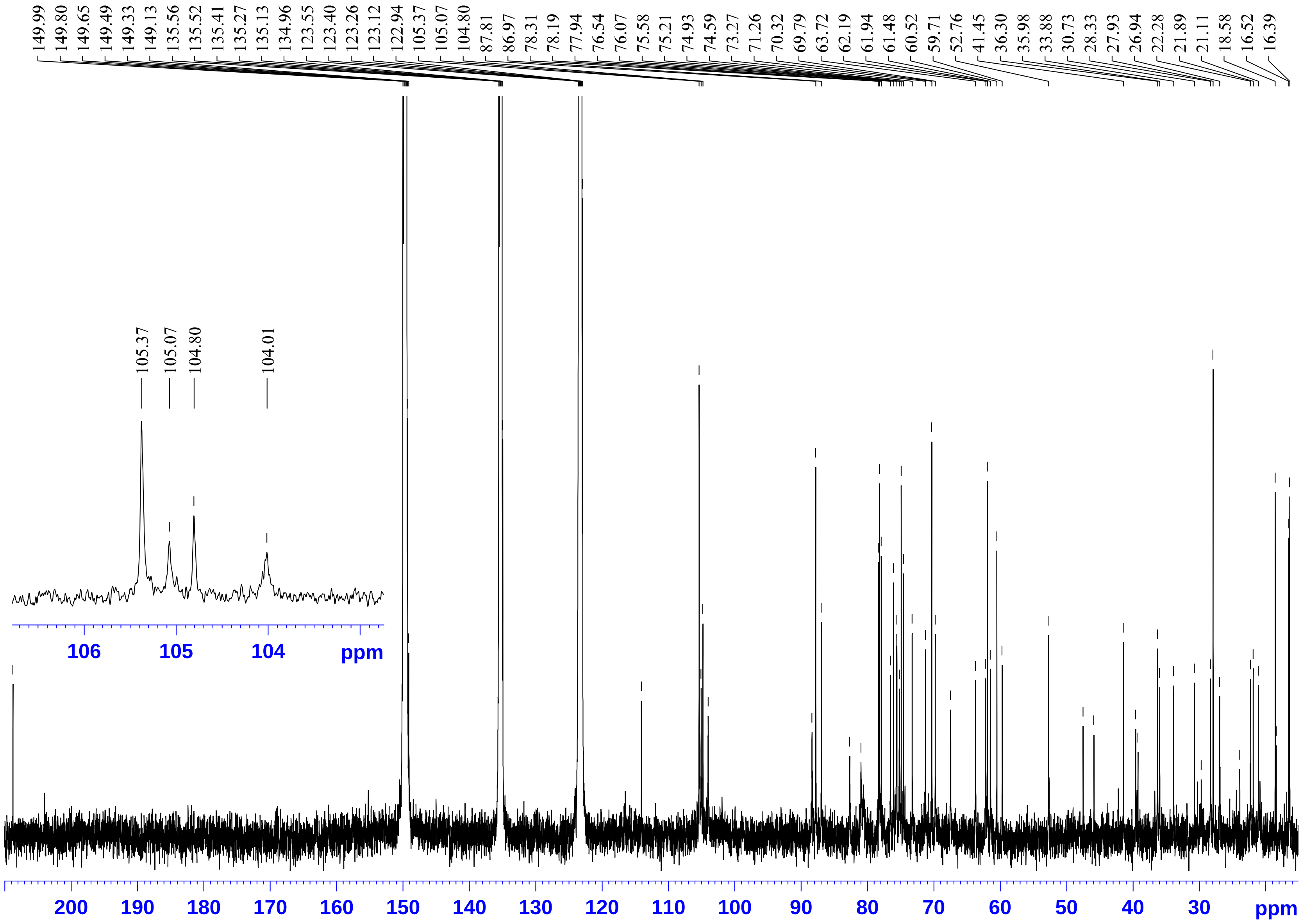

Figure S32. The ^{13}C NMR (176.03 MHz) spectrum of kuriloside E (**5**) in C_5D_5N/D_2O (4/1)

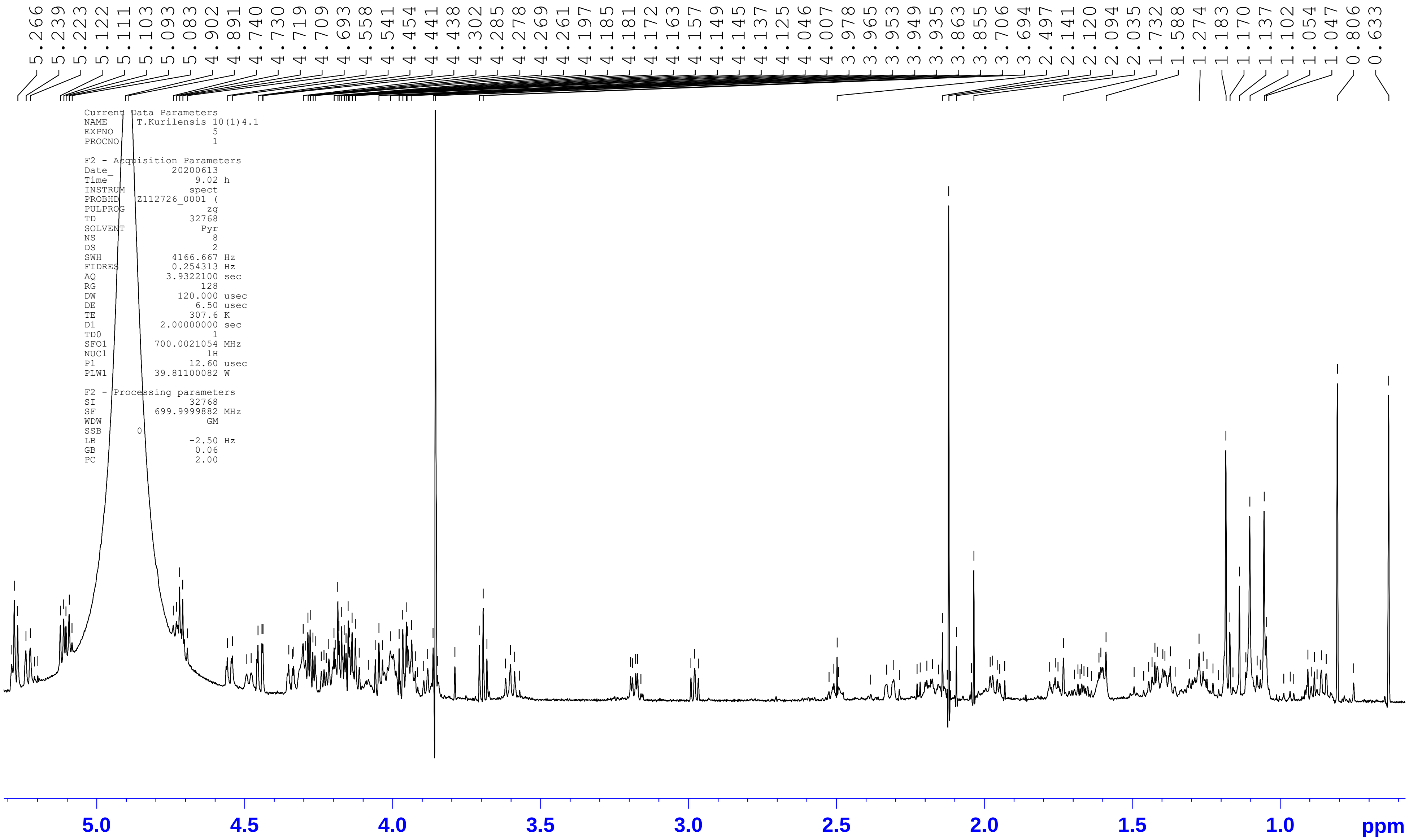

Figure S33. The ^{1}H NMR (700.00 MHz) spectrum of kuriloside E (**5**) in C_5D_5N/D_2O (4/1)

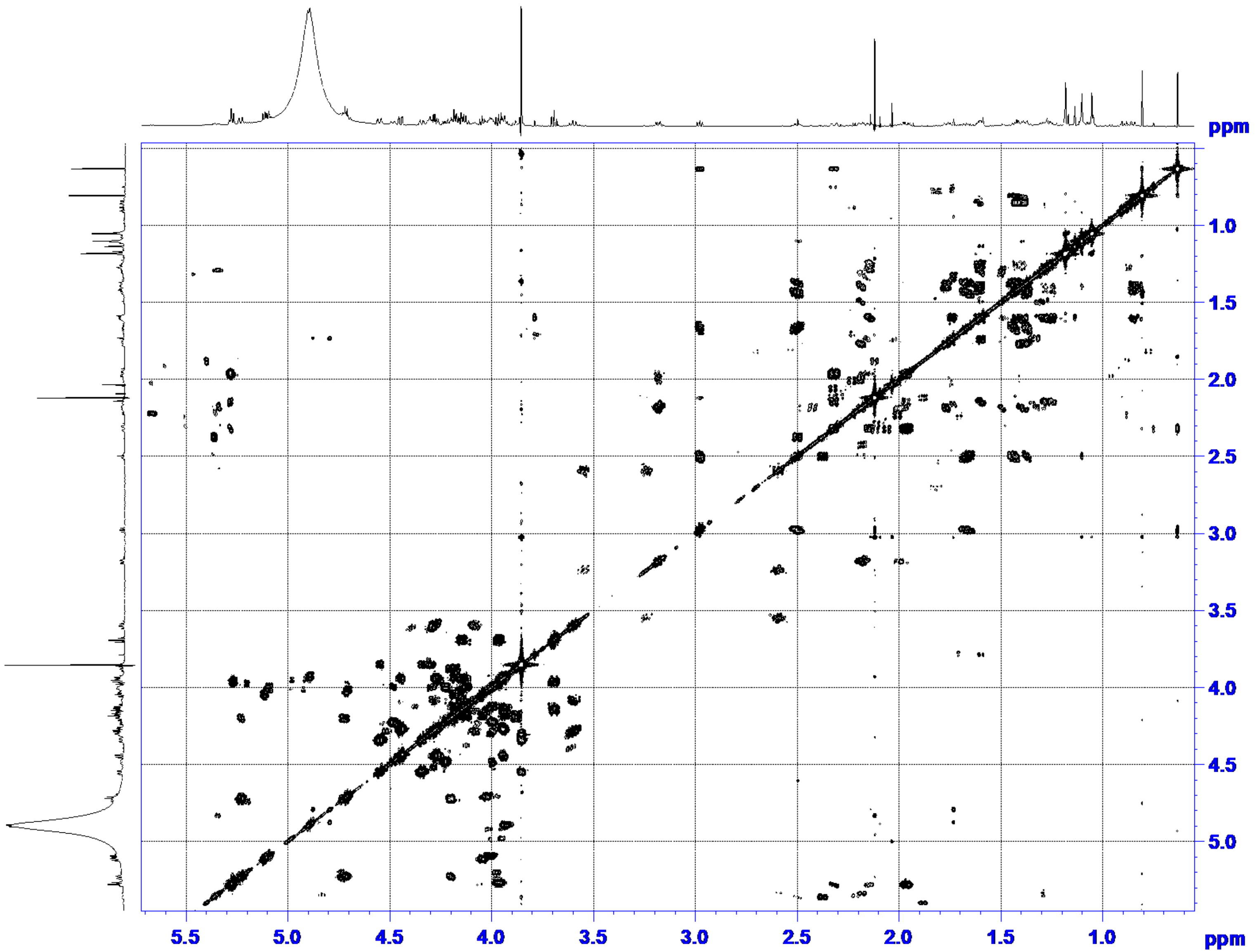

Figure S34. The COSY (700.00 MHz) spectrum of kuriloside E (**5**) in C_5D_5N/D_2O (4/1)

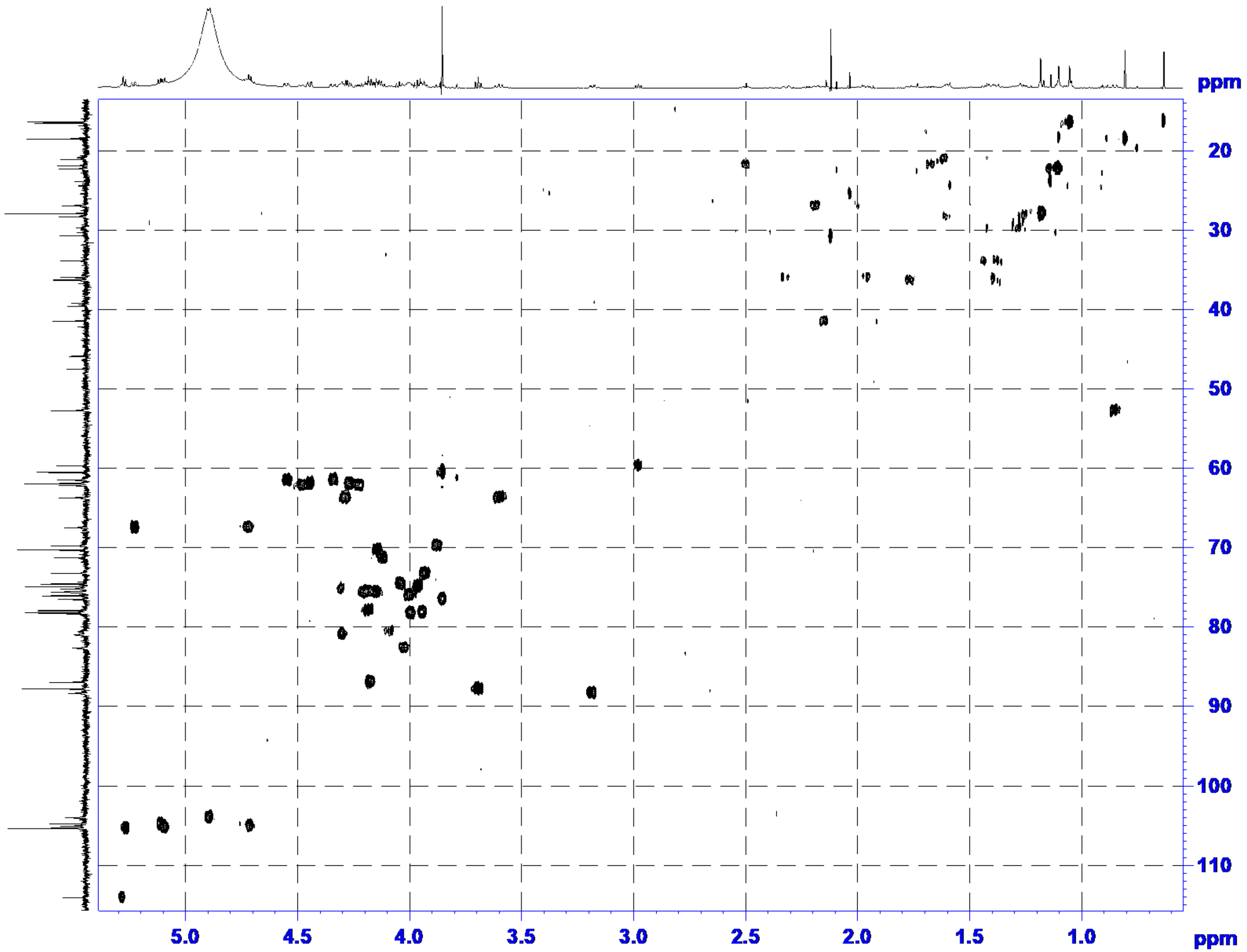

Figure S35. The HSQC (700.00 MHz) spectrum of kuriloside E (**5**) in C_5D_5N/D_2O (4/1)

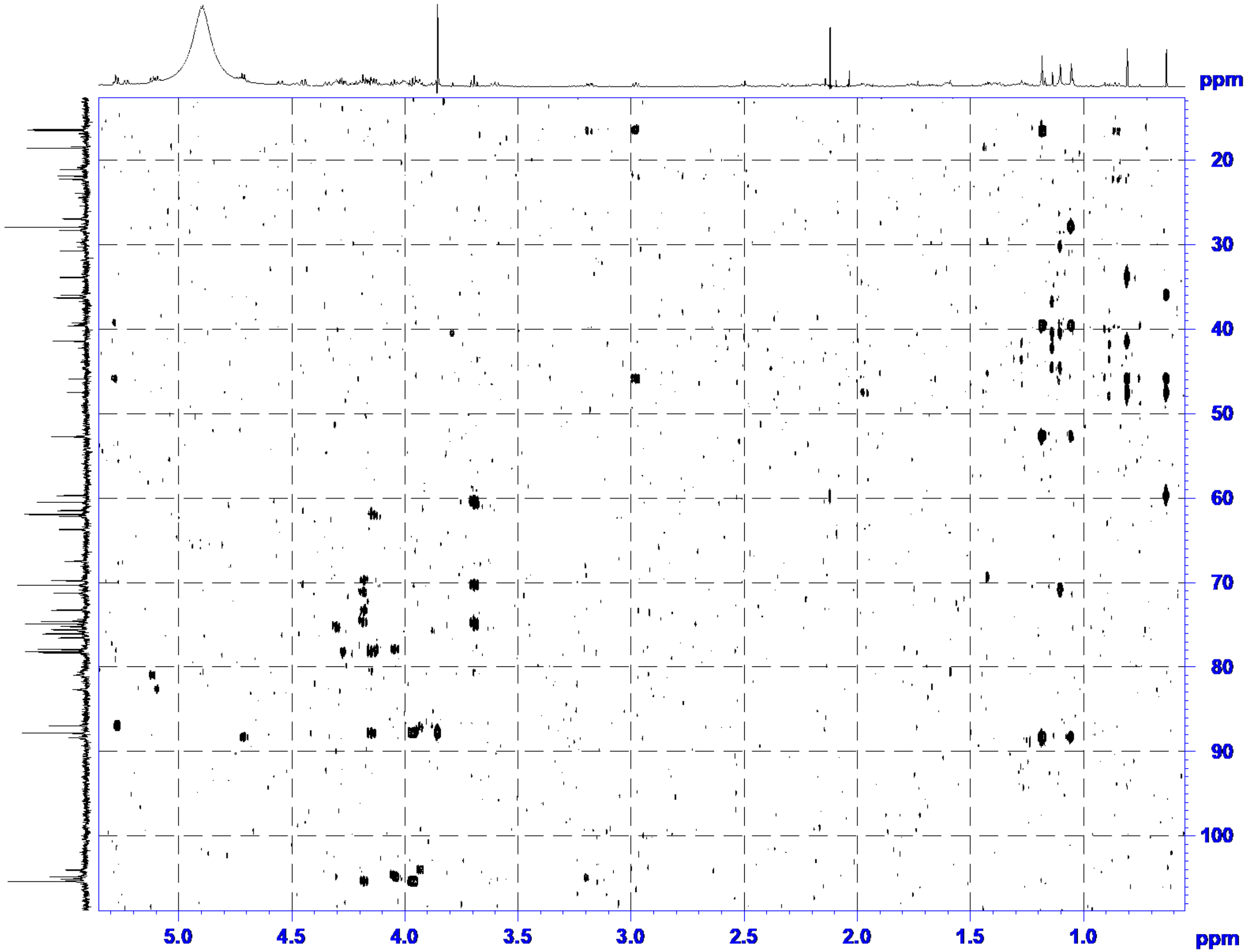

Figure S36. The HMBC (700.00 MHz) spectrum of kuriloside E (**5**) in C_5D_5N/D_2O (4/1)

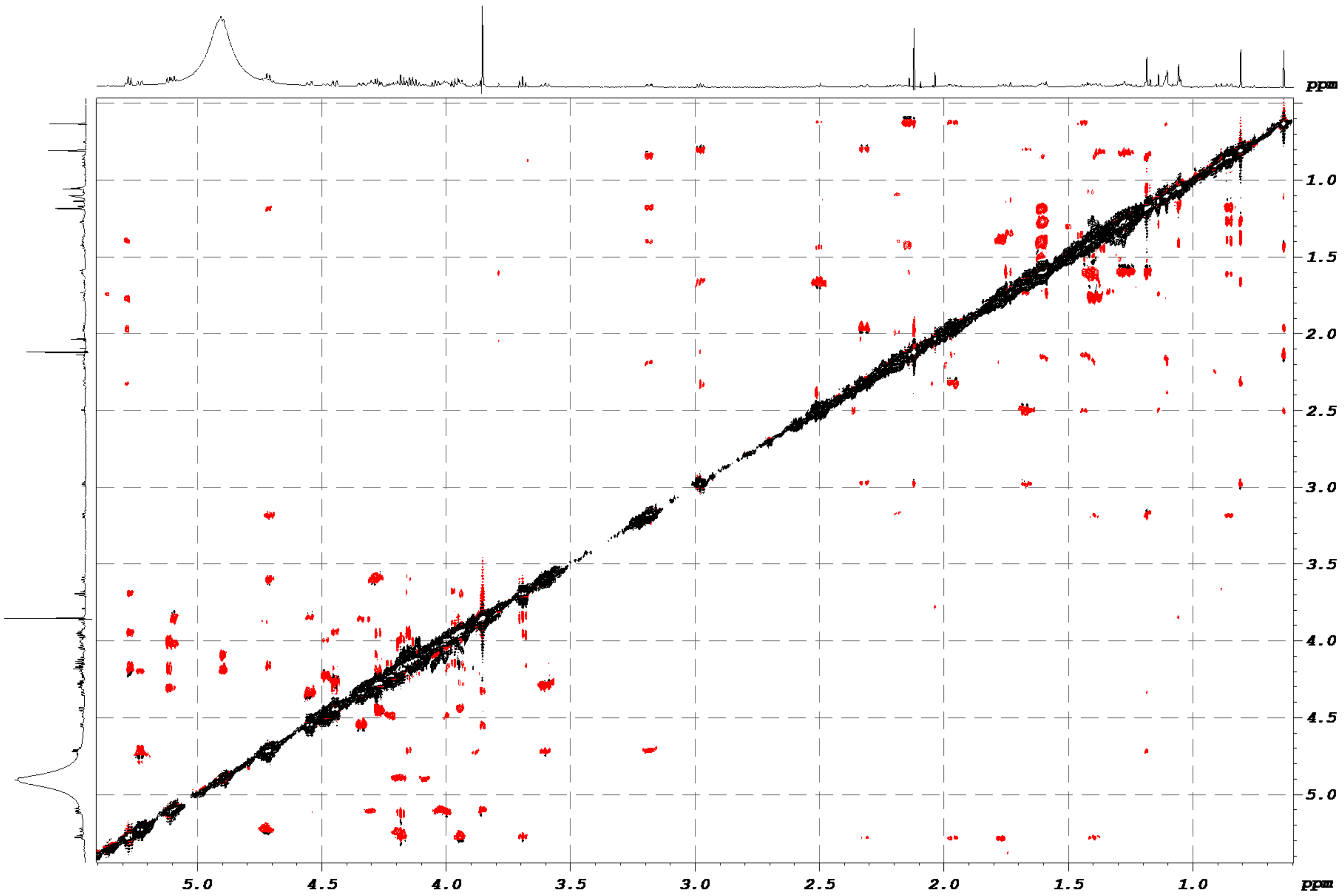

Figure S37. The ROESY (700.00 MHz) spectrum of kuriloside E (**5**) in C_5D_5N/D_2O (4/1)

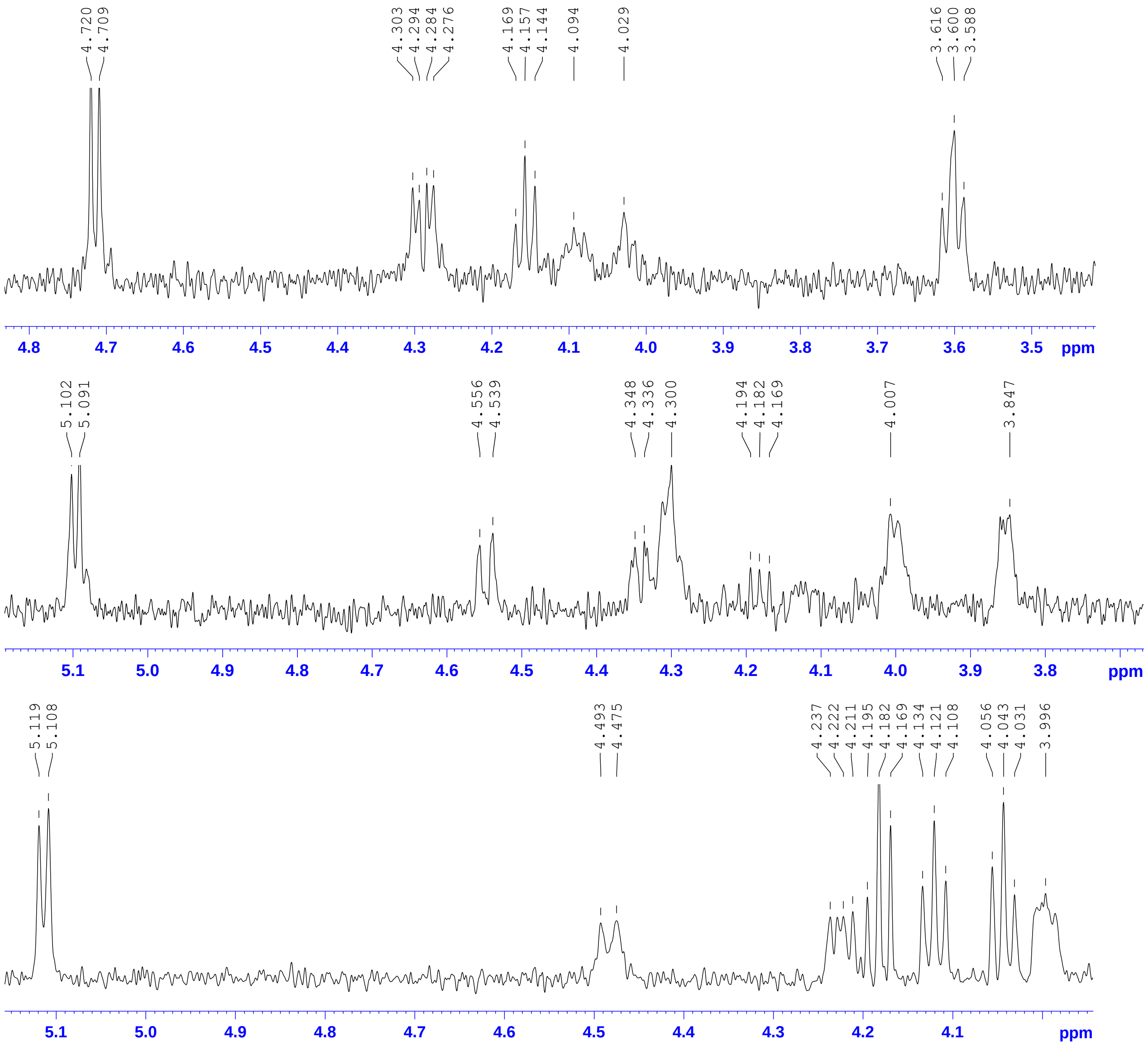

Figure S38. 1D TOCSY (700.00 MHz) spectra of kuriloside E (**5**) in C_5D_5N/D_2O (4/1)

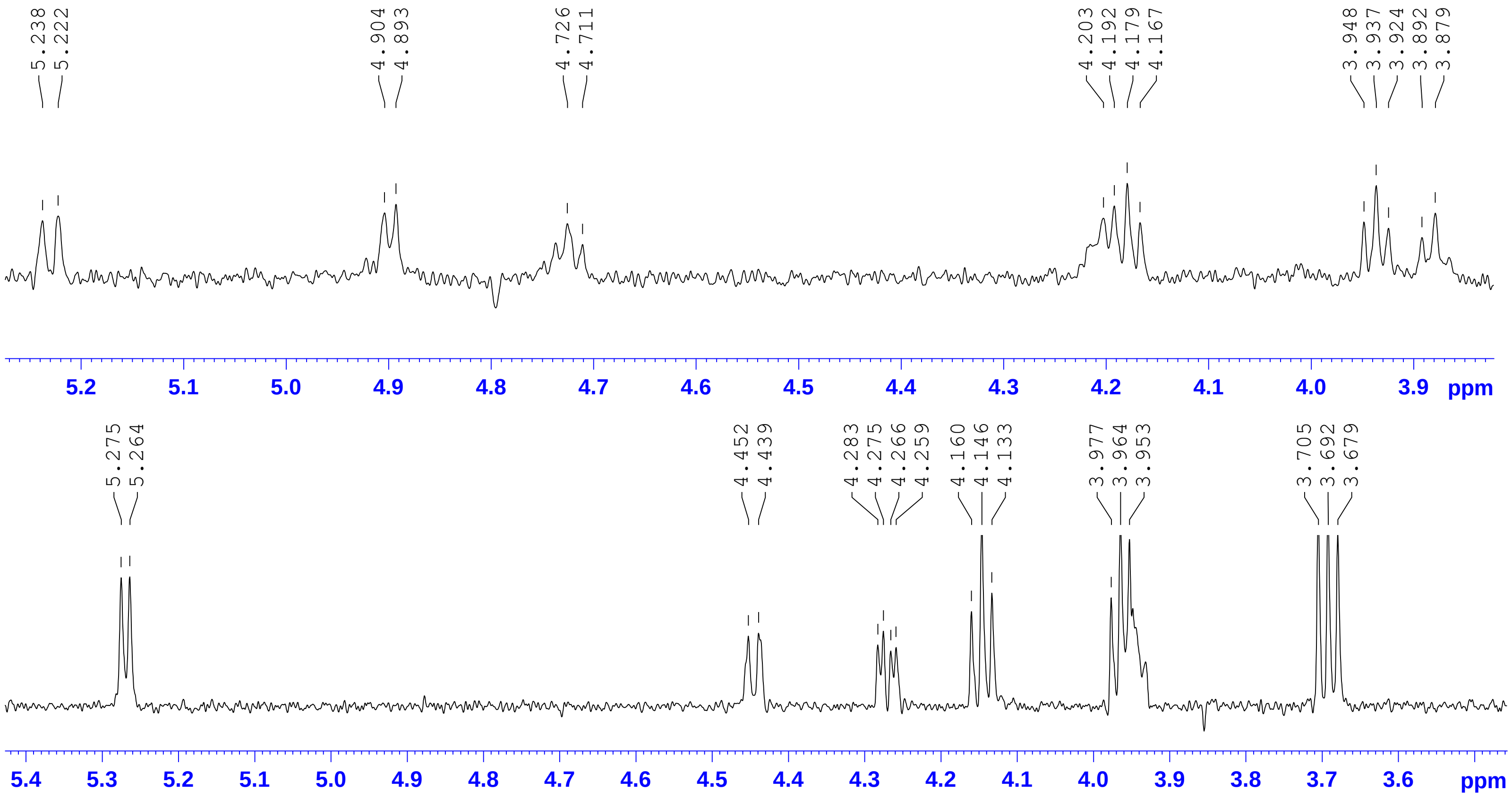

Figure S39. 1D TOCSY (700.00 MHz) spectra of kuriloside E (**5**) in C_5D_5N/D_2O (4/1)

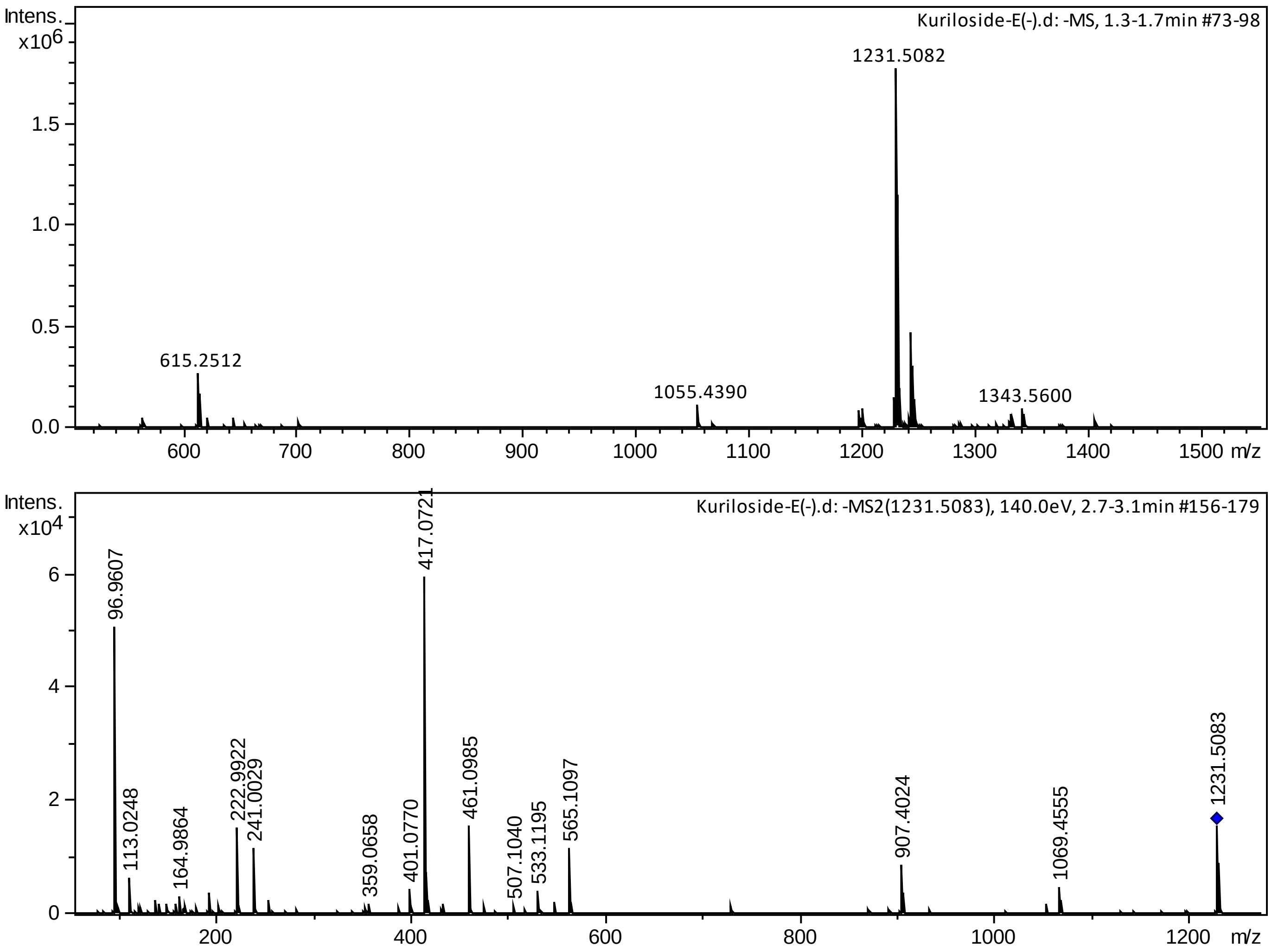

Figure S40. HR-ESI-MS and ESI-MS/MS spectra of kuriloside E (**5**)

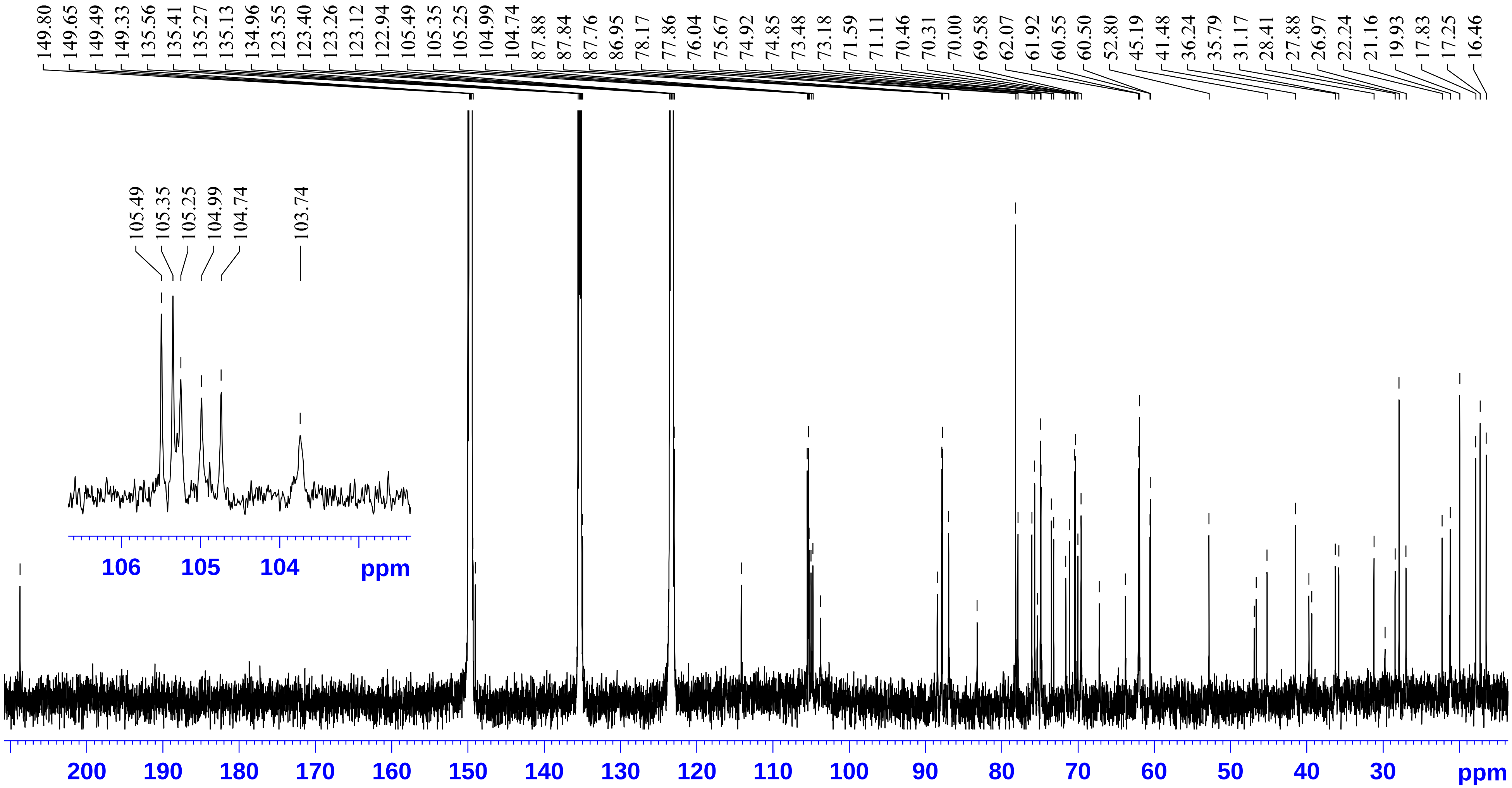

Figure S41. The ^{13}C NMR (176.03 MHz) spectrum of kuriloside F (**6**) in C_5D_5N/D_2O (4/1)

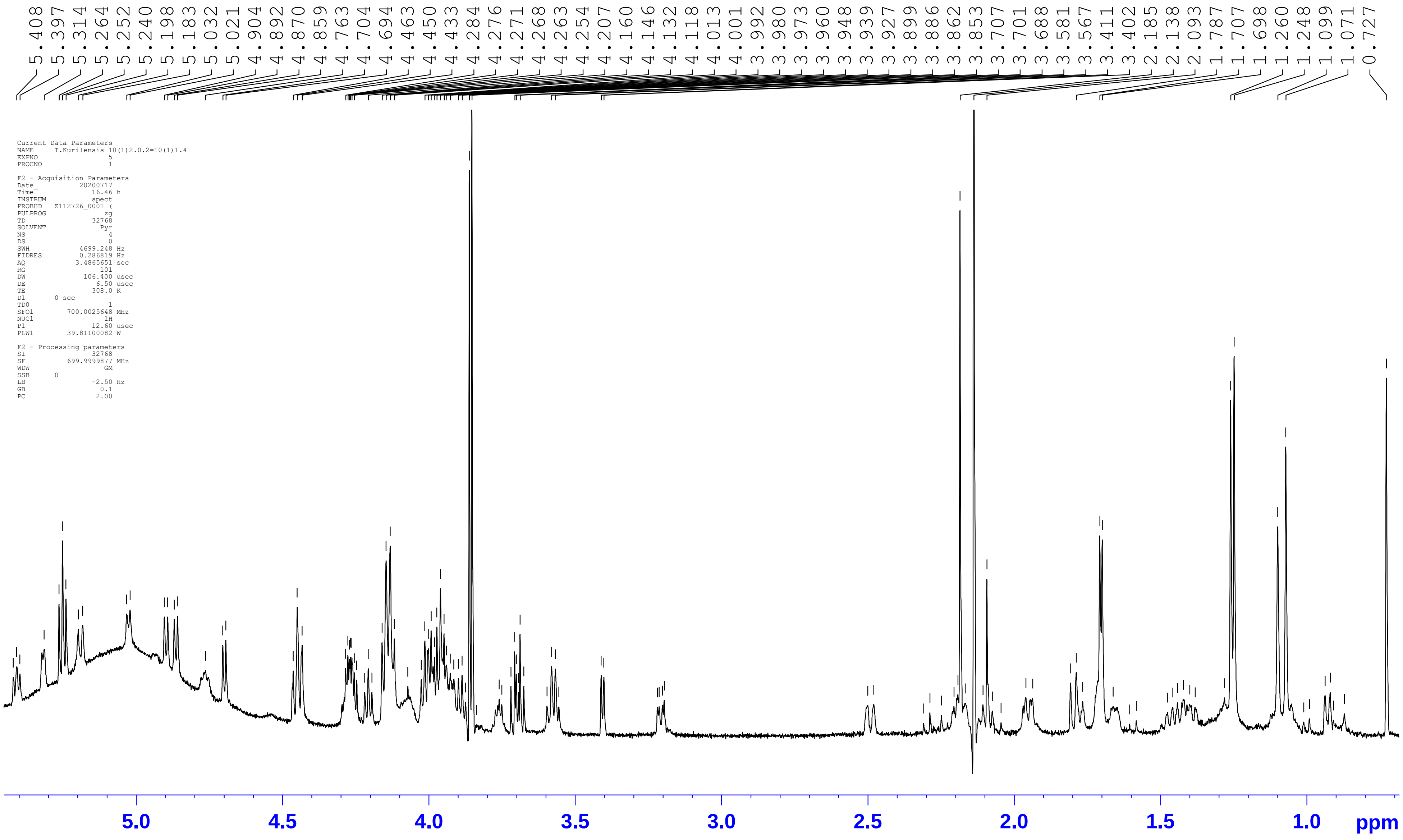

Figure S42. The ^{1}H NMR (700.00 MHz) spectrum of kuriloside F (**6**) in C_5D_5N/D_2O (4/1)

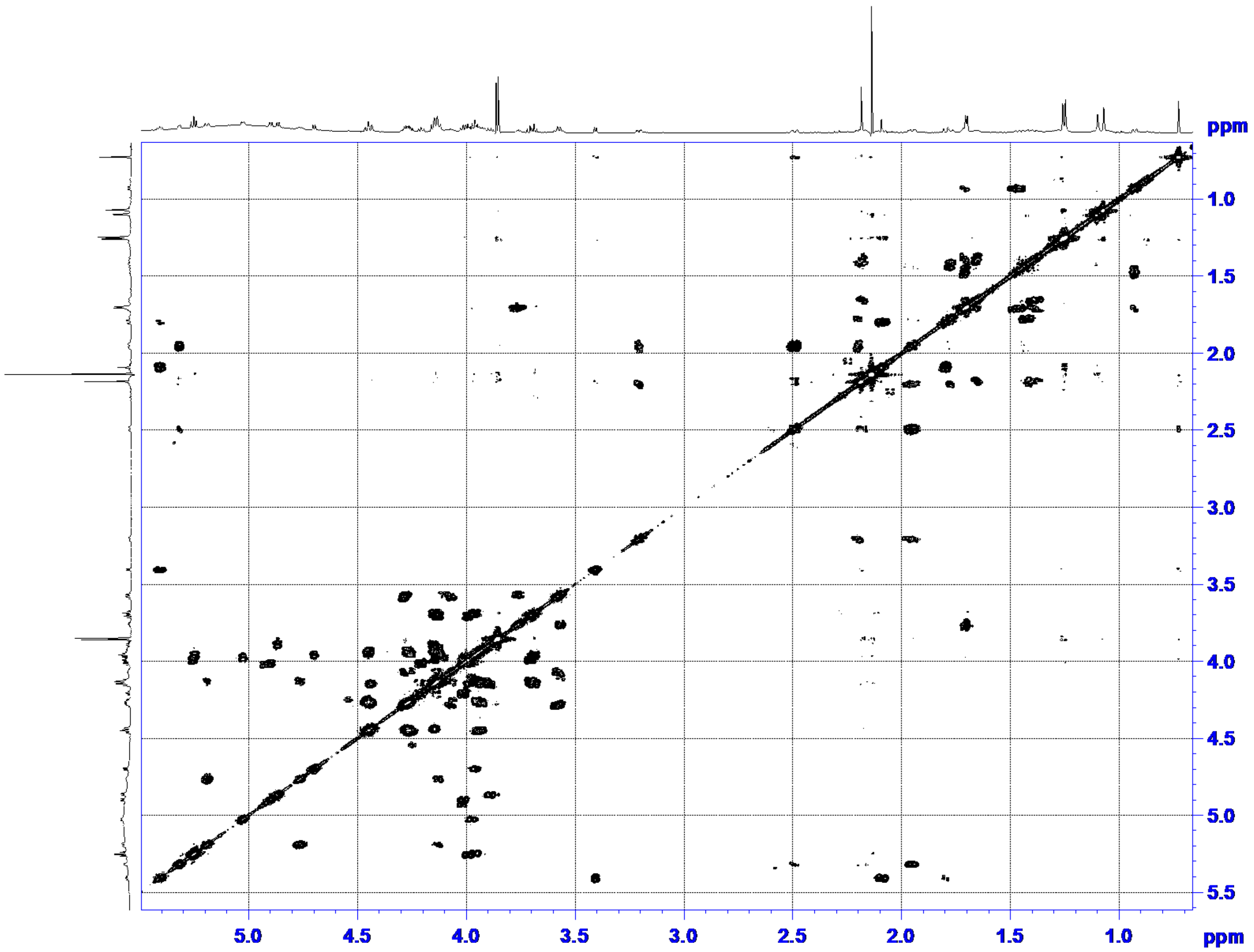

Figure S43. The COSY (700.00 MHz) spectrum of kuriloside F (**6**) in C_5D_5N/D_2O (4/1)

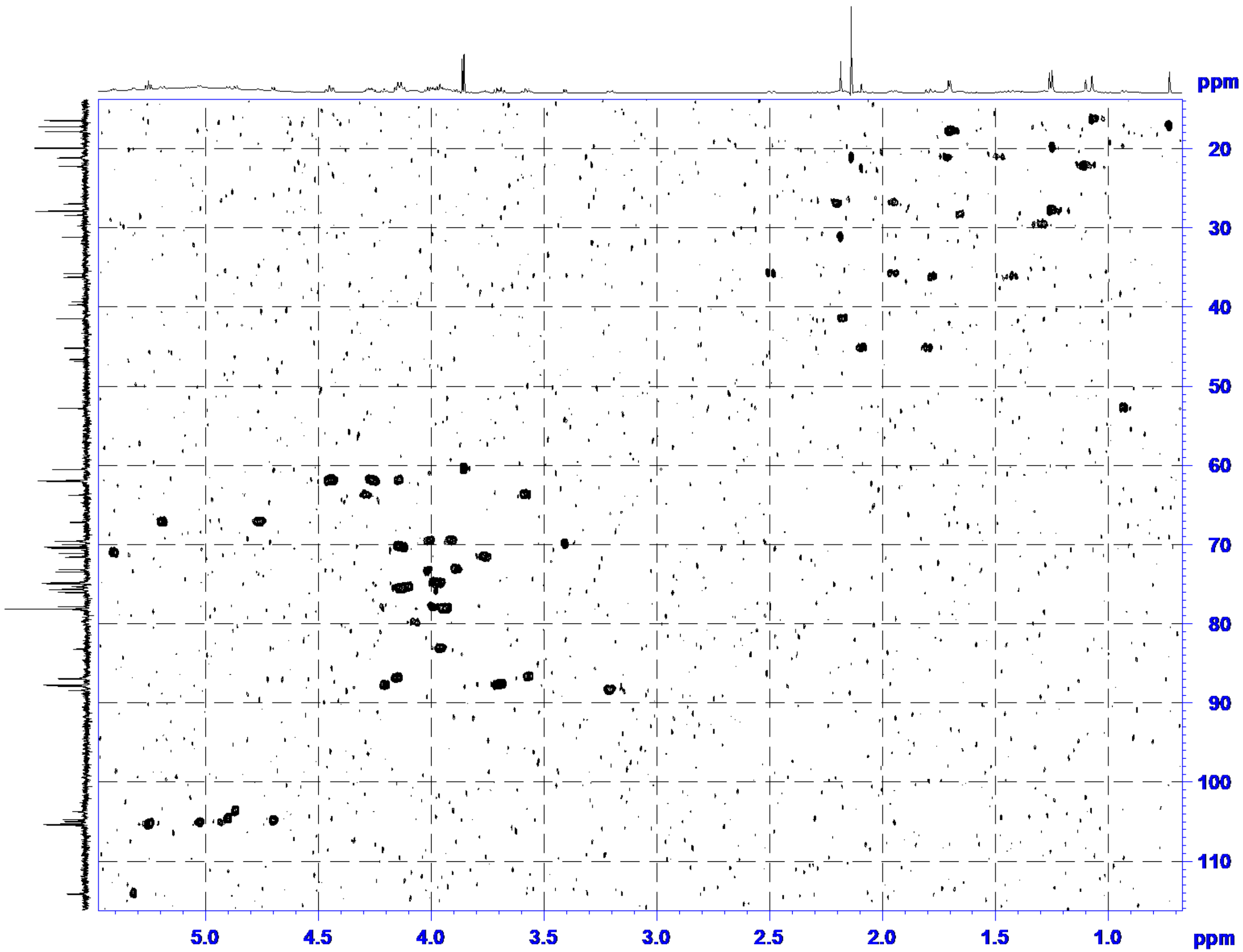

Figure S44. The HSQC (700.00 MHz) spectrum of kuriloside F (**6**) in C_5D_5N/D_2O (4/1)

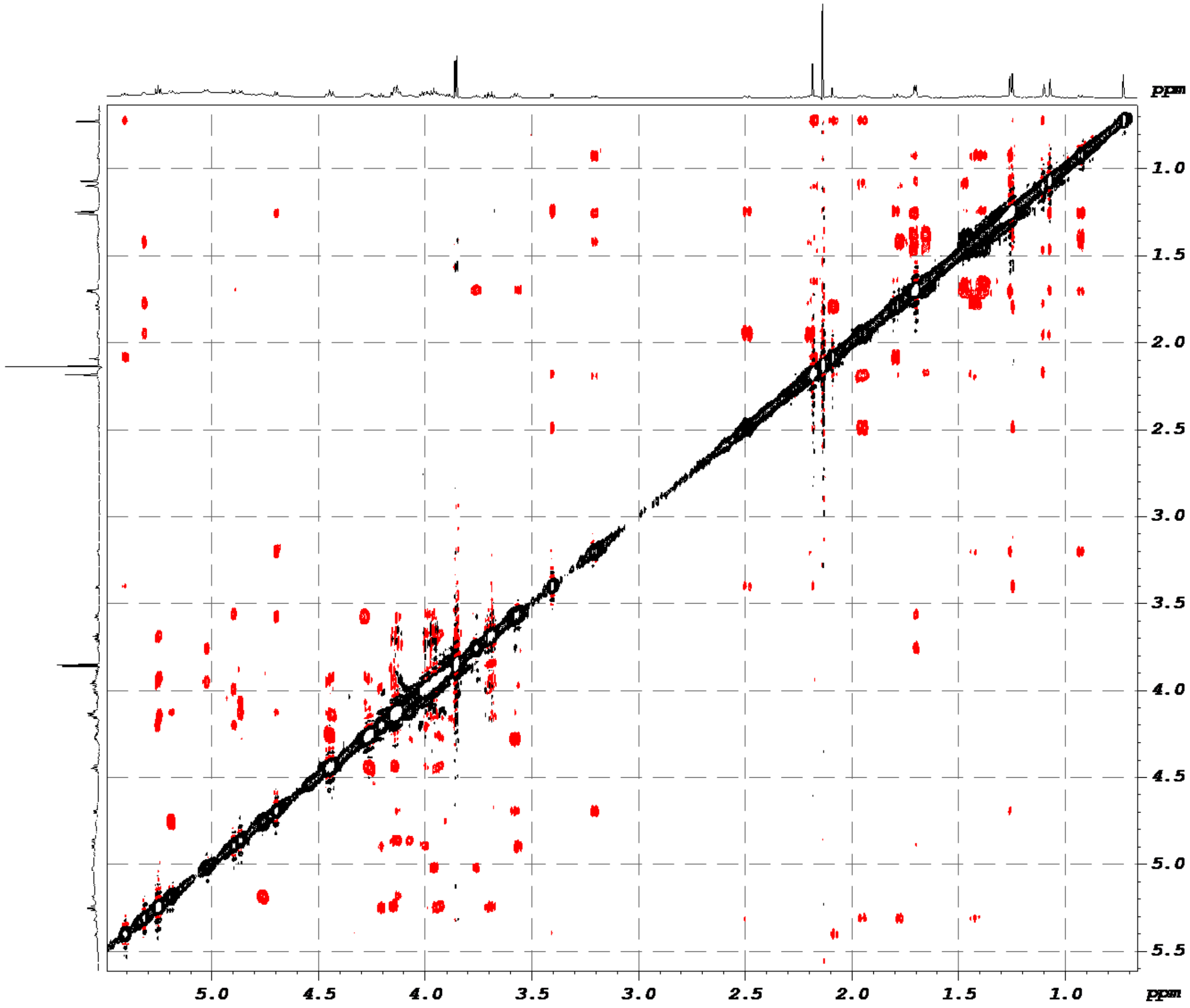

Figure S45. The ROESY (700.00 MHz) spectrum of kuriloside F (**6**) in C_5D_5N/D_2O (4/1)

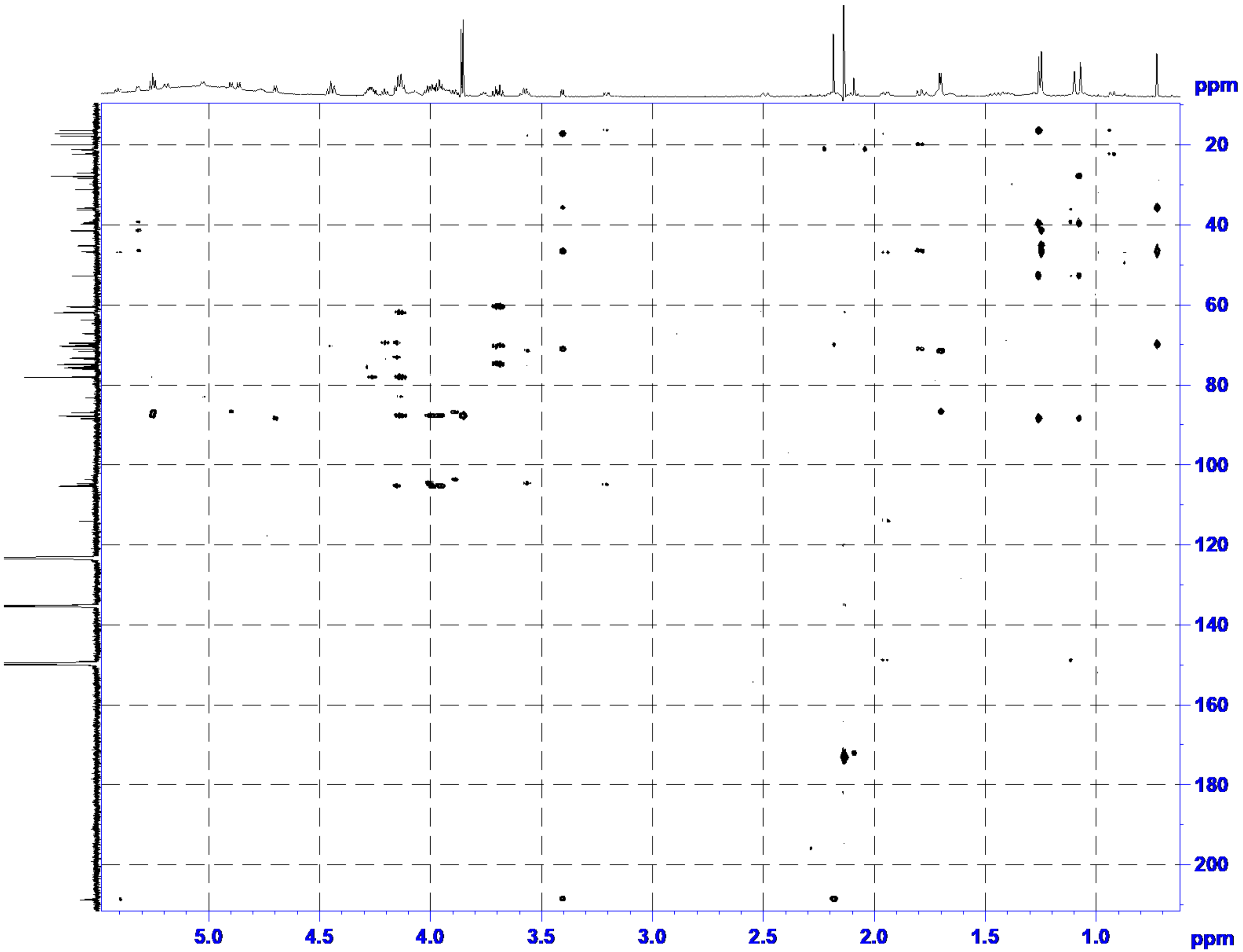

Figure S46. The HMBC (700.00 MHz) spectrum of kuriloside F (**6**) in C_5D_5N/D_2O (4/1)

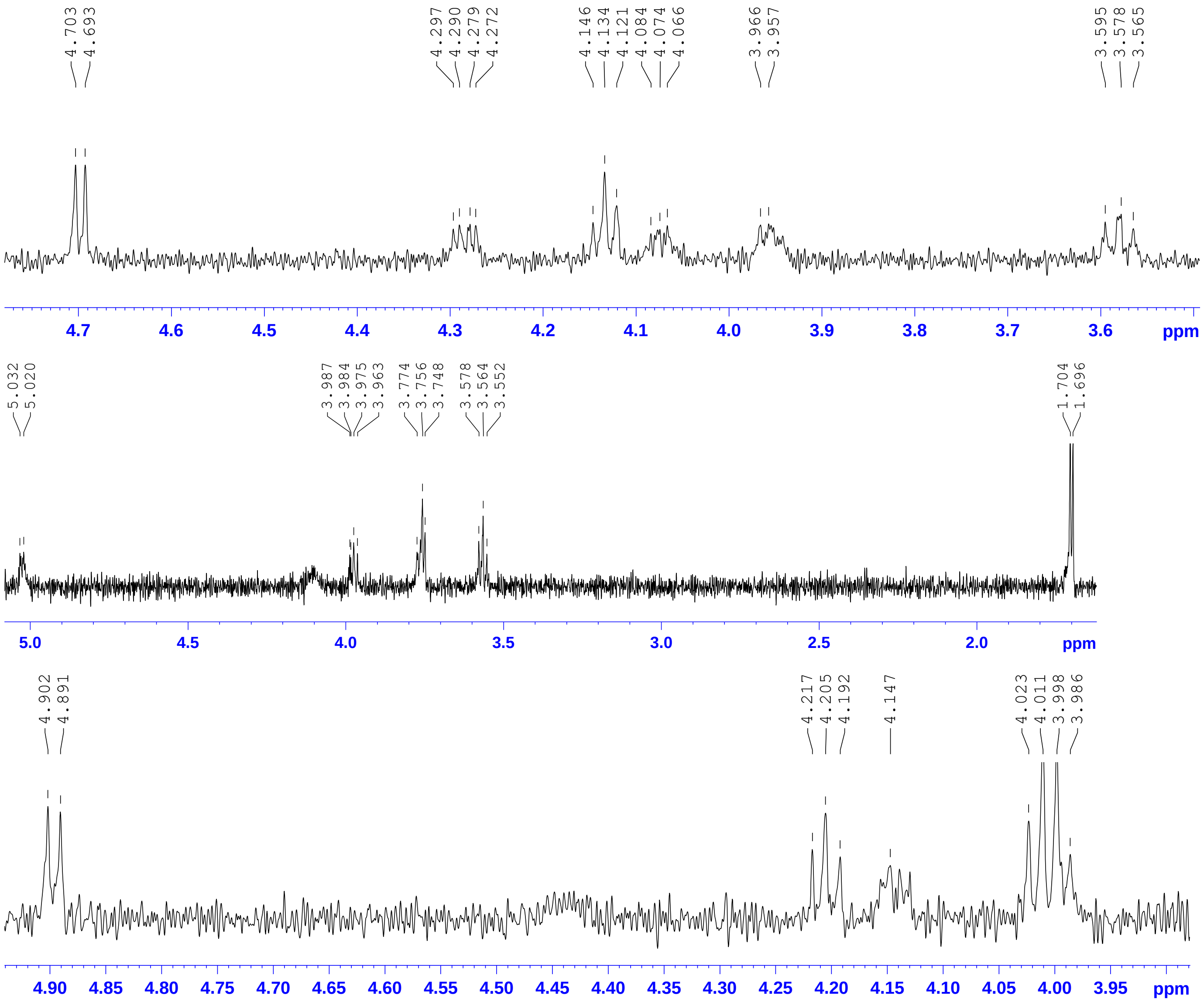

Figure S47. 1D TOCSY (700.00 MHz) spectra of the carbohydrate part of kuriloside F (**6**) in C_5D_5N/D_2O (4/1)

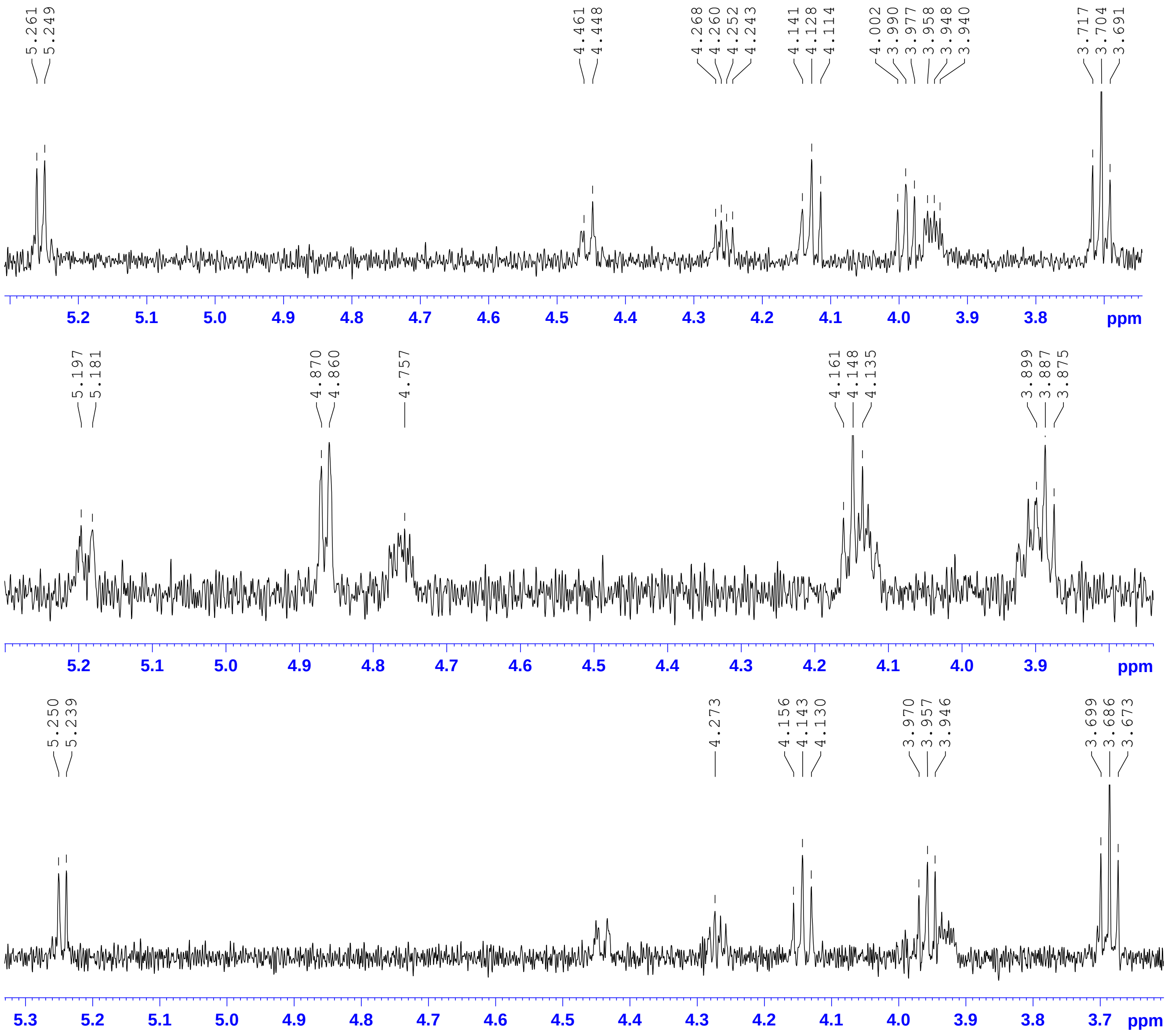

Figure S48. 1D TOCSY (700.00 MHz) spectra of the carbohydrate part of kuriloside F (**6**) in C_5D_5N/D_2O (4/1)

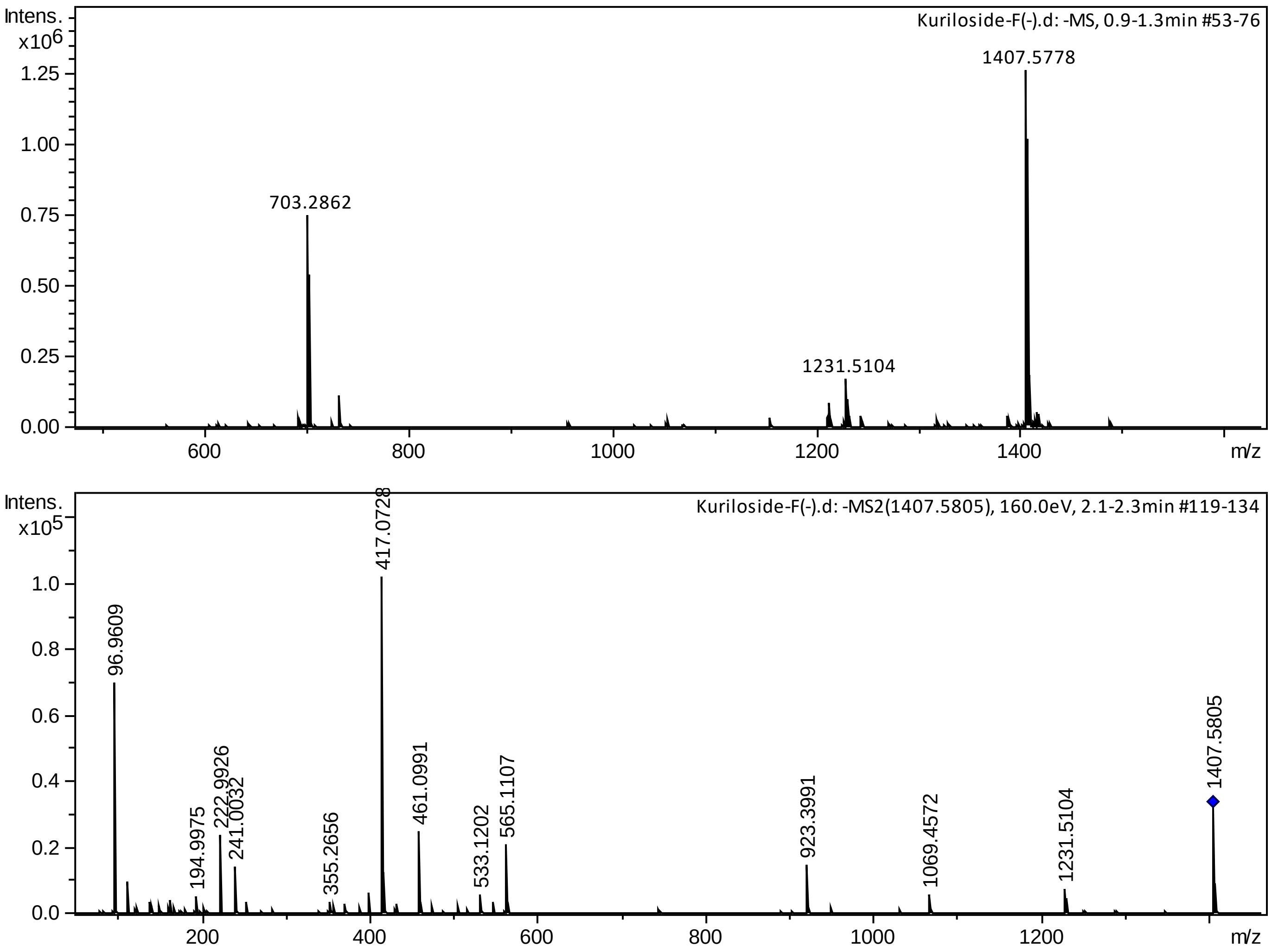

Figure S49. HR-ESI-MS and ESI-MS/MS spectra of kuriloside F (**6**)

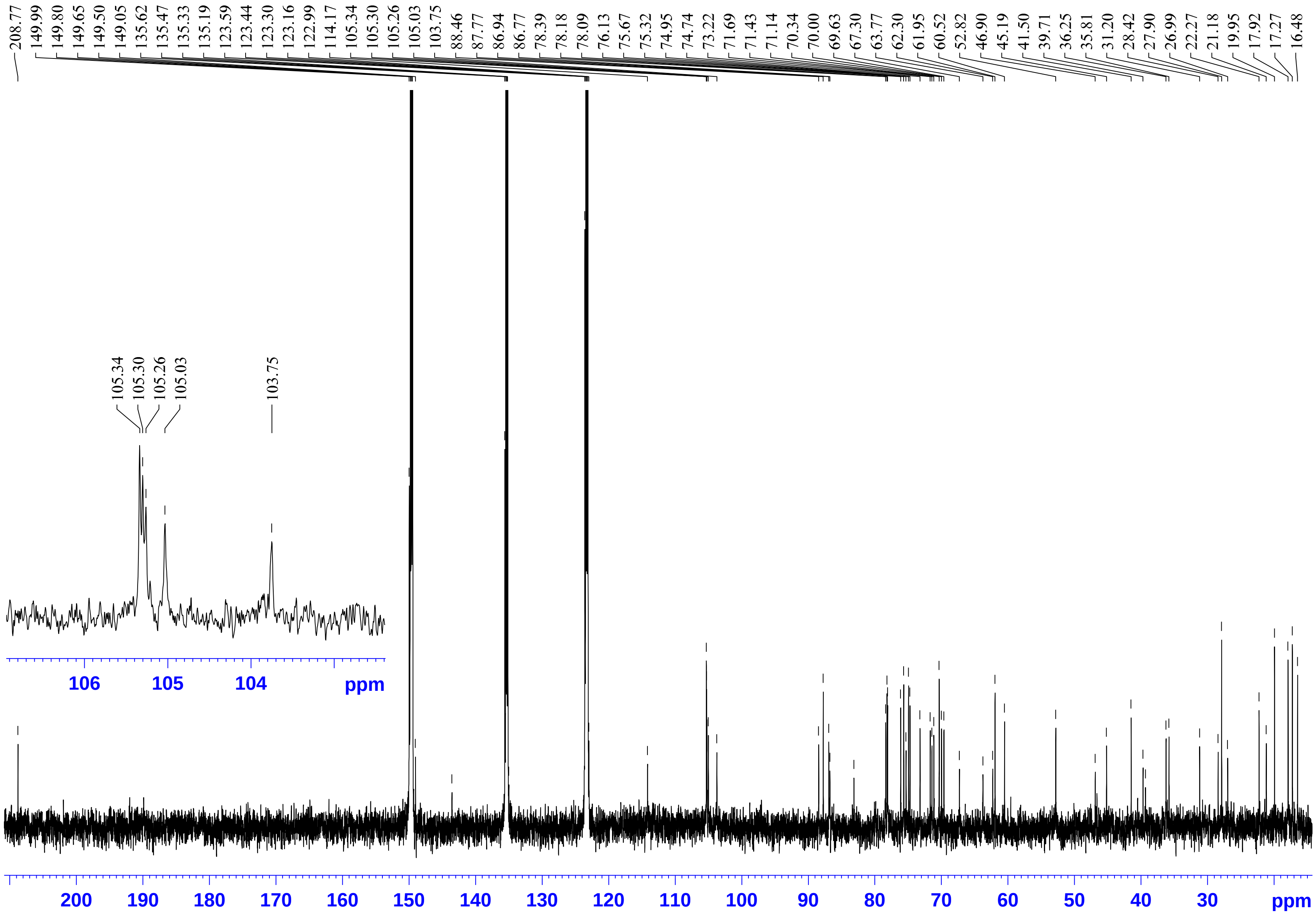

Figure S50. The ^{13}C NMR (176.03 MHz) spectrum of kuriloside A (**7**) in C_5D_5N/D_2O (4/1)

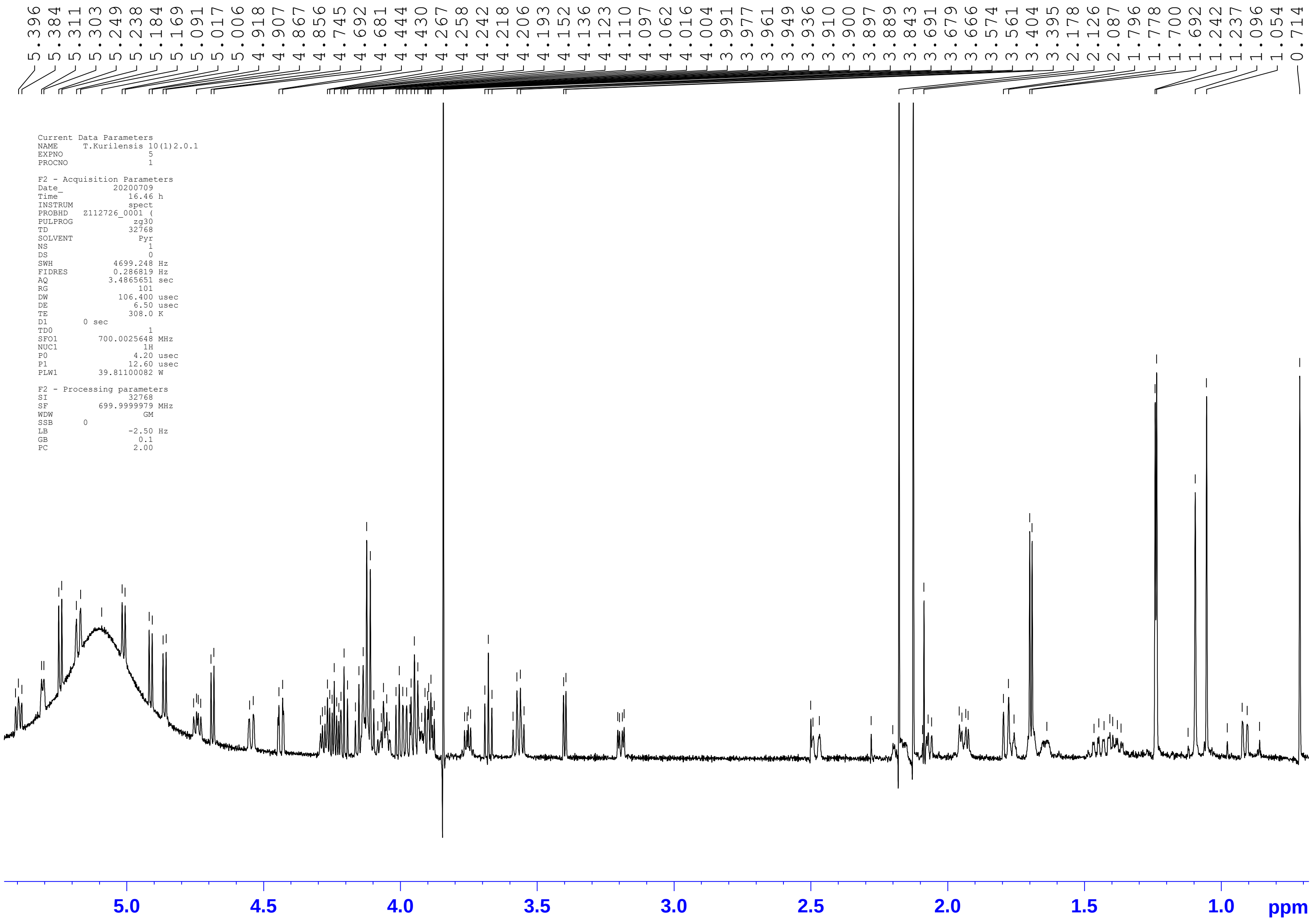

Figure S51. The ^1H NMR (700.00 MHz) spectrum of kuriloside A (**7**) in C_5D_5N/D_2O (4/1)

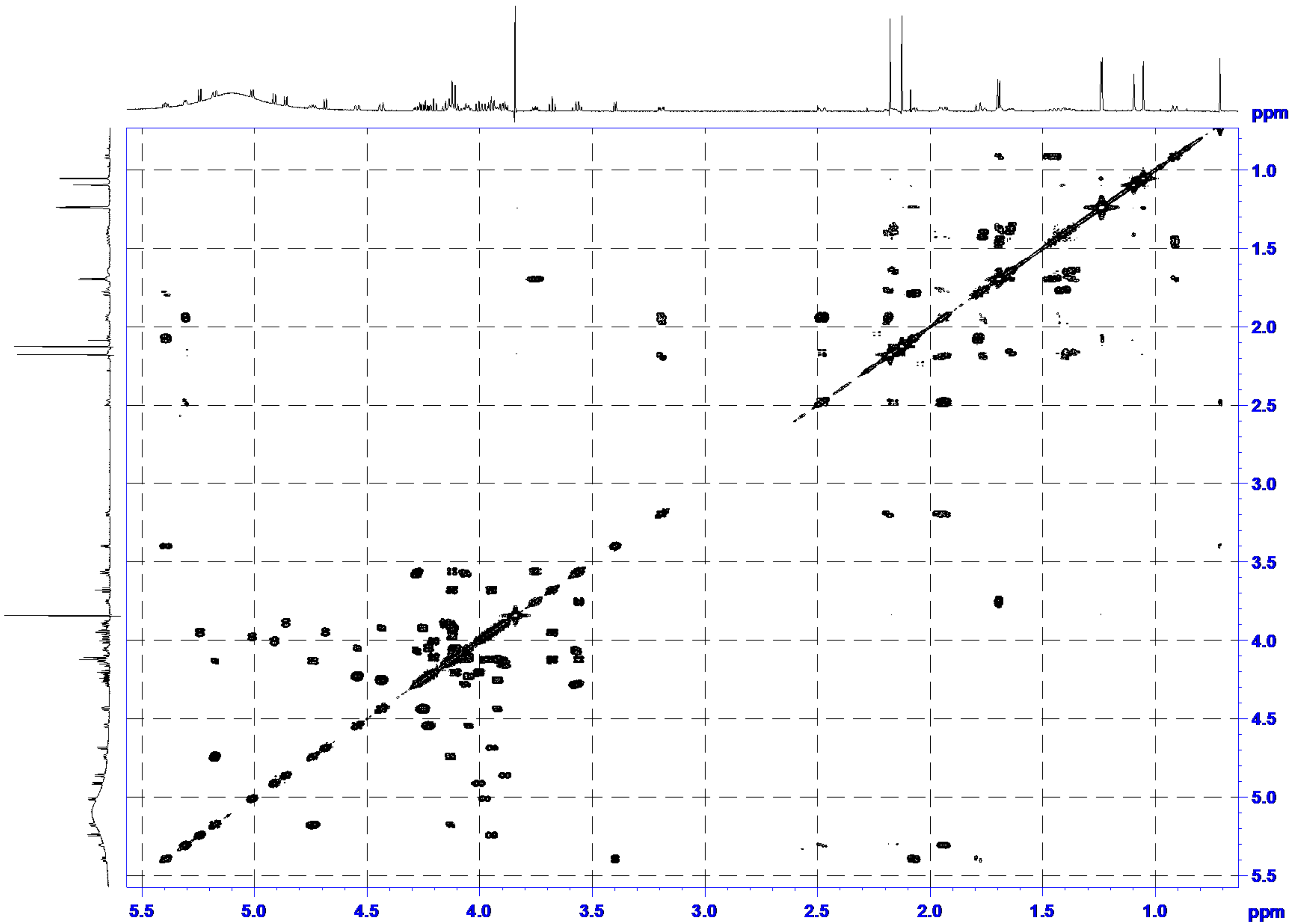

Figure S52. The COSY (700.00 MHz) spectrum of kuriloside A (**7**) in C_5D_5N/D_2O (4/1)

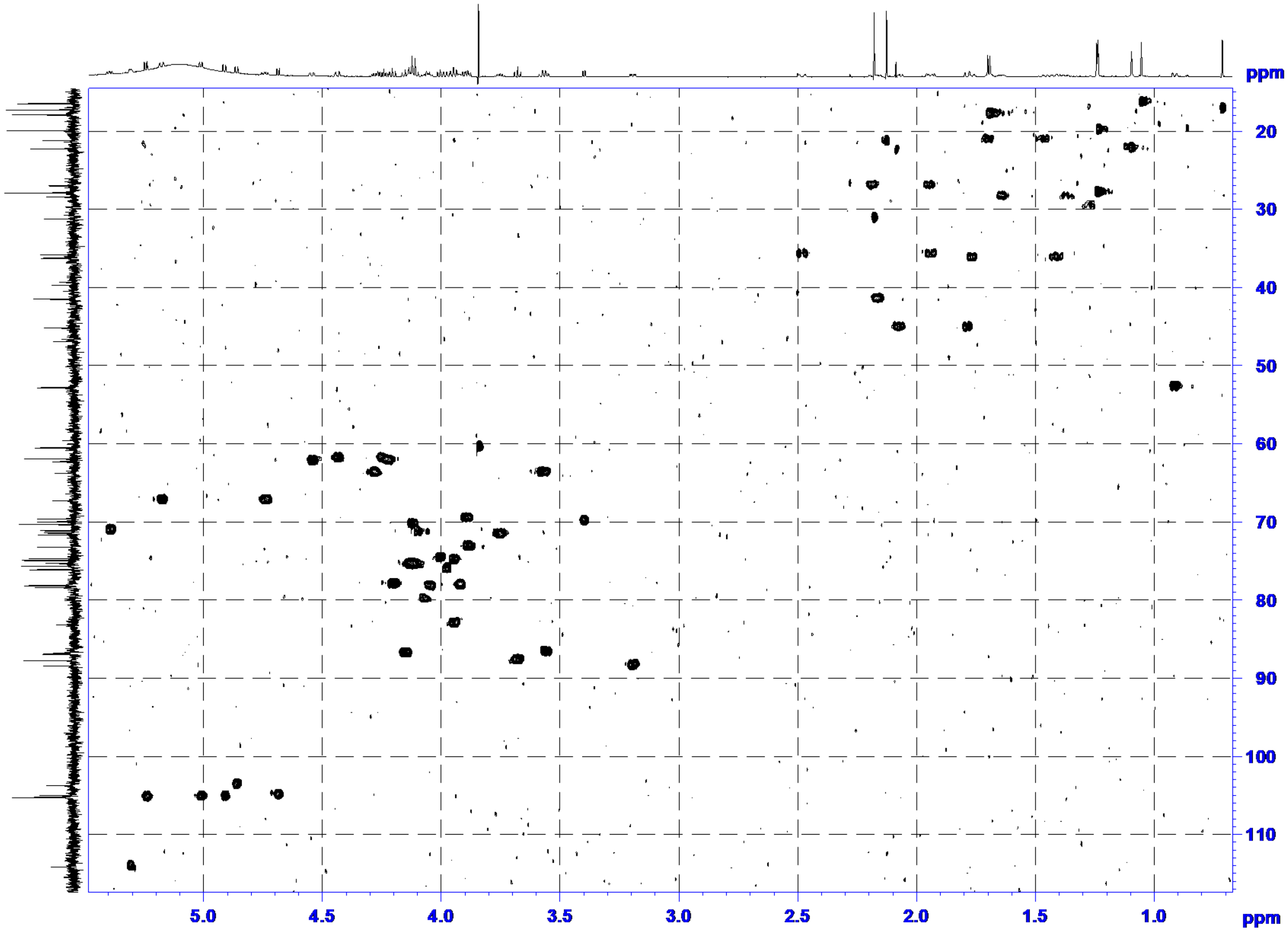

Figure S53. The HSQC (700.00 MHz) spectrum of kuriloside A (**7**) in C_5D_5N/D_2O (4/1)

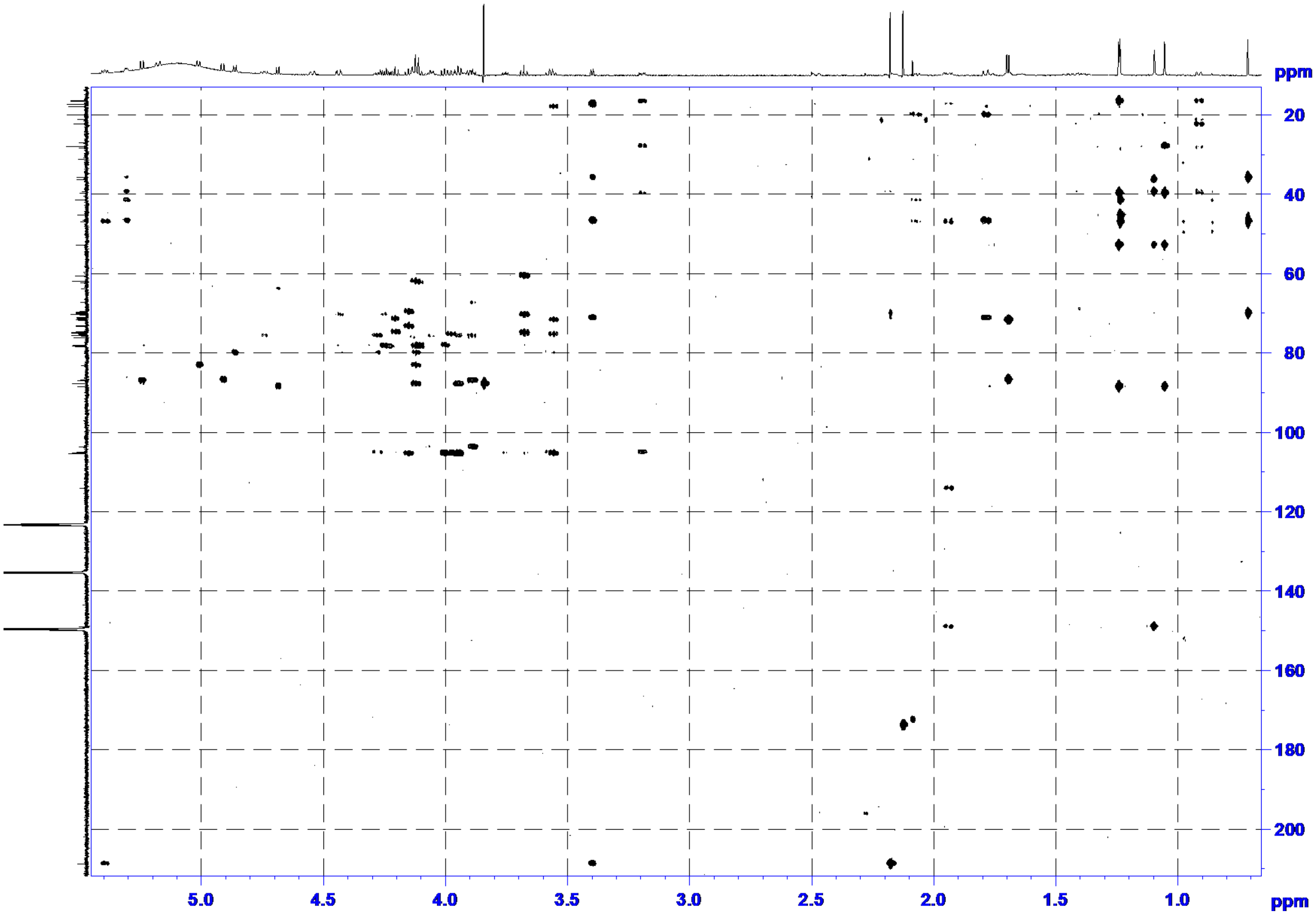

Figure S54. The HMBC (700.00 MHz) spectrum of kuriloside A (**7**) in C_5D_5N/D_2O (4/1)

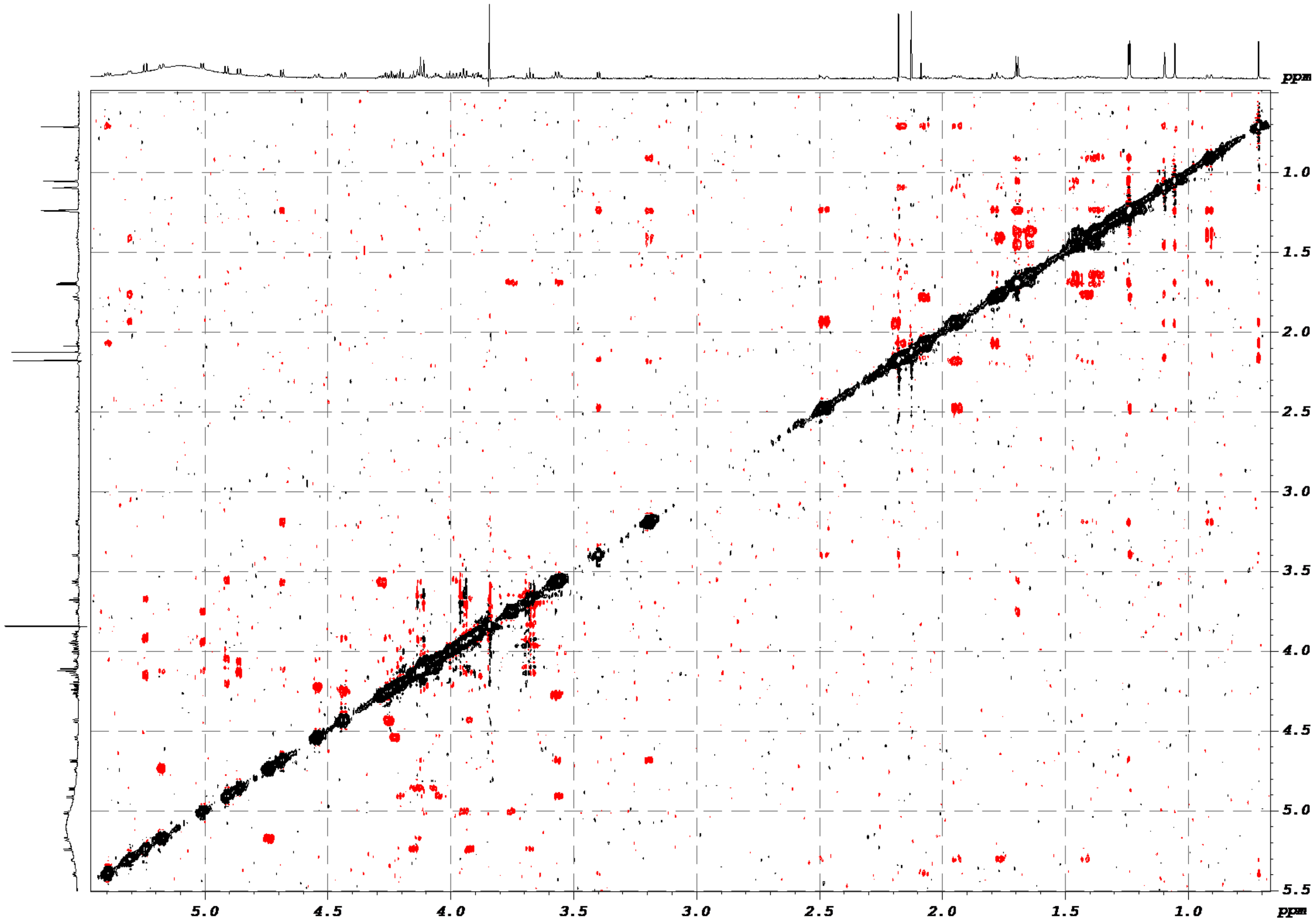

Figure S55. The ROESY (700.00 MHz) spectrum of kuriloside A (**7**) in C_5D_5N/D_2O (4/1)

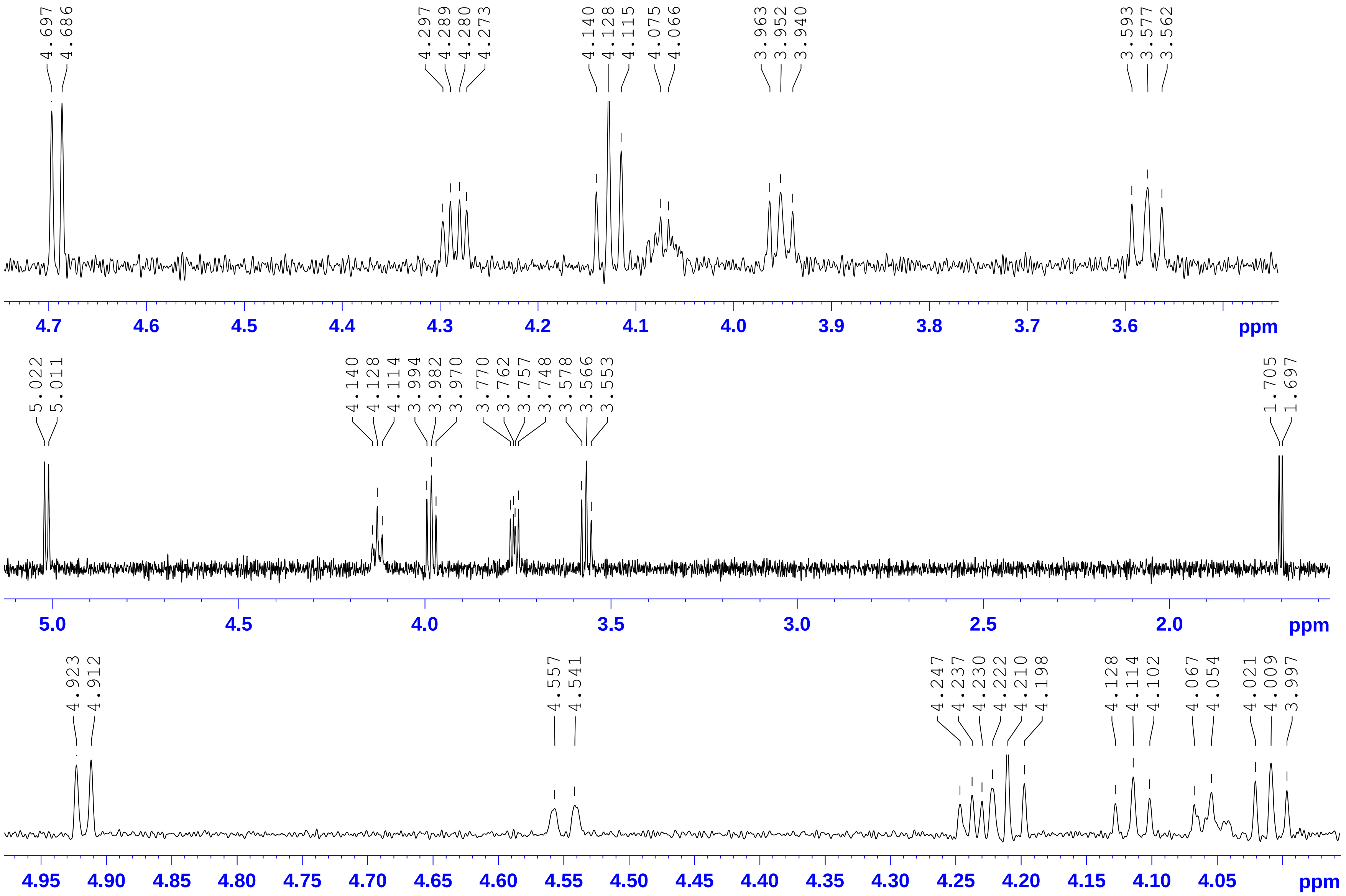

Figure S56. 1 D TOCSY (700.00 MHz) spectra of kuriloside A (**7**) in C_5D_5N/D_2O (4/1)

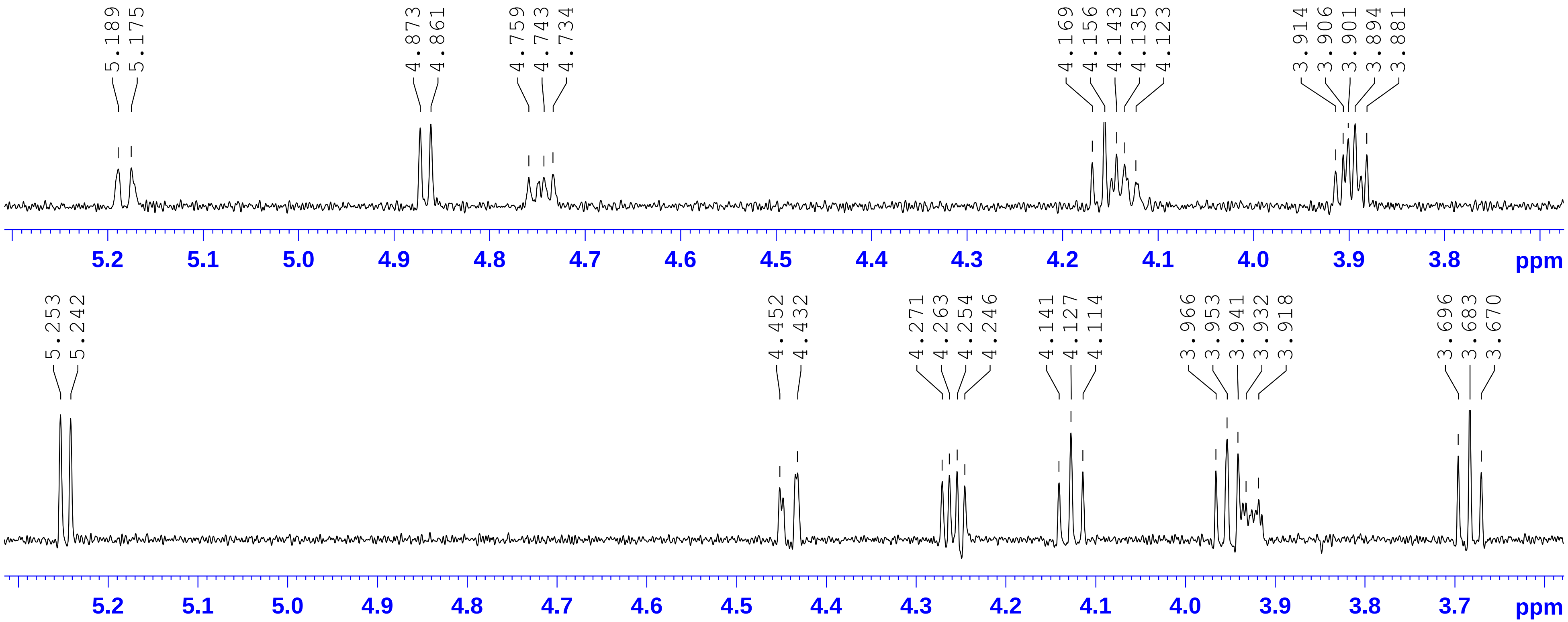

Figure S57. 1 D TOCSY (700.00 MHz) spectra of kuriloside A (**7**) in C_5D_5N/D_2O (4/1)

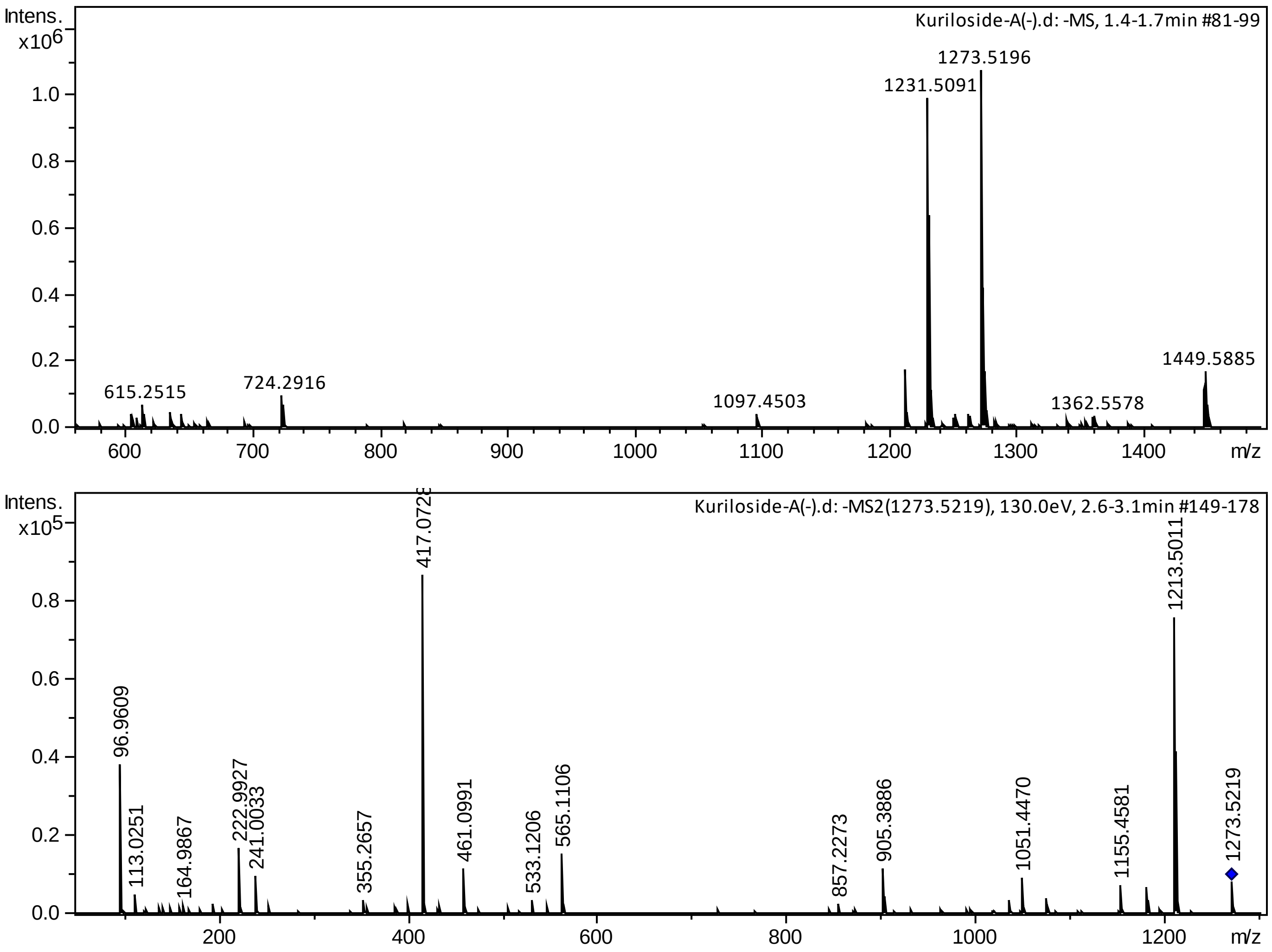

Figure S58. HR-ESI-MS and ESI-MS/MS spectra of kuriloside A (**7**)
